# ﻿Land snail diversity in central China: revision of *Laeocathaica* Möllendorff, 1899 (Gastropoda, Camaenidae), with descriptions of seven new species

**DOI:** 10.3897/zookeys.1154.86237

**Published:** 2023-03-20

**Authors:** Min Wu, Wang Shen, Zhong-Guang Chen

**Affiliations:** 1 School of Life Sciences, Nanjing University, Nanjing 210023, China Nanjing University Nanjing China; 2 Jiangxi Province Key Laboratory of Watershed Ecosystem Change and Biodiversity, Center for Watershed Ecology, Institute of Life Science and School of Life Sciences, Nanchang University, Nanchang 330031, China Nanchang University Nanchang China

**Keywords:** Anatomy, Central China, Helicoidea, molecular phylogeny, morphology, taxonomy

## Abstract

Central China harbors the native dart-sac-bearing camaenids *Laeocathaica*. The genus is revised and seven new species are proposed based on museum material and newly collected specimens. This work confirmed that most *Laeocathaica* species have restricted habitats. The comparison of the dart sac apparatus among the dart-sac-bearing camaenid genera indicated the importance of the presence of the proximal accessory sac that might be analogous to the membranous/muscular sac surrounding the proximal dart sac and/or the distal region of the vagina near the atrium, which also plays a significant role in the diagnosis of *Laeocathaica* species based on its number, symmetry and position on the dart sac. Species with similar shell morphology were studied using geometric morphometric methods to detect variations in shell shape. A molecular phylogenetic analysis based on 16S and ITS2 sequence data of partial *Laeocathaica* species and many other dart-sac-bearing taxa suggested that *Laeocathaica* might be monophyletic. Furthermore, the present phylogeny indicated that *Stilpnodiscus*, *Cathaica*, *Bradybaena*, and *Pseudobuliminus* might be polyphyletic and therefore the taxonomy of dart-sac-bearing camaenids in this region requires a thorough revision. This work reconfirms that the Southern Gansu Plateau is important as a hotspot for malacodiversity conservation on the Chinese mainland.

## ﻿Introduction

*Laeocathaica* Möllendorff, 1899 comprises more than twenty species, most of which were described by O. von Möllendorff. It is a group of sinistrally-shelled camaenids indigenous to Central China, an area that includes western Hubei, Chongqing (administratively part of Sichuan before 1997), Sichuan, southern Gansu, and western Shaanxi (works before 1934 listed in References; Wu and Chen 1998; Chen and Zhang 2004; Páll-Gergely et al. 2022). This genus is characterized by a sinistral shell with a conic to strongly depressed spire, usually with a sharp carina, more or less reflexed aperture, broad umbilicus and weak or strong apertural barriers on the young shell. However, their shell morphological phenotypic variation is so wide that most shell characters can also be observed in other dart-sac-bearing camaenids of China, such as toothless and toothed aperture of juvenile/mature shell, rounded and carinate periphery, narrow and broad umbilicus, scaly and smooth periostracum, banded color pattern and so on, which makes us question, whether it is a monophyletic group (Páll-Gergely et al. 2022). It is noteworthy that in the original paper on the introduction of this genus Möllendorff (1899) noticed that this group might be distinguished from other related genera by “juniora labro interno munita”, which means “during postembryogenesis several sets of teeth, different from those developed at adult stage in shape and/or number, present and remained to adult stage” (Schileyko 2004). In comparison to the shell characteristics, which are not always reliable taxonomic markers, the characteristics of genitalia are considered important to the systematics of dart-sac-bearing camaenids. Unfortunately, the genital anatomy of *Laeocathaica* species described so far has been insufficiently investigated, except for very few species (Wiegmann 1900; Schileyko 2004), whose absence makes the delimitation of the genus problematic.

This paper examines the shell morphology and genital anatomy of *Laeocathaica* using museum material and recent field collections and proposes seven new species. A molecular phylogeny based on 16S and ITS2 sequence data of a subset of *Laeocathaica* species and other dart-sac-bearing taxa is constructed to explore whether *Laeocathaica* is monophyletic.

## ﻿Materials and methods

### ﻿Specimen preparation and observation

**Figure 1.**
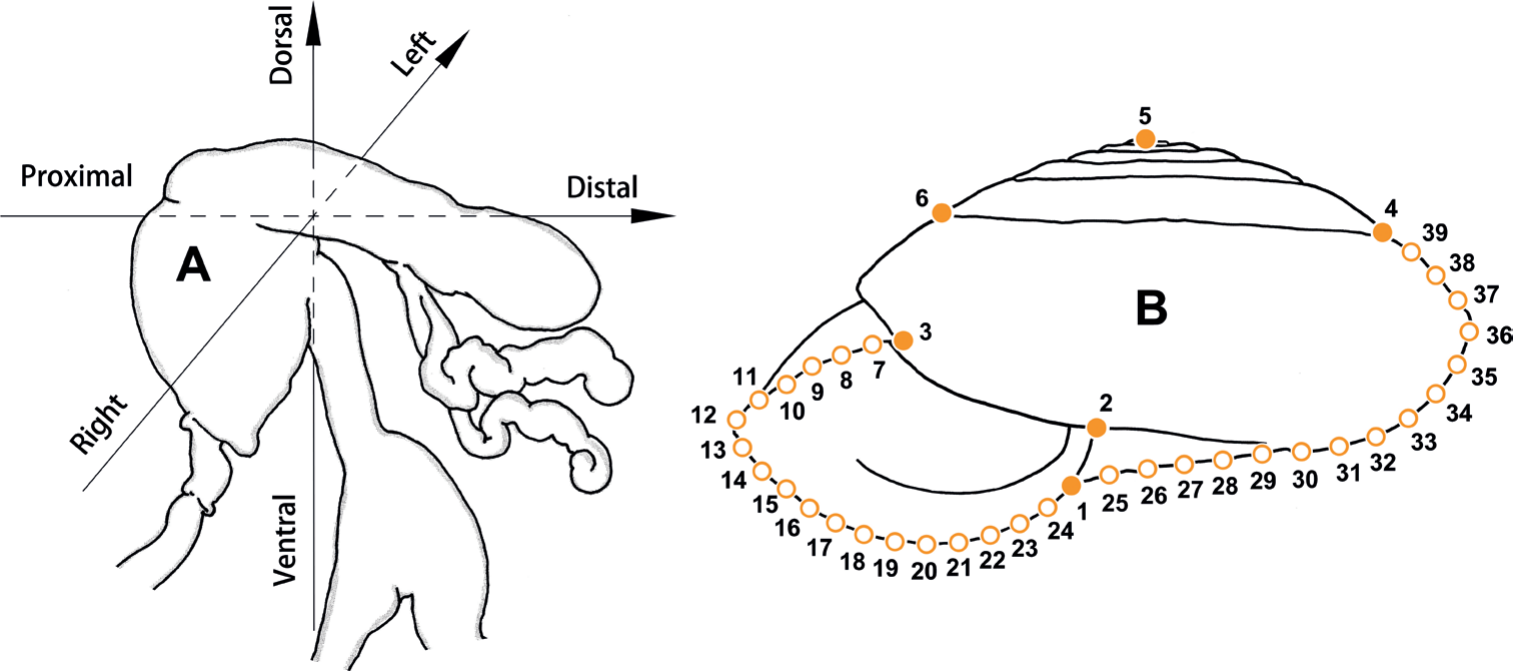
**A** directions used to describe dart sac apparatus **B** design of landmarks (solid dots) and semi-landmarks (empty dots).

Animals in most cases were relaxed by drowning in water before being transferred to 70% ethanol for fixation, which was replaced with ethanol of the same concentration after 3 days. To observe the animals with evaginated dart sac apparatus, before fixation the animal was drowned in water with 5% ethanol, 10% ethanol and 15% ethanol, in each solution for ~ 60 min. Photographs for illustrations (by MW) and geometric morphometric analyses (by WS) were taken using a Canon camera. The shells were measured with digital vernier calipers to the nearest 0.1 mm. Whorls were counted following Kerney and Cameron (1979) to the nearest 0.125 (= ^1^/_8_) whorls. Shells were observed under a scanning electron microscope Sigma 500. The penis was dissected at the opposite side of penial retractor insertion. The dart sac was dissected by cutting the dorsal part of the dart sac (see Fig. 1A) along the line from the atrium to the dart sac chamber. Directions used in the general descriptions of genitalia: proximal = towards the genital atrium; distal = away from the genital atrium. For directions used in the description of dart sac apparatus, refer to Fig. 1. Illustrations of genitalia were drawn based on actual photos (by MW). The corresponding Chinese name for person, new species, or locality is present only once in square bracket when necessary.

### ﻿Map preparation

**Figure 2.**
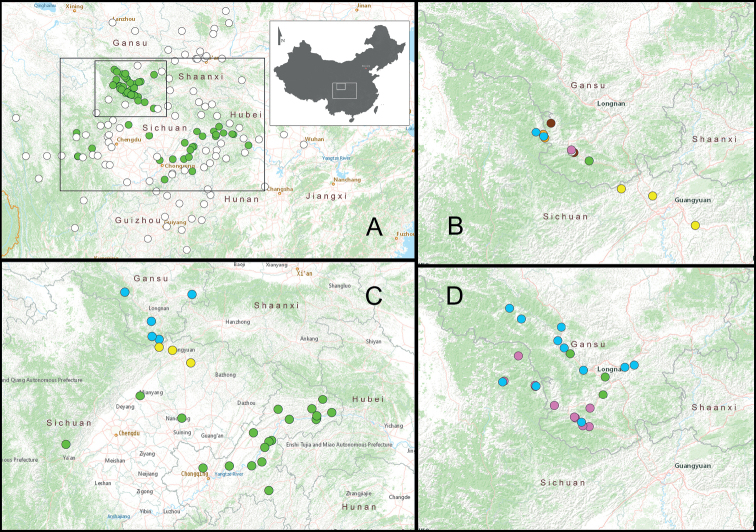
**A** distribution map of all known species of *Laeocathaica* Möllendorff, 1899 except *L.anceyi* (Möllendorff in Ancey, 1889), *L.hisanoi* Páll-Gergely, 2022, and *L.leucorhaphe* Möllendorff, 1899 whose precise localities are not known; white dots: the localities where no *Laeocathaica* species was ever found **B** distribution of the new species described in this paper, yellow dots: *L.qingchuanensis* Wu, sp. nov., green dot: *L.zhengpingliui* Wu, sp. nov., brown dots: *L.parapolytyla* Wu, sp. nov., white dot: *L.qiminglii* Wu, sp. nov., pink dot: *L.cheni* Wu, sp. nov., blue dots: *L.qishilii* Wu, sp. nov., orange dots: *L.nordsiecki* Wu, sp. nov. **C** distribution of *L.carinifera* (H. Adams, 1870) (green dots), *L.qingchuanensis* Wu, sp. nov. (yellow dots), and *L.stenochone* Möllendorff, 1899 (blue dots) **D** distribution of *L.amdoana* Möllendorff, 1899 (green dots), *L.distinguenda* Möllendorff, 1899 (pink dots), and *L.tropidorhaphe* Möllendorff, 1899 (blue dots) **C** equivalent to the larger box in (**A**), **B, D** equivalent to the smaller box in (**A**).

Distribution maps were created using ArcGIS Desktop 10.8 under GCS_WGS_1984 coordinate system. In addition, mapping was carried out to map both the distribution limit and the distribution pattern of *Laeocathaica* species (Fig. 2A). The nearby places where *Laeocathaica* was not found, represented by the counties/cities where the field work was carried out at one or more sites, are as follows: **Gansu Province**: Lixian [礼县], Qingyang [庆阳], Huanxian [环县], Huining [会宁县], Lanzhou [兰州]; **Guizhou Province**: Libo [荔波县], Jiangkou [江口县], Shibing [施秉县], Guiyang [贵阳市], Zhenning [镇宁县], Xishui [习水县], Meitan [湄潭县], Majiang [麻江县], Xifeng [息烽], Tongzi [桐梓县], Sansui [三穗县]; **Hubei Province**: Xianning [咸丰县], Wufeng [五峰县], Shennongjia in Hubei [湖北神农架], Zigui [秭归县], Xingshan [兴山县], Hefeng [鹤峰县], Enshi [恩施], Dangyang [当阳市], Jianshi [建始县], Wuchang [武昌], Jingshan [京山县], Lichuan [利川县], Xuanen [宣恩县]; **Hunan Province**: Taojiang [桃江县], Songtao [松桃苗族自治县], Xiushan [秀山县]; **Shaanxi Province**: Zhenping[镇坪县], Fengxian [凤县], Liquan [礼泉县], Luochuan [洛川县], Pingli [平利县], Yichuan [宜川县], Ziyang [紫阳县], Yanchang [延长县], Chunhua [淳化县], Huaxian [华县], Jingyang [泾阳县], Sanyuan [三原县], Linyou [麟游县], Shangnan [商南县], Baoji [宝鸡县], Liquan [礼泉县], Hanzhong [汉中市], Shiquan [石泉县], Huangling [黄陵县], Liuba [留坝县], Longxian [陇县], Xiba [西坝县], Langao [岚皋县]; **Sichuan Province**: Maoxian [茂县], Wenchuan [汶川县], Emei [峨眉], Luding [泸定县], Qingchengshan [青城山], Pingwu [平武县], Tianquan [天全县], Chongqing [崇庆县], Xicang [西昌], Mianzhu [绵竹县], Xinjin [新津], Yibin [宜宾], Leshan [乐山], Danba [丹巴县], Yaan [雅安市], Xiangcheng [乡城], Yajiang [雅江市], Batang [巴塘], Litang [理塘县], Dege [德格县], Dujiangyan [都江堰市], Panzhihua [攀枝花市], Songpan [松潘县], Heishui [黑水县], Hejiang [合江县], Lixian [理县], Wangcang [旺苍县], Nanjiang [南江县], Tongjiang [通江县], Xiaojin [小金县]; **Chongqing**: Wulong [武隆县], Chengkou [城口县], Hechuan [合川], Qianjiang [黔江县], Nanchuan [南川县]. It should be noted that the map (Fig. 2A) is far from complete, as any survey carried out in such a vast area could normally have provided very limited information about its malaco-fauna, not to mention that these places comprise 91 counties/cities of seven provinces, covering an area of ~ 1,080,000 km^2^ (calculated from Fig. 2A).

### ﻿Geometric morphometric analyses

Geometric morphometric methods (GMM) were used to investigate the relationship between some species that exhibits conchological similarities. The morphological variation analyses of the shells were performed in the tps series software including tpsUtil32 (Rohlf 2004), tpsDig32 (Rohlf 2005), and MorphoJ (Klingenberg 2011) using geometric morphometric methods based on the landmarks and semi-landmarks on the profile of the aperture-viewed shell (Schilthuizen et al. 2012; Wu et al. 2019a). The landmarks and semi-landmarks treated equally in the analyses were designed as follows: LM_1_, the crossing of peristome and right profile of body whorl; LM_2_, the columella insertion; LM_3_, the left insertion of peristome onto body whorl; LM_4_ and LM_6_, the right and left terminal points on last suture, respectively; LM_5_, apex of shell; LM_7–24_, eighteen semi-landmarks on the peristome between LM_3_ and LM_1_ by length; LM_25–39_, fifteen semi-landmarks on the right profile between LM_1_ and LM_4_ by length (Fig. 1B).

The material used in the GMM analyses were intact mature shells and the high-quality published photos, including *Laeocathaicaamdoana* Möllendorff, 1899: HBUMM05639 (2 specimens; the information of each collection lot is showed under each species in the section Systematics), -05640 (3), -05646 (5), -08147 (6), -08432 (3) and SMF 8952 (1); *Laeocathaicadistinguenda* Möllendorff, 1899: HBUMM05417 (5), -05434 (1), -05436 (1), -05473 (10), -05554 (4), -05565a (3), -05571 (3), -05576b (1), -06536 (3), -06556 (3), -06579 (2), -00674 (8), -08429 (2), CZG202008-w10 (4), CZG202008-w5 (5), SMF 8959 (1) and SMF 95024 (1); *L.tropidorhaphe* Möllendorff, 1899: HBUMM00450 (3), -00486 (2), -00508 (5), -00526 (1), -00527 (2), -05600 (8), -05621 (4), -05664 (6), -05692 (7), -05719 (4), -06621 (3), -08425 (7), CZG202008-w1 (2), SMF 9074 (1), SMF 9075 (1), SMF 9077 (1) and fig. 7D in Páll-Gergely et al. (2022) (paratype of *Laeocathaicadangchangensis* Chen & Zhang, 2004; 1); *L.carinifera* (H. Adams, 1870): HBUMM00006 (1), -01573 (5), -00228 (2), -04064 (5), -04162 (7), -04217 (8), -04219 (5), -04235 (5), -08443 (4), NHMUK V. W. McAndrew coll. Acc. No. 1563 (1), SMF 24265 (1), SMF 95116 (1) and fig. 12A in Páll-Gergely et al. 2022 (syntype; 1); *L.qingchuanensis* Wu, sp. nov.: HBUMM01179a (5), -04264 (2), -08195 (5), -08196 (4) and -08200 (9); and *L.stenochone* Möllendorff, 1899: HBUMM05495 (5), -05764a (1), -05767 (2), -05772 (4), -08431 (3), -08433 (1), SMF 8951 (1), SMF 9071 (1) and fig. 12D in Páll-Gergely et al. 2022 (lectotype; 1).

### ﻿Molecular studies and phylogenetic analyses

Whole genomic DNA was extracted from a piece of the foot muscle using the Animal Genome Quick Extraction Kit (B518221, Sangon Biotech). All PCR reactions were performed in 50 μL volumes containing 5 μL 10× reaction buffer containing 25 mm MgCl_2_, 10 mm of each dNTP, 1 μL of Taq polymerase (5 U/μL), 1 μL of 10–100 ng template DNA and ddH_2_O. Thermal cycling was performed on an Eastwin ETC811. PCR amplicons were inspected on a 1% agarose gel for quality and fragment size, then were sequenced on an automated sequencer. Primers used for partial fragment of 16S were 16Sar-L (5’-CGCCTGTTTATCAAAAACAT-3’) /16Sbr-H (5’-CCGGTCTGAACTCAGATCACGT-3’) (Palumbi et al. 2002). The conditions for thermal cycling: an initial denaturing step at 94 °C for 2 min, 35 cycles of denaturing at 94 °C for 30 s, annealing at 58 °C for 30 s and extending at 72 °C for 30 s, and final extending step of 72 °C for 10 min. A partial fragment of the ITS2 gene was amplified using the primer pair LSU-1 (5’-CTAGCTGCGAGAATTAATGTGA-3’)/ LSU-3 (5’-ACTTTCCCTCACGGTACTTG-3’) (Wade and Mordan 2000). The conditions for thermal cycling: 2 min at 94 °C for pre-denaturing, 35 circles of 30 sec at 94 °C, 30 sec at 58 °C and 60 sec at 72 °C.

Chromatographs and sequences were first studied and compiled in sequencer 4.5. DNA sequences of both genes were aligned by T-Coffee with standard parameters (Di Tommaso et al. 2011). Then the badly aligned parts (marked green and purple in T-Coffee, indicating inferior alignment) were deleted. For the subsequent analyses a concatenated matrix of 91 (incl. outgroup, table 1) × 1068 bp (incl. gaps) was used. The model selection was made using “Models” in MEGA 7.0.26 (Kumar et al. 2015). The dataset was analyzed by Bayesian Inference (BI) in MrBayes 3.2.4 (Ronquist et al. 2012). While the dataset was analyzed using the Maximum Likelihood Analysis in raxmlGUI 2.0 beta (Edler et al. 2019), applying the GTRGAMMA+I model and reps 1,000. Three independent runs were performed in Bayesian Inference Analysis, each of which was performed for 1,000,000 generations and sampled every 1,000 generations, where the first 25% samples were discarded as burn-in. The convergence of the Markov Chain Monte Carlo simulations was investigated with tracer v. 1.7 (Rambaut et al. 2018) to confirm that all ESS values exceeded 200. *Helixpomatia* Linnaeus, 1758 (Table 3) was used as an outgroup for rooting phylogenetic trees. New DNA sequences were deposited in the NCBI GenBank under the accession numbers ON261686–ON261865 (Table 3).

### ﻿Supraspecific classification

The generic classification of species adopted in this study mainly follows MolluscaBase (2021a–p; 2022a–c) (Table 3).

### ﻿Abbreviations

**AS** accessory sac;

**AO** opening of accessory sac leading to dart chamber;

**a.s.l.** above sea level;

**At** atrium;

**BC** bursa copulatrix;

**BCD** bursa copulatrix duct;

**DS** dart sac;

**Dt** love dart;

**DtC** a chamber containing love dart;

**DVM** a membranous (or muscular) sac surrounding proximal dart sac and/or distal region of vagina near atrium;

**Ep** epiphallus;

**fma** fully mature animal(s) (i.e., shell(s) and soft parts);

**fms** fully mature empty shell(s);

**FO** free oviduct;

**MG** mucous glands;

**juv** juvenile;

**P** penis;

**PAS** proximal accessory sac, a blind sac on proximal dart sac (= DVM);

**PO** opening of proximal accessory sac leading to dart sac chamber or dart chamber. In illustrations an PO is indicated by an asterisk or a red dot;

**PR** penial retractor muscle;

**PS** penial sheath;

**Va** vagina;

**VD** vas deferens.

### ﻿Depositories

**CZG** Personal collection of Chen, Zhong-Guang [陈重光], China;

**HBUMM** Mollusk collection of Museum of Hebei University (Baoding, China);

**NHMUK**Natural History Museum (London, UK);

**SMF**Senckenberg Forschungsinstitut und Naturmuseum (Frankfurt am Main, Germany);

**ZIN RAS**Zoological Institute, Russian Academy of Sciences (St. Petersburg, Russia).

## ﻿Results

### ﻿Comparative examination of the terminal genital organs in *Laeocathaica*

The most complicated structure in the genital system of dart-sac-bearing camaenid species (here, we use “dart-sac-bearing camaenids” to replace the former subfamily Bradybaeninae), if present, is the dart sac apparatus, a sack-like structure on the vagina. The basic structure of a dart sac apparatus consists of a dart sac, the muscular blind tube that contains and can secrete the love dart, the penis insertion/opening and the vagina insertion/opening. The ventral side of the dart sac usually has an accessory sac that is distally connected by mucous glands and opens into the chamber containing the love dart or opening into the dart sac chamber. The mucous glands consist of several mucous gland tubes that are simply branched (i.e., only one tube or bifurcate tube) or complicatedly branched and covered, packaged and connected by a sheet of loose connective tissue that binds the mucous glands more or less tightly to the vagina (Fig. 4B). The nerve fibers that connect the mucous gland tubes (e.g., Wu 2019: fig. 5C), together with the proximal nerve fibers that connect the dart sac (incl. accessory sac), fuse into a main nerve fiber that is attached to the surface of the proximal oviduct before it enters the body wall of the animal. Bilateral symmetry (for directions see Fig. 1A) of a dart sac apparatus consisting of the above components has been observed in many genera of Bradybaeninae (Wu 2004). However, the position of the membranous sac surrounding the partial dart sac and/or distal region of the vagina proximal to the atrium (DVM) makes the dart sac asymmetric in some genera, e.g., in *Laeocathaica*. The DVM in dart-sac-bearing camaenid snails, has been ignored in many studies (e.g., those works before Wu and Guo 2003), or not particularly emphasized (e.g., in *Stilpnodiscusentochilus* Möllendorff, 1899, Wu (2004: fig. 16E): the sac around the space between V2 and V3), or treated by some authors as DVM (in *Trichobradybaena* Wu & Guo, 2003; in many species in Wu 2004; Wu 2019: table 1 for incomplete taxa, see below), or with alternative nomenclature referring to structures which are close in position and/or shape and blind or open into the dart sac chamber (i.e., “finger-shaped structure” in *Stilpnodiscusmoellendorffi* Wu, 2001; “bladder” in *Laeocathaicapolytyla* in Schileyko (2004); “bridge-like structure” in *Ponsadenia* spp. in Wu and Guo (2006); “proximal accessory sac”, in *Traumatophoratriscalpta* (Martens, 1875) in Wu (2019) and in *Pseudiberus* spp. where both blind ones or those open into the dart sac chamber were observed in Zhang et al. (2021)). The opening of the DVM was not recorded prior to observation in *T.triscalpta* (Wu 2019).

The so-called DVM structure cannot be distinguished from the proximal accessory sac in position, external morphology or internal structure. The fact that the conjunction of DVM structure and the proximal accessory sac was not observed in any Chinese bradybaenine genus (Wu 2019: table 1) suggests that they are originally the same organ. Consequently, in this work, the proximal accessory sac refers to “a membranous (or muscular) sac that partially surrounds the dart sac and/or distal region of the vagina proximal to the atrium” that does not differ from the DVM.

The proximal accessory sac, whose opening leads to the outside of the body (if is not blind) (Fig. 4), has been speculated to be the place where the fluid secreted by the mucous glands is stored (Zhang et al. 2021). Connecting with the chambers inside the dart sac apparatus, the PLS structure (e.g., in Aegista (Aegista) accrescens (Heude, 1882), Wu (2004: fig. 13C); Aegista (Plectotropis) gerlachi (E. Martens, 1881), Wu (2004: fig. 14B–D); in *Metodontiahouaiensishouaiensis* (Crosse, 1882), Wu and Prozorova (2006: fig. 3C), has yet to be proven that it is a modified PAS. When the dart sac apparatus in *Laeocathaica* is evaginated outwards to the body wall prior to dart shooting and epiphallus protrusion, an external sac is formed exposing the penial, vaginal and dart sac orifices and the proximal accessory sac (Fig. 4). Functionally, the proximal accessory sac forms a “cushion” (Fig. 4, arrowed) filled with the fluid secreted by the mucous glands during the pre-copulatory behavior. The shape, size, and number of openings of the proximal accessory sac are believed to be related to the compatibility of courtship behavior, which affects the efficiency of copulation.

**Figure 3.**
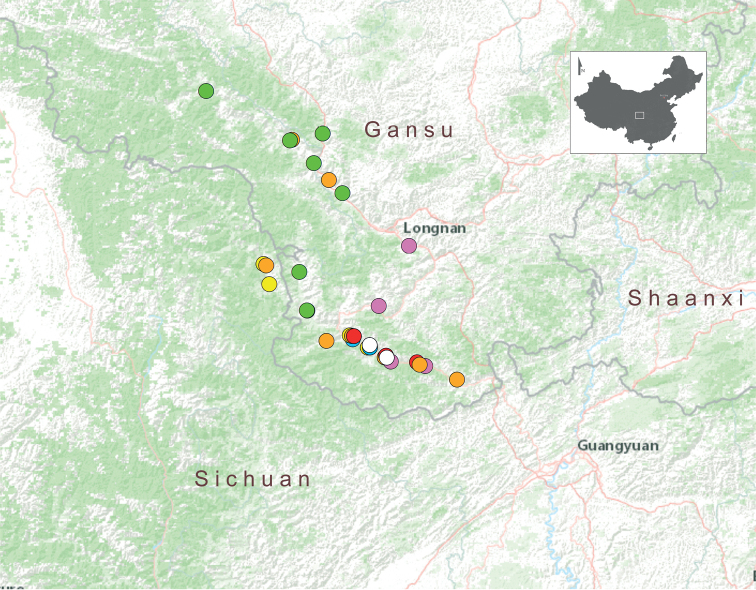
Distribution map of *Laeocathaicadityla* Möllendorff, 1899 (green dots), *L.odophora* Möllendorff, 1899 (pink dots), *L.pewzowi* Möllendorff, 1899 (white dots), *L.phaeomphala* Möllendorff, 1899 (blue dots), *L.polytyla* Möllendorff, 1899 (yellow dots), *L.potanini* Möllendorff, 1899 (red dots), and *L.prionotropis* Möllendorff, 1899 (orange dots). Range of the map is defined by the rectangle in the thumbnail.

**Figure 4.**
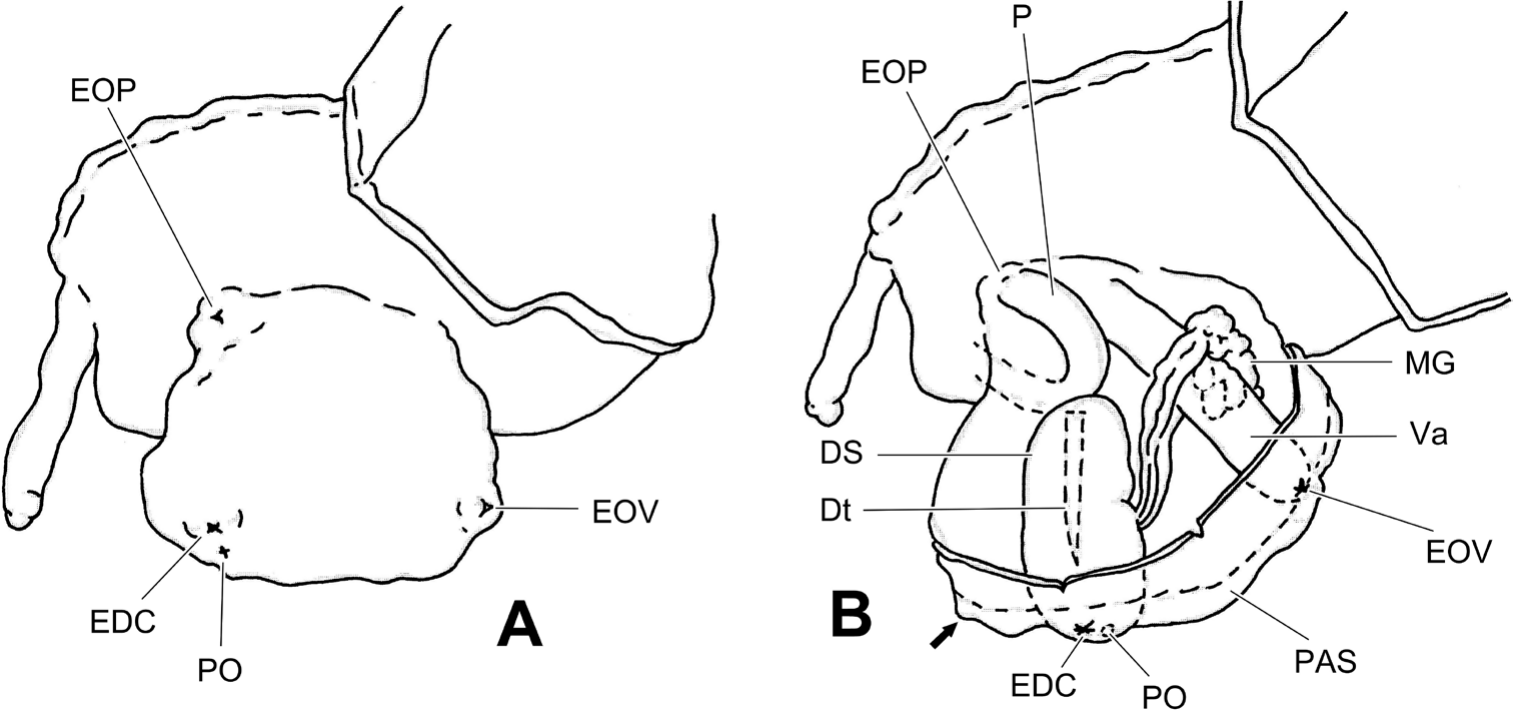
Terminal genitalia in the evaginated pouch in *Laeocathaicaqishilii* Wu, sp. nov. (HBUMM08298-spec.8, paratype), showing relative position of dart sac apparatus, vagina, and penis **A** evaginated pouch of terminal genitalia **B** the exposed pouch. Arrow indicates the position of the proximal accessory sac. Abbreviations: DS – dart sac; Dt – love dart; EDC – entrance of dart chamber; EOV – external opening of vagina; EOP – external opening of penis; MG – mucous glands; P – penis; PAS – proximal accessory sac; PO – opening of proximal accessory sac leading to dart sac chamber or dart chamber; Va – vagina.

In *Laeocathaica* it is most commonly observed that two proximal accessory sacs, the left and the right, each with a pore leading to the dart chamber or dart sac chamber, are dorsally separated and ventrally fused. For *Laeocathaicaphaeomphala* Möllendorff, 1899, *L.polytyla* Möllendorff, 1899, and *L.tropidorhaphe*, the dart sac has no proximal accessory sac (Figs 23, 26, 34). The only species that has one proximal accessory sac is *Laeocathaicadolani* (Pilsbry, 1934) (Fig. 7). Two of the three species without proximal accessory sac have the elongated vagina part between atrium and dart sac (Figs 23A, 34A; Table 1). However, the third species *Laeocathaicapolytyla* has neither a proximal accessory sac nor the elongated vagina part (Fig. 26). We can hardly guess how the love dart is to be exposed outside body through the long distance of the elongated vagina part, based on our knowledge of the normal situation of dart shooting in the genus, as showed in Fig. 4. The presence of two proximal accessory sacs in *Laeocathaicaamdoana*, which has the relatively smallest proximal accessory sac in the genus (Fig. 6B, C), makes *L.amdoana* a species that may has a transitional state among the species that have large and ventrally fused proximal accessory sacs (e.g., in *L.carinalis* Chen & Zhang, 2004, Fig. 8), have large but ventrally separated proximal accessory sacs (e.g., in *L.zhengpingliui* Wu, sp. nov., Fig. 38) and have no proximal accessory sac (e.g., in *L.phaeomphala*, Fig. 23).

**Table 1.** Some character states of the terminal genitalia in *Laeocathaica* Möllendorff, 1899.

Characters\Species	Vagina elongated above dart sac (+) or not (–)	Number of mucous glands	Branching of mucous gland duct complicated (+) or simple (–)	Blades of apical dart	Number of PAS	PAS: Symmetrical (+) or asymmetrical (–)	Number of PAS opening(s)	PAS: ventrally touching (+) or not (–)
*L.amdoana* Möllendorff, 1899	+	8	+	2	2 (tiny)	+	2	–
*L.carinalis* Chen & Zhang, 2004	–	4	–	/	2	+	2	+
*L.carinifera* (H. Adams, 1870)	–	6–7	+/–	/	2	–	2	+
*L.christinae* (H. Adams, 1870)	–	4–5	+	2	2	+	2	–
*L.distinguenda* Möllendorff, 1899	–	4–12	+/–	2	2	+	2	–
*L.dityla* Möllendorff, 1899	–	2–3	–	?	2	+	2	+
*L.dolani* (Pilsbry, 1934)	–	2–3	+	4	1	–	1	/
*L.filippina* (Heude, 1882)	–	5–7	+	?	2	+	2	+
*L.odophora* Möllendorff, 1899	–	5	–	2	2	–	2	+
*L.powzowi* Möllendorff, 1899	–	4	–	/	2	+	2	+
*L.phaeomphala* Möllendorff, 1899	+	8	–	/	0	/	/	/
*L.polytyla* Möllendorff, 1899	–	5	–	2	0	/	/	/
*L.potanini* Möllendorff, 1899	–	2–3	+	4	2	+	2	+
*L.prionotropis* Möllendorff, 1899	–	4–8	–	4	2	+	2	+
*L.stenochone* Möllendorff, 1899	–	5	+	2	2	+	2	+
*L.tropidorhaphe* Möllendorff, 1899	+	7–10	+	4	0	/	/	/
*L.parapolytyla* Wu, sp. nov.	–	4–5	–	/	0	/	/	/
*L.qishilii* Wu, sp. nov.	–	2	+	2	2	+	2	+
*L.qiminglii* Wu, sp. nov.	/	/	/	/	/	/	/	/
*L.zhengpingliui* Wu, sp. nov.	+(somewhat)	6	–	0	2	+	2	–
*L.cheni* Wu, sp. nov.	–	4	–	2	2	+	2	+
*L.qingchuanensis* Wu, sp. nov.	+	6–8	–	/	2	+	2	+

Unlike the structurally closed proximal accessory sac observed only in *Pseudiberustectumsinensepingi* Zhang & Wu, 2021, the proximal accessory sac observed in all *Laeocathaica* species has an opening/pore leading to the dart chamber (for most species) or dart sac chamber (for *L.amdoana* only) instead of the proximal accessory sac opening leading only to dart sac chamber as observed in *Traumatophora* Ancey, 1887 (Wu 2019) and in *Pseudiberus* Ancey, 1887 (Zhang et al. 2021). In *Laeocathaica*, the proximal accessory sac pore is located at the distal end of the proximal accessory sac, opening into the proximal dart chamber, or at the central part of the proximal accessory sac, opening into the dart sac chamber. Accordingly, if the dart sac is evaginated during the mating of the snail, the pore is exposed to the outer environment or not deep in the dart chamber (Fig. 4).

The accessory sac in some *Laeocathaica* species is tiny in appearance and internally solid, with the mucous glands entering papilla (e.g., arrowed in Wu 2004: fig. 19B) missing (in *Laeocathaicaamdoana*, *L.dityla* Möllendorff, 1899, *L.distinguenda*, *L.polytyla*, *L.potanini* Möllendorff, 1899, *L.stenochone*, *L.qishilii* Wu, sp. nov., *L.cheni* Wu, sp. nov.; Figs 6B, 14D, 16C, 26C, 28B, 32B, 36C, 39C). Even in species with an internally empty accessory sac, the mucous glands entering papilla is absent (in *Laeocathaicadolani*, *L.filippina* (Heude, 1882), *L.odophora* Möllendorff, 1899, *L.phaeomphala*, *L.prionotropis* Möllendorff, 1899, *L.tropidorhaphe*; *L.zhengpingliui* Wu, sp. nov., and *L.qingchuanensis* Wu, sp. nov.; e.g., in Fig. 7B).

The penis of *Laeocathaica* is divided into three parts according to the structures that protrude on the inner wall. The proximal part consists of main/thicker pilasters. The central part is covered by cross-linked/network-like fine pilasters branched off from the main pilasters of the proximal part (e.g., in *Laeocathaicaprionotropis*, Fig. 43D), or covered by isolated tongue-like/diamond-like granules that erect on the penis wall (in *L.cheni* Wu, sp. nov., Fig. 44F, H). The distal part is the distal end of the penis near the epiphallic opening, where the proximal mini-pilasters aggregate and form several short and more or less thick pilasters. All these three parts are intraspecifically stable, but more or less divergent among species. In the proximal part of the penis, it is characteristic that one (in most cases, i.e., in *Laeocathaicaamdoana*, *L.carinalis*, *L.cheni* Wu, sp. nov., *L.distinguenda*, *L.dityla*, *L.dolani*, *L.odophora*, *L.polytyla*, *L.potanini* and *L.qishilii* Wu, sp. nov.) (Figs 41–44) or two (i.e., in *L.carinifera*, *L.prionotropis*, *L.tropidorhaphe*, *L.stenochone*, *L.parapolytyla* Wu, sp. nov., and *L.zhengpingliui* Wu, sp. nov.) (Figs 42–44) pairs of adjacent pilasters merge into one or two Y-shaped forks. In *Laeocathaicastenochone*, two pilasters form a Y-shaped fork (Fig. 43E, right arrow) and two other parallel pilasters merge into a rather thick pilaster (Fig. 43E, left arrow). Such a Y-shaped fork, formed by two adjacent pilasters, was not observed only in very few species (i.e., *Laeocathaicachristinae* (H. Adams, 1870), *L.phaeomphala*, and *L.qingchuanensis* Wu, sp. nov.) (Figs 42C, D, 44E).

### ﻿Systematics

#### ﻿Family Camaenidae Pilsbry, 1895

##### ﻿Genus Laeocathaica

Taxon classificationAnimaliaStylommatophoraCamaenidae

Möllendorff, 1899

A3BE6C9E-0F99-5169-8407-437356ABBE71

Laeocathaica Möllendorff, 1899: 86; Richardson 1983: 77; Schileyko 2004: 1686.

###### Type species.

Helix (Plectotropis) christinae H. Adams, 1870 (by original designation).

###### Description.

Shell sinistral, strongly depressed to broadly conic, moderately solid, of 5–10.5 almost flat whorls. Last whorl abruptly descending in front, angulated to strongly keeled; rarely rounded. Coloration consists of whitish, corneous, or chestnut background and mostly with one or two dark sub-peripheral bands; besides, usually there are several fulvous, diffuse radial streaks. Protoconch usually with fine radial threads and/or fine granules that each is formed by a low hump deposited in a shallow socket. Sculpture of teleoconch whorls varying from fine, silky radial striation to rather strong ribbing; on basal surface below keel or angle this sculpture becomes much weaker. Aperture rounded to peach-shaped, oblique, margins usually more or less reflexed. Within aperture a ring-like thickening present. Apertural teeth absent or with one tuberculiform basal tooth and sometimes with another one on palatal wall. During postembryogenesis several sets of teeth, different from those developed at adult stage in shape and/or number, present and remained to adult stage. Umbilicus moderate to very wide, ratio of umbilicus diameter to maximum diameter 0.21–0.50. Height 3.5–14 mm, maximum diameter 10.0–29.5 mm.

On left and right side of mantle edge, no leaf-shaped appendage present. Head wart between ommatophores present, weak, or developed. Jaw arcuate, with 3–8 more or less projecting ribs.

Slender vas deferens entering epiphallus at penial retractor muscle insertion. Penis generally clavate, rarely subcylindrical. Penis internally divided into three regions: the proximal part with narrow or thick longitudinal pilasters, among which one pair or two pairs of adjacent pilasters fuse into one Y-shaped fork or two Y-shaped forks (not in *Laeocathaicachristinae*, *L.phaeomphala*, and *L.qingchuanensis* Wu, sp. nov.). The median part, composed of fine pilasters that weave into network or covered by isolated tongue-like/diamond-like granules erecting on penial wall. The distal part, near epiphallic opening, with mini-pilasters crowded and forming several short and thick pilasters. Epiphallic papilla absent. Penial sheath always present, surrounding proximal penis. Dart sac always present. Accessory sac presents at ventral dart sac, internally solid or narrowly empty. Mucous glands 2–12, each simply or complicatedly branched, entering accessory sac separately before being united into a common duct inside wall of accessory sac. Proximal section of dart sac with 0–2 PAS that if present, each has a tiny opening leading to dart chamber. Vagina between atrium and dart sac elongated only in a few species. Bursa copulatrix duct subcylindrical throughout (modified from Schileyko 2004).

###### Distribution.

China: S Gansu, W Hubei, W Shaanxi, Chongqing, Sichuan.

###### Remarks.

*Laeocathaica* species are granulate on the protoconch, which is smooth in actual observation due to erosion or weathering. In the original description of *Laeocathaicafilippina*, Heude (1882) first mentioned the irregular white radiate stripes (1882), which were particularly noted by Möllendorff (1899) as “die stets vorhandenen Jugendlippen (the ever-present juvenile lips)” that joined the definition of *Laeocathaica*. Möllendorff (1899) also noticed in *Euhadrahaplozona* Möllendorff, 1899 and *Euhadraeris* Möllendorff, 1899 such “ever-present juvenile lips” exist. The juvenile lips, which have remained on the mature shell, may be frequent and weak such as those of *L.minwui* Páll-Gergely, 2022 (Fig. 11A) and many other species, or sparse and strong as those of *Laeocathaicadityla* (Fig. 13D) and *L.parapolytyla* Wu, sp. nov. (Fig. 25). Regarding the genital organs, the Y-shaped forks present on the proximal part of inner wall of the penis were observed exclusively in most anatomically studied *Laeocathaica* species among the Chinese dart-sac-bearing camaenids.

##### ﻿ Laeocathaica amdoana

Taxon classificationAnimaliaStylommatophoraCamaenidae

Möllendorff, 1899

ADC7D54B-847F-567B-9559-7DF8581B2718

Figs 2A, D , 5A, B , 6 , 41A, B , 46G, H , 50A , 51 , 52E ; Tables 1 , 3

Laeocathaica amdoana Möllendorff, 1899: 92, pl. 5, fig. 5; – Gude 1902: 6; – Yen 1939: 148, pl. 15, fig. 31; – Páll-Gergely et al. 2022: 38, fig. 3A, B.
Laeocathaica stenochone amdoana – Wiegmann 1900: 104, pl. 3, figs 91–93.
Laeocathaica (Laeocathaica) amdoana –Zilch 1968: 173; – Richardson 1983: 77.

###### Museum material.

ZIN RAS No. 2, 1 fma and 1 subadult, “*Aegistaamdoana* Möllendorff.”, Wen-Xian [文县], 1885-IX-8, coll. Potanin, det. Möllendorff. SMF 8952, lectotype, Ho-dshi-gou, Gansu, China. ex Potanin 853. Slg. O. v. Möllendorff. SMF 8953, paratype, Wen-hsien, SO-Gansu, China.

###### New material.

HBUMM05600, numerous fma and juvs; southern slope of Beishan [北山], Wudu [武都], Longnan [陇南], Gansu Province, limestone hill with sparse shrubs; 2006-X-02, coll. Liu, Jian-Min [刘建民], Zheng, Wei [郑伟]. HBUMM05640, numerous specimens, 2 fma dissected, Jiaogongzhen [角弓镇], Wudu, Gansu Province, (33.57°N, 104.64°E), broken limestone rocks, 2006-X-02, coll. Liu, J.-M. and Zheng, W. HBUMM05639, 4 fma and 1 juv, 1 fma dissected; Jiaogongzhen, Wudu, Gansu Province (33.57°N, 104.64°E), broken limestone rocks, 2006-X-02, coll. Liu, J.-M. and Zheng, W. HBUMM8147, 1 fma dissected: coll. data as HBUMM05640; DNA voucher HBUMM05626. HBUMM08147, 1 fma dissected, Wudu, Gansu Province, 927 m a.l.s., (33.346111°N, 105.044°E), adults on cliff and juvs on deadwood, 2017-VIII-05, coll. Shen, Xue-Fen [盛雪芬], etc.; DNA voucher HBUMM08147a. HBUMM08432, near Shichuanba [石川坝], Wenxian, Gansu Province, near 33.17534°N, 105.019362°E; 2019-X-12, coll. Li, Qi-Ming [李启明]; DNA voucher HBUMM08432a.

###### Distribution.

Gansu: Wudu, Wenxian (type locality).

###### Additional information of shell.

Very fine and slim granules (~ 20 µm long) are present on the protoconch. After the fourth whorl, including the umbilical region, irregularly arranged spiral grooves are present. On teleoconch whorls, the growth lines are low and indistinct.

###### General anatomy.

Eversible head wart lowly present. Jaw arcuate, with five projecting ribs.

###### Anatomy of genital organs.

Penial sheath moderately long, covering ~ 1/3 of penis. Penis slightly expanded distally. Inside penis, two penial internal pilasters fusing into Y-shaped fork at proximal ^1^/_4_, accompanied with 4–6 low pilasters. Distally inside penis, numerous fine pilasters merging into ~ 4 short but thick folds near opening of epiphallus. Vas deferens narrow throughout. Vagina between atrium and dart sac elongated, ~ 2 times longer than dart sac. Vagina between dart sac and insertion of bursa copulatrix duct short, ~ 3 mm in length. Dart sac ~ 1/3 length of penis. Love dart ~ 1.2 mm long, apically 2-bladed. Accessory sac small, internally solid, inserting into dart sac medially, opening to distal dart chamber. Mucous glands seven or eight, each complicatedly branched. Proximal accessory sacs two, not touching; each proximal accessory sac tiny, ~ 0.5 × 0.5 mm^2^ (HBUMM05640-spec.1) or smaller (HBUMM05640-spec.2) in size; each with an opening leading to dart sac chamber. Bursa copulatrix duct of even diameter. Bursa copulatrix ovate.

###### Remarks.

By shell sculpture, including that on the protoconch, this species (Fig. 46G, H) cannot be immediately distinguished from *Laeocathaicatropidorhaphe* (Fig. 46I, J) and *L.distinguenda* (Fig. 46A, B). It seems that *L.tropidorhaphe* forms a continuous variation of shell shape and the conchological delimitation among these three species that are geographically coexistent is not so distinct (Páll-Gergely et al. 2022). According to our observation, the key morphological features to practically distinguish the three species are:

**Figure 5.**
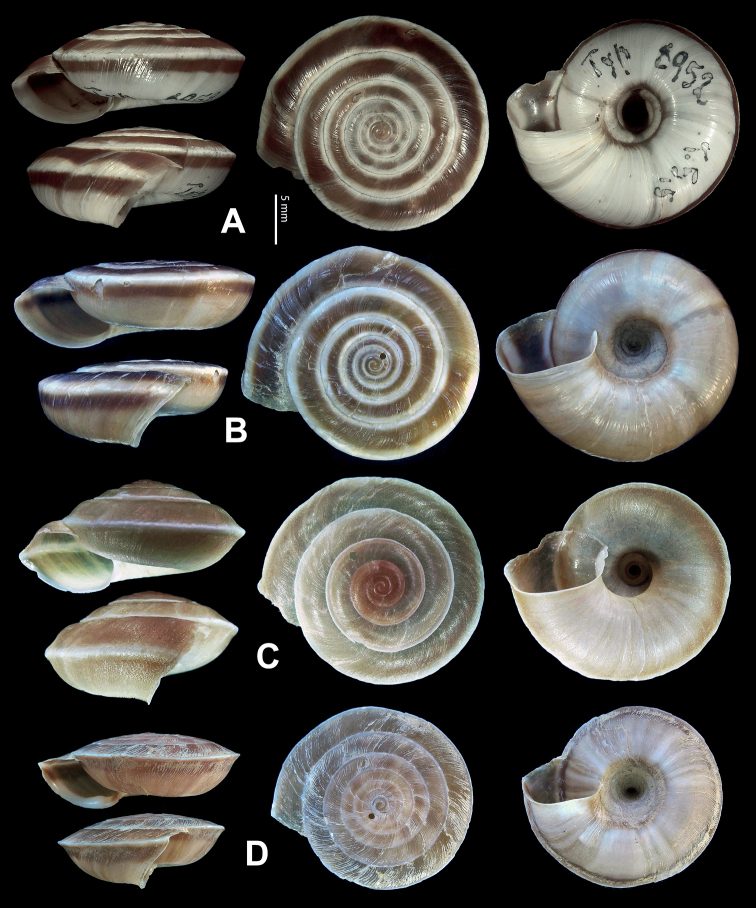
**A, B***Laeocathaicaamdoana* Möllendorff, 1899 **A**SMF 8952, lectotype **B** HBUMM05640-spec.1 **C***L.dolani* (Pilsbry, 1934), HBUMM00069-spec.1 **D***L.carinalis* Chen & Zhang, 2004, HBUMM08400-spec.1.

1. brownish/chestnut coloration. In *Laeocathaicaamdoana* such coloration is usually (!) obviously darker and in *L.distinguenda* the coloration is usually very pale (in our Fig. 15C, it is a relatively darker shell). However, in HBUMM05479 (dissected, shell not pictured) the shell is in very pale dirty yellow except peripherally whitish.
2. peripheral angulation. The level of angulation, in order is *Laeocathaicatropidorhaphe*> (>: stronger than; >>: much stronger than) *L.amdoana*>> *L.distinguenda*, which is also indicated by the geometric morphometric analyses: compared to *L.amdoana* (Fig. 5A, B; average shape ‘b’ in Fig. 50A) and *L.tropidorhaphe* (Fig. 33; average shape ‘c’ in Fig. 50A), the shell of *L.distinguenda* appears to be much broader in aperture and the periphery is very bluntly round (Möllendorff 1899).
3. the structure of umbilicus. In both *Laeocathaicaamdoana* and *L.tropidorhaphe*, the umbilicus is funnel-shaped and through which every whorl is visible. However, in *Laeocathaicadistinguenda*, the penultimate whorl is much projecting and makes a suddenly enlarged umbilicus (Möllendorff 1899; our own observation, e.g., to compare Fig. 15 with Figs 5A, B, 33). *Laeocathaicaamdoana*, *L.distinguenda*, and *L.tropidorhaphe* are clearly distinguishable based on genital features. *Laeocathaicaamdoana* and *L.tropidorhaphe*, both of which have a long vagina between dart sac apparatus and atrium that was not seen in *L.distinguenda*, can be distinguished by the presence of proximal accessory sac in the former species. *Laeocathaicatropidorhaphe* belongs to the three species in *Laeocathaica* that have no proximal accessory sac. In *Laeocathaicaamdoana* there are two tiny proximal accessory sacs, and each has an opening leading to the dart sac chamber, which means the proximal accessory sacs could be functional compared to the blind one (i.e., without opening) observed in *Pseudiberustectumsinensepingi* (Zhang et al. 2021). In addition, in *Laeocathaicaamdoana* the dart is apically 2-bladed rather than 4-bladed in *L.tropidorhaphe*.

The phylogeny based on ITS2 and 16S suggests that *Laeocathaicaamdoana* is sister to *L.distinguenda* and *L.tropidorhaphe* (Fig. 51). In Chen and Zhang (2004: 316, fig. 303), the species identified as *Laeocathaicaamdoana* is dubious and looks like a *L.distinguenda*. For more comments, see *Laeocathaicatropidorhaphe*.

**Figure 6.**
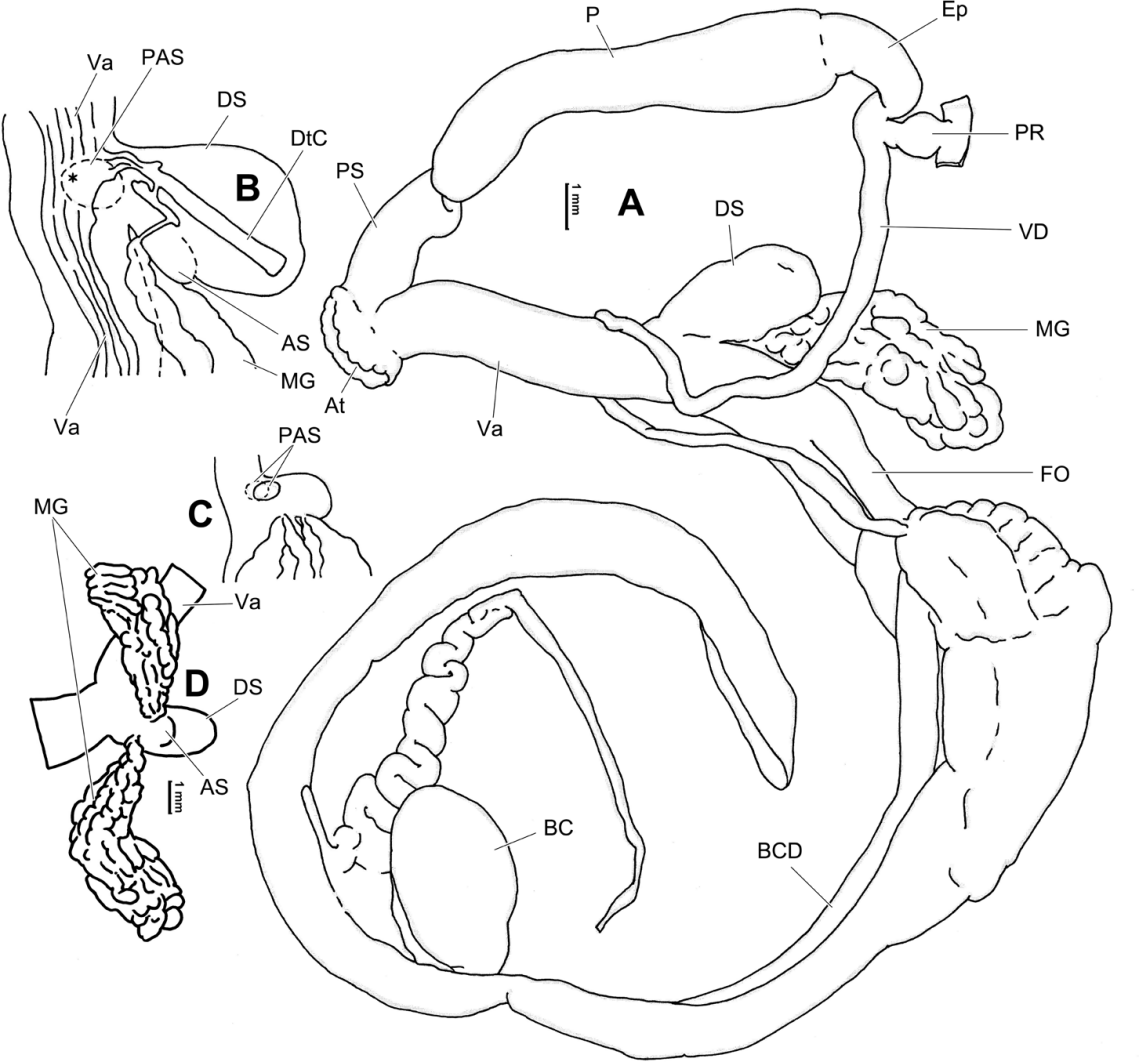
Genital anatomy of *Laeocathaicaamdoana* Möllendorff, 1899, HBUMM05640-spec.1 **A** general view **B** left part of dart sac apparatus **C** right view of dart sac apparatus, showing position of proximal accessory sacs **D** ventral view of dart sac apparatus. Abbreviations: AS – accessory sac; At – atrium; BC – bursa copulatrix; BCD – bursa copulatrix duct; DS – dart sac; DtC – a chamber containing love dart; Ep – epiphallus; FO – free oviduct; MG – mucous glands; P – penis; PAS – proximal accessory sac; PR – penial retractor muscle; PS – penial sheath; Va – vagina; VD – vas deferens. Asterisk * indicates the opening of proximal accessory sac.

**Figure 7.**
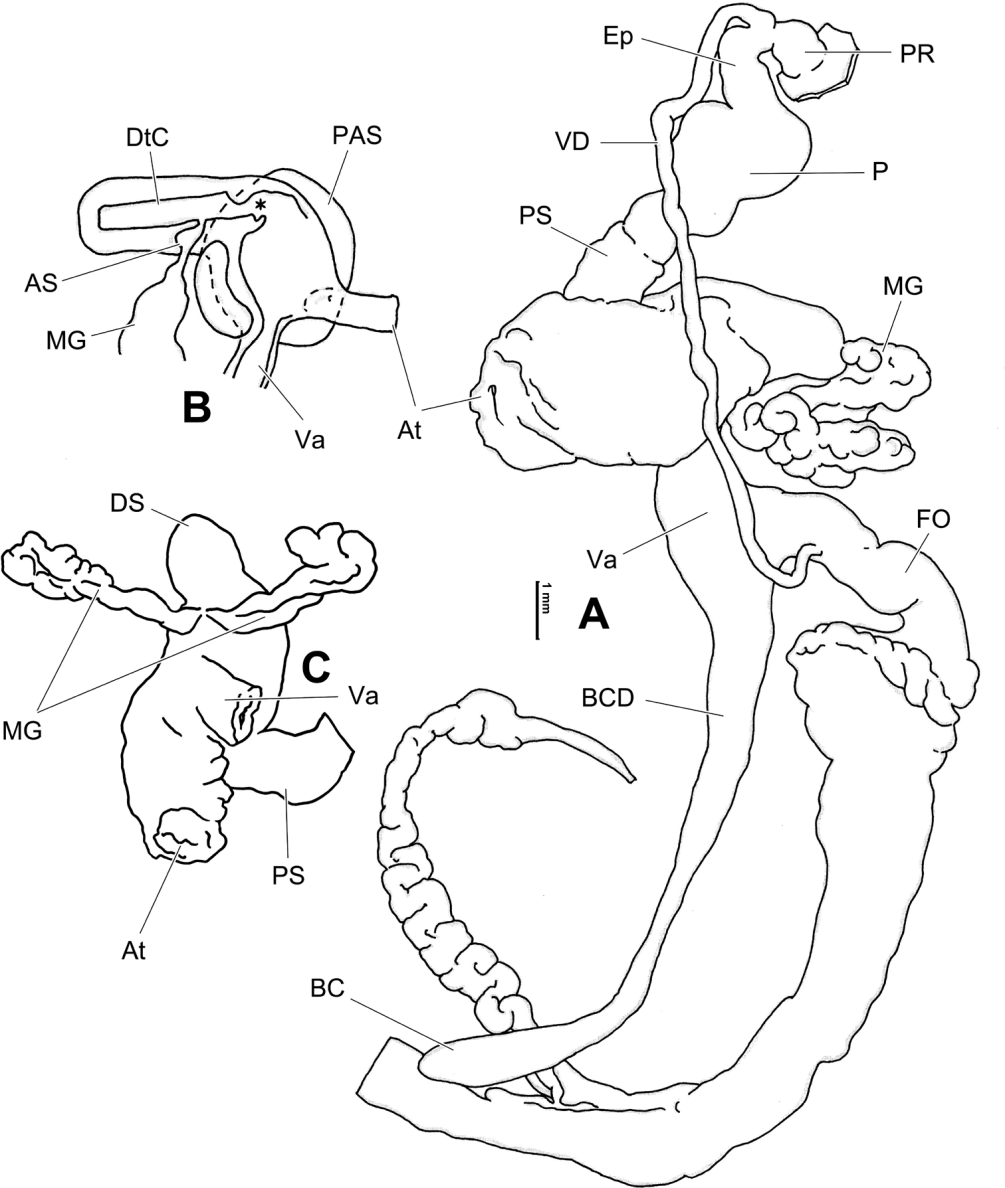
Genital anatomy of *Laeocathaicadolani* (Pilsbry, 1934) **A** general view, HBUMM08439-spec.1 **B** left view of dart sac apparatus, HBUMM08439-spec.1 **C** ventral view of dart sac apparatus, HBUMM00069-spec.1. Abbreviations: AS – accessory sac; At – atrium; BC – bursa copulatrix; BCD – bursa copulatrix duct; DS – dart sac; DtC – a chamber containing love dart; Ep – epiphallus; FO – free oviduct; MG – mucous glands; P – penis; PAS – proximal accessory sac; PR – penial retractor muscle; PS – penial sheath; Va – vagina; VD – vas deferens. Asterisk * indicates the opening of proximal accessory sac.

##### ﻿ Laeocathaica anceyi

Taxon classificationAnimaliaStylommatophoraCamaenidae

(Möllendorff in Ancey, 1889)

3F56C658-E874-5B52-BA01-694D95CB3B8E

Helix anceyi Möllendorff in Ancey, 1889: 205.
Laeocathaica anceyi – Pilsbry 1892: 215; – Gude 1902: 8.
Laeocathaica (Laeocathaica) anceyi – Richardson 1983: 77.

###### Examined specimens.

None.

###### Distribution.

Sichuan.

##### ﻿ Laeocathaica carinalis

Taxon classificationAnimaliaStylommatophoraCamaenidae

Chen & Zhang, 2004

05D5831B-947D-580B-8ABB-168C18BF373A

**Figure 8.**
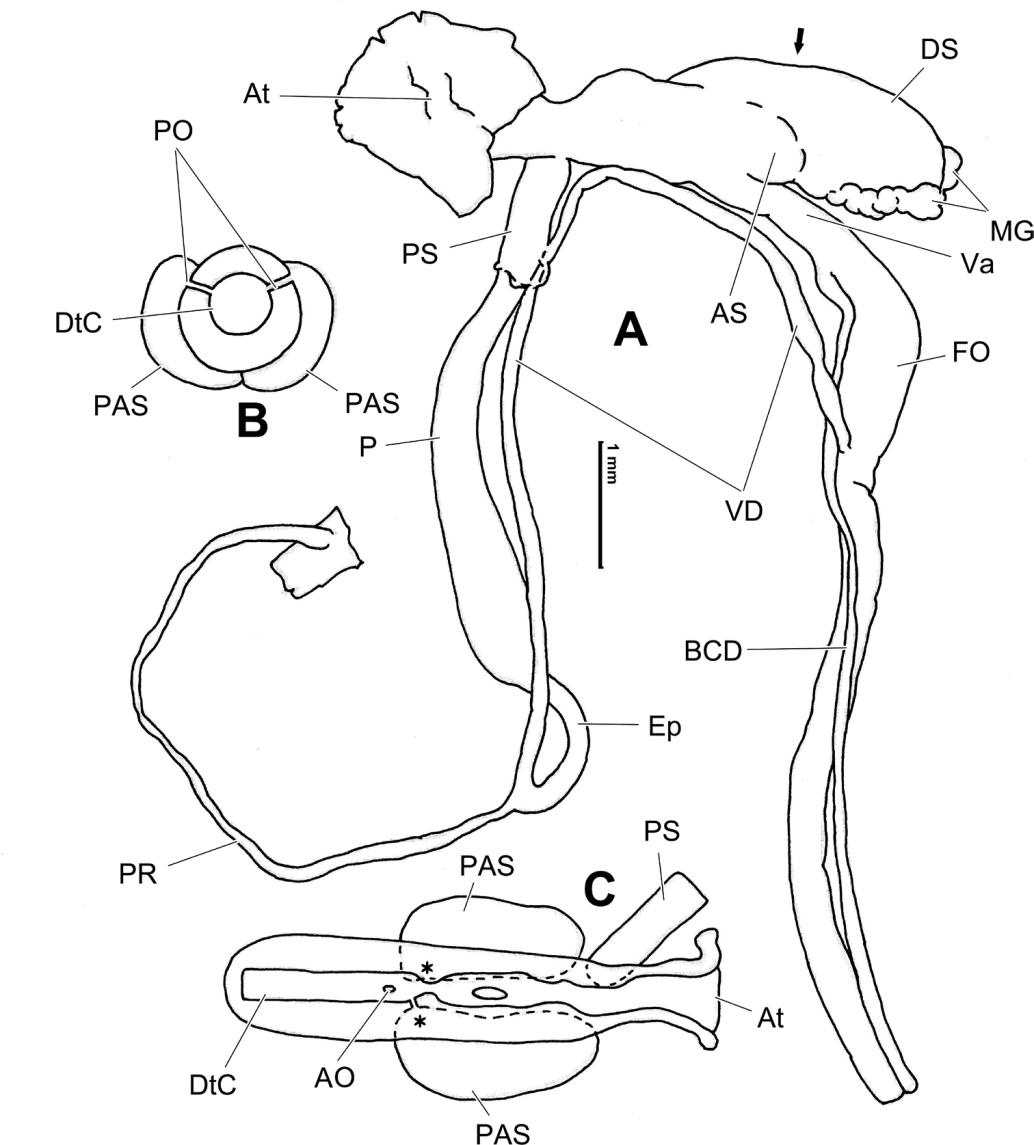
Genital anatomy of *Laeocathaicacarinalis* Chen & Zhang, 2004, HBUMM08300-spec.1 **A** general view **B** cross-section of dart sac at the position arrowed in (**A**) **C** apical view of dart sac apparatus. Abbreviations: AS – accessory sac; At – atrium; BCD – bursa copulatrix duct; DS – dart sac; DtC – a chamber containing love dart; Ep – epiphallus; FO – free oviduct; MG – mucous glands; P – penis; PAS – proximal accessory sac; PO – opening of proximal accessory sac leading to dart chamber; PR – penial retractor muscle; PS – penial sheath; Va – vagina; VD – vas deferens. Asterisk * indicates the opening of proximal accessory sac.

Figs 2A , 5D , 8 , 41F , 47I, J ; Table 1

Laeocathaica carinalis Chen & Zhang, 2004: 341, in Chinese, with erroneous text figure (fig. 334); – Páll-Gergely et al. 2022: 46, figs 9, 10.

###### New specimens.

HBUMM08300, 3 fma, all dissected, ?Wenxian, Gansu Province, 2019-IV, coll. Li, Qi-Shi [李奇石]; DNA voucher HBUMM08300a. HBUMM8453, many fms, Shifangzhen [石坊镇], Wenxian, Gansu Province, near point (33.00509°N, 104.579061°E), 2021-IX-27, coll. Chen, Z.-G. HBUMM8455, Town of Wenxian, Gansu Province, near point (32.944361°N, 104.679819°E), 2020-VIII, coll. Chen, Z.-G.

###### Additional information of shell.

In juveniles, regularly arranged fine threads and elongate granules are present on the protoconch, where in adults such sculpture is difficult to be observed because of erosion. Spiral grooves are present after the fourth whorl or only on apical body whorl, but are absent in umbilical side. The teleoconch has distinct and regularly arranged ribs, among which there is no fine threads.

###### Distribution.

Gansu: Wenxian (type locality).

###### General anatomy.

Eversible head wart not prominent, perhaps due to specimen of not full maturity. Jaw arcuate, with five projecting ribs.

###### Anatomy of genital organs.

Penial sheath moderately long, covering ~ 1/4 of penis. Penis slightly expanded distally. Accompanied with two or three lower pilasters, at proximal 1/2 of penis two thin penial pilasters fusing into one Y-shaped fork. Fine pilasters of distal 1/2 penis weaving into delicate net. Near epiphallic opening numerous fine pilasters merging into ~ 4 short but thick folds. Vas deferens narrow throughout. Vagina between atrium and dart sac not elongated. Vagina between dart sac and insertion of bursa copulatrix duct ~ 1/2 length of dart sac. Dart sac ~ 2/3 length of penis. Information of love dart unknown. Accessory sac small. Mucous glands ~ 4, each complicatedly branched. Proximal accessory sacs two, symmetrical on dart sac, dorsally separated and ventrally touching, internally smooth (without pilasters), each with an opening leading to dart chamber near dart chamber opening. Bursa copulatrix duct of even diameter.

##### ﻿ Laeocathaica carinifera

Taxon classificationAnimaliaStylommatophoraCamaenidae

(H. Adams, 1870)

11B03A67-FB60-5D42-95C2-8D764E82CEA7

**Figure 9.**
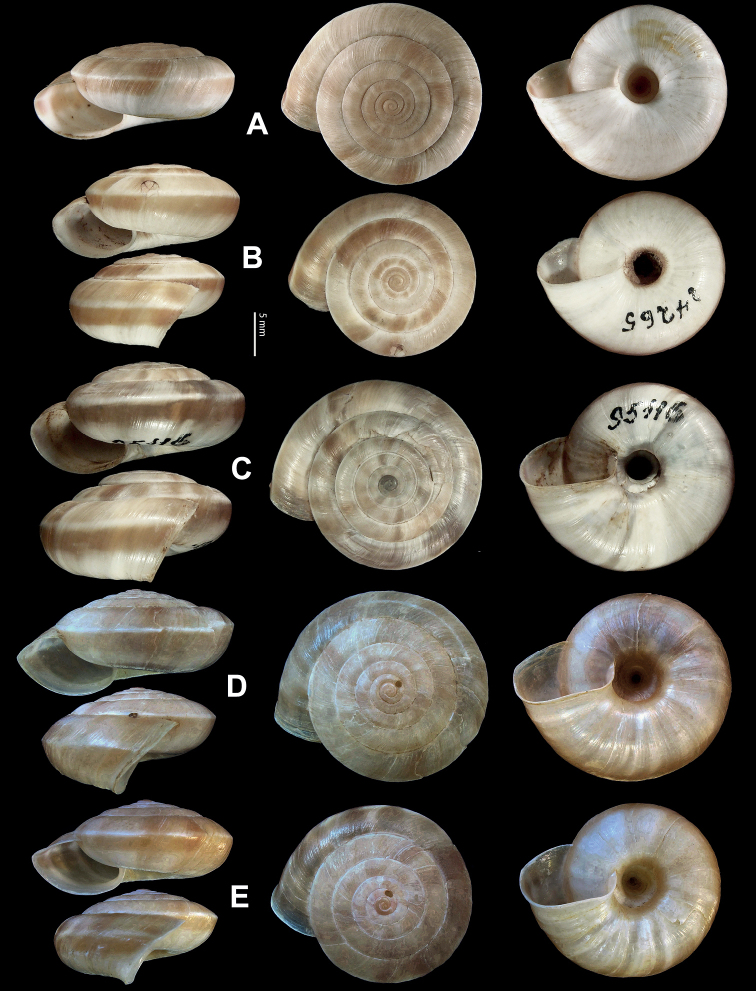
*Laeocathaicacarinifera* (H. Adams, 1870) **A**NHMUK V. W. McAndrew coll. Acc. no. 1563 **B**SMF 24265 **C**SMF 95116 **D** HBUMM08443-spec.1 **E** HBUMM08443-spec.2.

**Figure 10.**
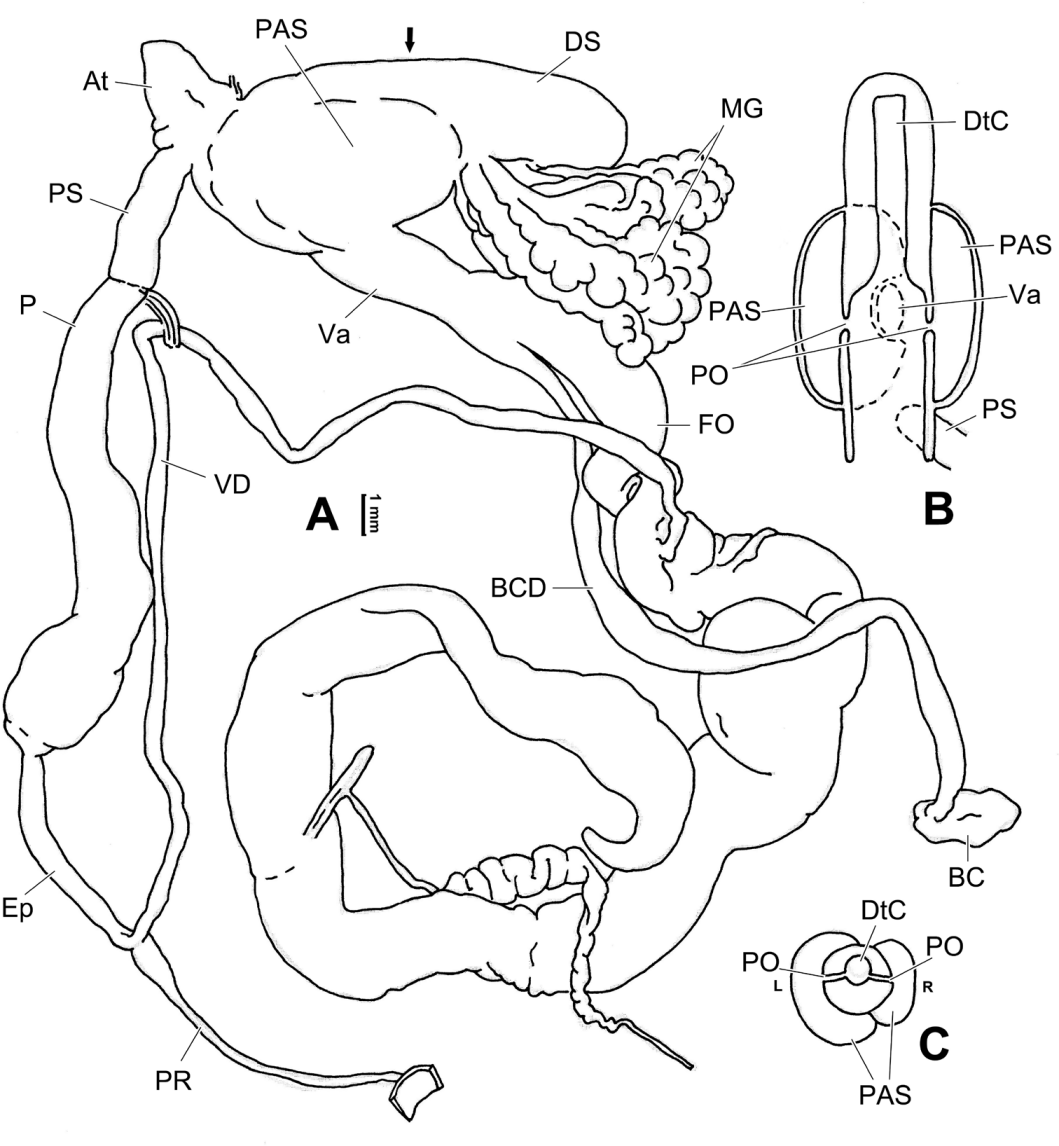
Genital anatomy of *Laeocathaicacarinifera* (H. Adams, 1870), HBUMM08443-spec.1 **A** general view **B** apical view of dart sac apparatus **C** cross-section of dart sac at the position arrowed in (**A**). Abbreviations: AS – accessory sac; At – atrium; BC – bursa copulatrix; BCD – bursa copulatrix duct; DS – dart sac; DtC – a chamber containing love dart; Ep – epiphallus; FO – free oviduct; L – left; MG – mucous glands; P – penis; PAS – proximal accessory sac; PO – opening of proximal accessory sac leading to dart chamber; PR – penial retractor muscle; PS – penial sheath; R – right; Va – vagina; VD – vas deferens.

Figs 2A, C , 9 , 10 , 43G , 45I, J , 50B , 51 , Tables 1 , 3

Helix (Plectotropis) christinae var. carinifera H. Adams, 1870: 377.
Helix subsimilis Deshayes, 1874: 10, pl. 2, figs 28, 29; – Heude 1882: 22, pl. 20, fig. 18; – Standen 1905: 231.
Helix (Plectotropis) subsimilis – Ancey 1883: 7, pl. 2, figs 28, 29.
Helix christinae var. carinifera – Gredler 1884: 264 (= Helixsubsimilis Deshayes, 1873); – Möllendorff 1884: 351.
Helix (Cathaica) christinae var. subsimilis – Pilsbry 1892: 214, pl. 49, figs 29–33.
Helix (Cathaica) subsimilis – Kobelt 1894: 713, pl. 203, figs 1–3.
Laeocathaica christinae carinifera – Richardson 1983: 78.
Laeocathaica (Laeocathaica) subsimilis – Zilch 1968: 175; – Richardson 1983: 79; Wu and Chen 1998: 107.
Cathaica subsimilis – Yen 1938: 456.
Laeocathaica subsimilis subsimilis – Yen 1939: 148, pl. 15, fig. 28.
Laeocathaica subsimilis – Möllendorff 1899: 44; – Wiegmann 1900: 96, pl. 3, figs 84–87; – Yen 1942: 283; – Gude 1902: 6; – Chen and Zhang 2004: 313, fig. 299.
Laeocathaica (Laeocathaica) filippina – Wu 2004: fig. 17.
Laeocathaica carinifera – Páll-Gergely et al. 2022: 50, fig. 12.

###### Museum material.

*Laeocathaicasubsimilis* (Deshayes, 1874): NHMUK V. W. McAndrew coll. Acc. no. 1563. SMF 95116, one fms; Szetschwan, Noerdl. der Stadt Juanj-juanj, China; ex Mus. Petersb. 1905 ex Potanin. Det. Möllendorff. SMF 24265, one fms and one near matured; Yang-dsy-gebiet; ex Heude. Slg. O. v. Möllendorff. SMF 294292, two juvs (the third one, a fms of *L.filippina*); Sytshuan, China; Slg. C. Bosch ex H. Rolle. SMF 24261, two fms; Liu-ba-ting, Shen-hsi, China; ex Potanin 451, Slg. O. v. Möllendorff. SMF 24258, three fms; O-Sytshuan; ex Möllendorff. Slg. W. Kobelt. SMF 24257, four fms; O-Sy-tshuan; ex. B. Schmacker, Slg. O. v. Möllendorff. SMF 24262, four fms; Sy-tshuan, Chung-king, W-M-China; ex Möllendorff, 1890, Slg. O. Böttger. SMF 24255, four fms; O-Sy-tshuan; ex L. Fuchs, Slg. O. v. Möllendorff. SMF 95712, one fms; Prov. Sze-chuen; Slg. C. R. Böttger, 1905. SMF 294294, one fms. China; Slg. C. Bosch ex Hermann Rolle. The species of following specimens are not decided: SMF 24260, one fms (with aperture not full matured), Kwan-juon-hszien (= Guangyuan) [广元县], Prov. Sze-Csuen, China, ex Kormos (Budapest). SMF 24256, three fms, Zw. Guan-yuan u. Dshau-hoa [昭化], Potanin 275, Slg. O. v. Möllendorff. SMF 24264, four fms, Lue-feng-kou bei Guan-yuan, ex Potanin 270, Slg. O. v. Möllendorff.

###### New material.

HBUMM00006, 4 fma, 4 juvs; Xinjianxiang [新建乡], Nanchong [南充市], Sichuan Province, near point (30.963475°N, 105.842133°E); 1964-V-20, coll. Chen, De-Niu. [陈德牛]. HBUMM00228, 1 fma and 2 subadults; Beibei [北碚], Chongqing [重庆], near point (29.809307°N, 106.411375°E); 1980, coll. Chen D.-N. HBUMM01573, 7 fma, 1 dissected; Muping [穆坪镇], Baoxingxian [宝兴县], Sichuan Province; the first hill to the south, between point A (1090 m a.s.l., 30.360333°N 102.7255°E) and point B (1021 m a.s.l., 30.341389°N 102.783722°E); limestone, 2 cm- thick litter layer, typical local vegetation type, seminatural, nearby cultivated with maize; 2003-VII-23, coll. Wu, Min [吴岷]. HBUMM03118, 4 juvs; Changping [长坪], Wanzhou [万州], Chongqing, near point (30.448543°N, 108.260695°E); 2003-VII-16, coll. Yuan, C. etc. HBUMM04064, many fma, 1 dissected; Meizixiang [梅子乡], Fengjiexian [奉节县], Chongqing, near point (430 m a.s.l., 31.178028°N, 109.341583°E); mixed rocks of slate and sandstone, semimature environment, very thin litter layer, nearby with eucalyptus trees; 2004-VII-18, coll. Wu, M. and Wu, Qin [吴琴]; DNA voucher HBUMM05121. HBUMM04162, numerous fma, 1 dissected, Shibaozhai [石宝寨], Zhongxian [忠县], Chongqing, 238 m a.s.l., 30.423083°N, 108.183972°E, shrubs and weed, purple sandy stone, humid, 2004-VII-16, coll. Wu, M., Wu, Q. and Qi, Gang [齐钢]; DNA voucher HBUMM05103. HBUMM04217, 10 fma; Tiejiacun [铁甲村], Jiangnanxiang [江南乡], Fengjiexian, Chongqing, 1005 m a.s.l., 30.956444°N, 109.468306°E; limestone, grass and shrubs, humid; 2004-VII-19, coll. Wu, M. and Wu, Q. HBUMM04219: many fma; same coll. data as HBUMM04064. HBUMM04232, several fma and juvs; near Xinmincun [新民村], Jiangnanxiang, Fengjiexian, Chongqing, 980 m a.s.l., 30.932583°N, 109.455639°E; mixed rocks of sandstone and limestone, grass and shrubs, humid, semi-farmland; 2004-VII-19, coll. Wu, M. and Wu, Q. HBUMM04235, 2 fma and 1 juv, 1 dissected, near town of Wuxixian [巫溪县], Chongqing, 339 m a.s.l., 31.402111°N, 109.634722°E; limestone, grass and shrubs, bare earth without litter layer; 2004-VII-22, coll. Wu, M.; DNA voucher HBUMM05131). HBUMM08443, 4 fma and 1 subadult, 4 dissected, Baidicheng [白帝城], Fengjie County, Chongqing, two kilometers away from the point (241 m a.s.l., 31.020194°N,, 109.472833°E), slate and sandy stones, woods with shrubs and trees, humid, 2004-VII-20, coll. Wu, M.

###### Distribution.

Sichuan: Nanchong, Baoxing. Chongqing: Beibei, Changshouxian [长寿县], Fengduxian [丰都县] (type locality: Fungsiang gorge = Fengxiangxia [风箱峡]), Fengjiexian, Kaixian [开县], Liangpingxian [梁平县], Pengshuixian [彭水县], Shizhuxian [石柱县], Wanzhou, Wushanxian [巫山县], Wuxi, Yunyangxian [云阳县], Zhongxian. Shaanxi: Lueyang [略阳].

###### Additional information of shell.

On adult shell the protoconch is granulate with thick granules of ~ 40 µm long, each of which looks like a hump in a pit. The obscurity of such granules is caused by erosion or weathering. Spiral grooves are absent throughout the shell. On teleoconch the growth lines are low but distinct.

###### General anatomy.

Eversible head wart weak. Jaw arcuate, with four or five projecting ribs.

###### Anatomy of genital organs.

Penial sheath moderately long, covering ~ 1/4–1/3 of penis. Penis somewhat expanded distally. A pair of penial internal pilasters fusing into one Y-shaped fork at proximal 1/3, and another pair fusing into one Y-shaped fork at proximal 1/2 of penis; some other low pilasters variably present. Distally inside penis, numerous fine pilasters merging into 6–8 short but thick folds near opening of epiphallus. Vas deferens narrow throughout. Vagina between atrium and dart sac not elongated. Vagina between dart sac and insertion of bursa copulatrix duct approximately as long as or slightly shorter than 2/3 of dart sac. Dart sac ~ 1/2 of penis. Love dart ~ 2 mm long. Accessory sac spherical, internally almost solid, inserting into dart sac medially, opening to distal dart chamber. Mucous glands six or seven, each simply or complicatedly branched. Proximal accessory sacs two, asymmetrical, left one larger than right one, dorsally separated and ventrally touching, with internal pilasters, each with an opening leading to proximal dart chamber. Bursa copulatrix duct of even diameter. Bursa copulatrix ovate, small.

###### Remarks.

See *Laeocathaicastenochone*.

##### ﻿ Laeocathaica christinae

Taxon classificationAnimaliaStylommatophoraCamaenidae

(H. Adams, 1870)

3D2C7EE7-3974-5D7A-8B96-38B4A770FCC4

**Figure 11.**
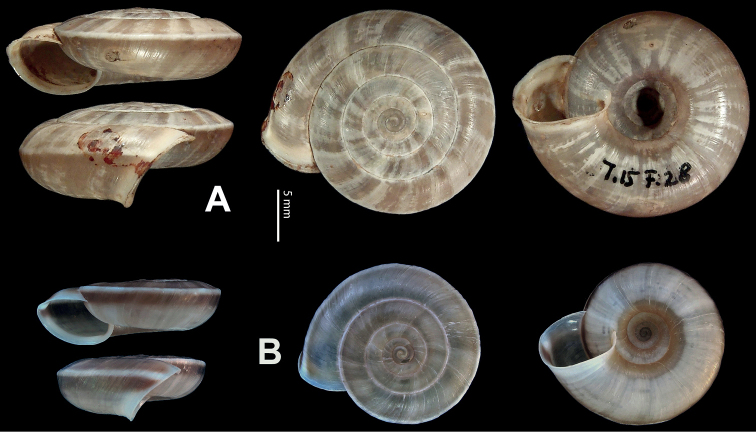
**A***Laeocathaicaminwui* Páll-Gergely, 2022, SMF 24255a **B***L.christinae* (H. Adams, 1870), HBUMM01251-spec.1.

**Figure 12.**
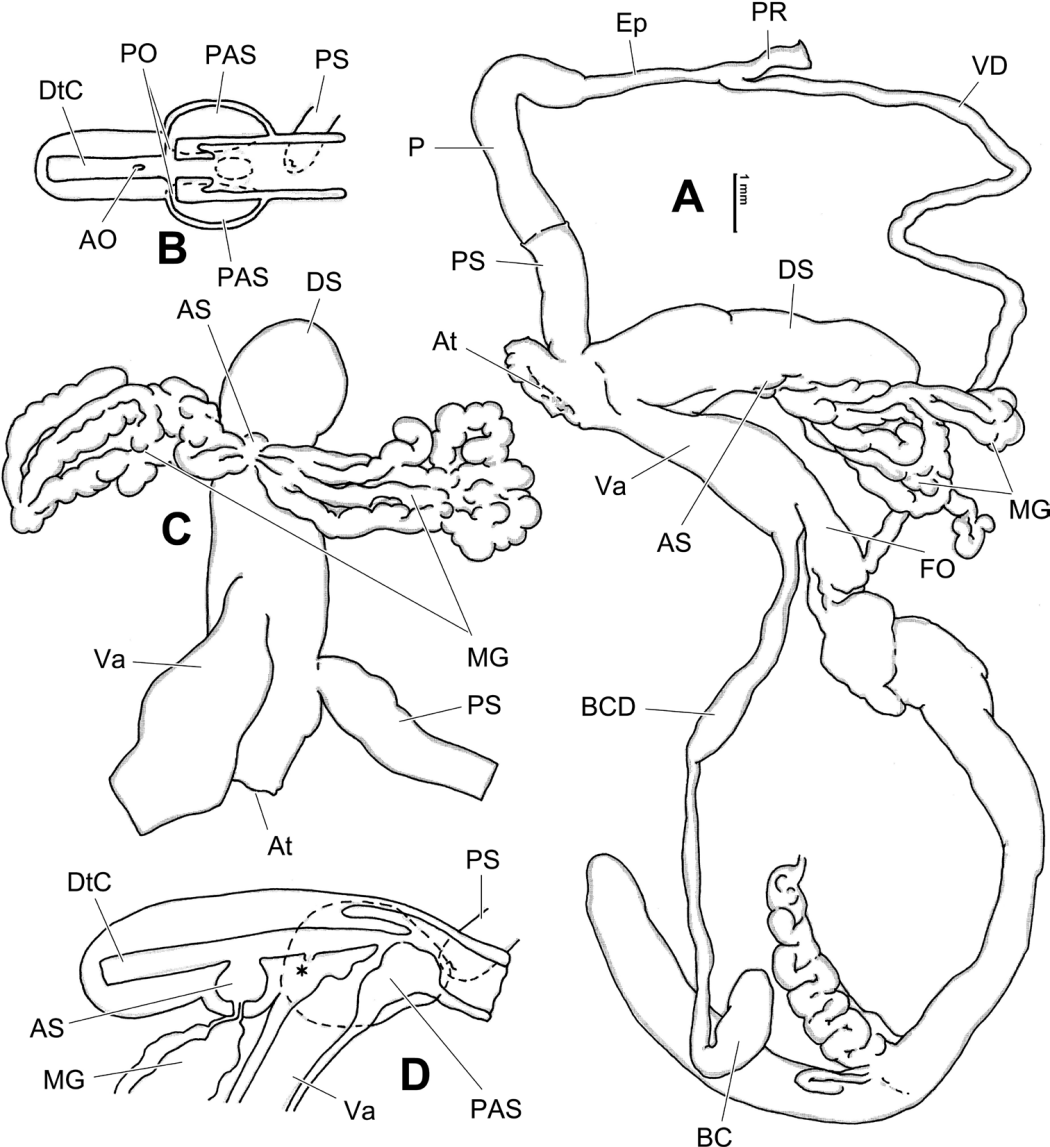
Genital anatomy of *Laeocathaicachristinae* (H. Adams, 1870) **A, C** HBUMM01251-spec.1 **B, D** HBUMM04204-spec.1 **A** general view **B–D** apical, ventral, and left views of dart sac apparatus. Abbreviations: AO – opening of accessory sac leading to dart chamber; AS – accessory sac; At – atrium; BC – bursa copulatrix; BCD – bursa copulatrix duct; DS – dart sac; DtC – a chamber containing love dart; Ep – epiphallus; FO – free oviduct; MG – mucous glands; P – penis; PAS – proximal accessory sac; PO – opening of proximal accessory sac leading to dart chamber or dart sac chamber; PR – penial retractor muscle; PS – penial sheath; Va – vagina; VD – vas deferens. Asterisk * indicates the opening of proximal accessory sac.

Figs 2A , 11B , 12 , 42C , 45G, H ; Table 1

Helix (Plectotropis) christinae H. Adams, 1870: 377, pl. 27, fig. 4, 4a.
Helix (Plectopylis) christinae – Ancey, 1882: 44.
Helix christinae – Dohrn, 1881: 596, pl. 174, figs 17–19; – Möllendorff 1884: 340, 351; – Gredler 1884: 264; – Heude 1885: 111, pl. 29, fig. 4; – Pilsbry 1892: 213, pl. 57, figs 15–17.
Laeocathaica christinae – Möllendorff 1899: 43; – Gude 1902: 6; – Yen 1942: 283, pl. 28, fig. 197; – Chen and Zhang 2004: 334, fig. 326; – Páll-Gergely et al. 2022: 54, fig. 13.
Laeocathaica (Laeocathaica) christinae christinae – Zilch 1968: 173.
Laeocathaica (Laeocathaica) christinae – Richardson 1983: 77.

###### Museum material.

SMF 61056. Labeled with *L.subsimilis*: SMF 50089, two fms; W-Hupei, China; Slg. g. Maegele ex V. Gredler, 1906.

###### New material.

HBUMM01251a, many fma, 1 fma dissected; Wuyuandong [无源洞] and nearby, Badong [巴东], Hubei Province, 254 m a.s.l., 31.027667°N, 110.420139°E, broad-leaved woods, limestone, humid, 2003-VIII-20, coll. Wu, M. HBUMM04204, Wuyuandong and nearby, Badong, Hubei Province, 255 m a.s.l., 31.026806°N, 110.422111°E, broad-leaved woods, limestone, humid, 2004-VII-31, coll. Wu, M., Wu, Q., Qi, G.

###### Distribution.

Chongqing: Fengjie (type locality: Fungsiang gorge (= Fengxiangxia)); Hubei: Badong.

###### Additional information of shell.

Tiny granules (~ 5 – ~ 50 µm) are densely and radially arranged throughout the protoconch. The erosion or weathering of sculpture on adult shell is not observed. Spiral grooves are absent throughout the shell. On teleoconch the growth lines are usually indistinct.

###### General anatomy.

Eversible head wart weakly present. Jaw arcuate, with four projecting ribs.

###### Anatomy of genital organs.

Penial sheath covering ~ 1/3 of penis. Penis tubular and equally thick. Inside penis, 5–6 narrow or thick pilasters present, Y-shaped fork formed by adjacent pilasters absent. Fine pilasters on distal end of penis merging into seven or eight thick folds. Vas deferens narrow throughout. Vagina between atrium and dart sac not elongated. Vagina between dart sac and insertion of bursa copulatrix duct ~ 1/2 length of dart sac. Dart sac slightly shorter than penis. Love dart ~ 5 mm long, apically 2-bladed. Accessory sac small but externally distinct, internally empty but spatially narrow, inserting into dart sac at middle part, opening to distal dart chamber. Mucous glands four (HBUMM04204-spec.14, spec.16) or five (HBUMM04204-spec.13, HBUMM01251a-spec.1), each complicatedly branched. Proximal accessory sacs two, symmetrical or the left one slightly larger (HBUMM04204-spec.14), dorsally and ventrally separated, each with a distal opening leading to dart chamber near its opening. Bursa copulatrix duct of even diameter. Bursa copulatrix ovate, small.

###### Remarks.

This species is not known from NW Sichuan, so the specimens distributed in “Wentschun” [Wenchuan 汶川] identified as *Cathaicachristinae* by Blume (1925; followed by Yen 1938), may indicate some other sinistral species. However, it could also be an erroneous site naming, as all field records except this indicate that no *Laeocathaica* species is distributed in Wenchuan.

##### ﻿ Laeocathaica dejeana

Taxon classificationAnimaliaStylommatophoraCamaenidae

(Heude, 1882)

EDEC6696-AE01-5529-B921-4DC0DE788243

Figs 2A , 13A, B

Helix dejeana Heude, 1882: 21, pl. 20, fig. 17; – Möllendorff 1884: 340, 352.
Helix (Cathaica) dejeana – Pilsbry 1892: 215, pl. 49, figs 36–38.
Cathaica dejeana – Möllendorff, 1899: 73; – Yen 1938: 446; – Yen 1939: 141, pl. 14, fig. 46; – Yen 1942: 278.
Cathaica (Pseudiberus) dejeana – Gude 1902: 8.
Cathaica (Campylocathaica) dejeana – Richardson 1983: 51; – Chen and Zhang 2004: 270, fig. 255.
Laeocathaica dejeana – Wu 1999: 148, fig. 6.13-1; – Chen and Zhang 2004: 337, fig. 330; – Páll-Gergely et al. 2022: 56, fig. 15.

###### Museum material.

SMF 23919, lectotype. NHMUK 1902.5.13.1-2, 2 juv shells; W. Setchuan, W. China.

**Figure 13.**
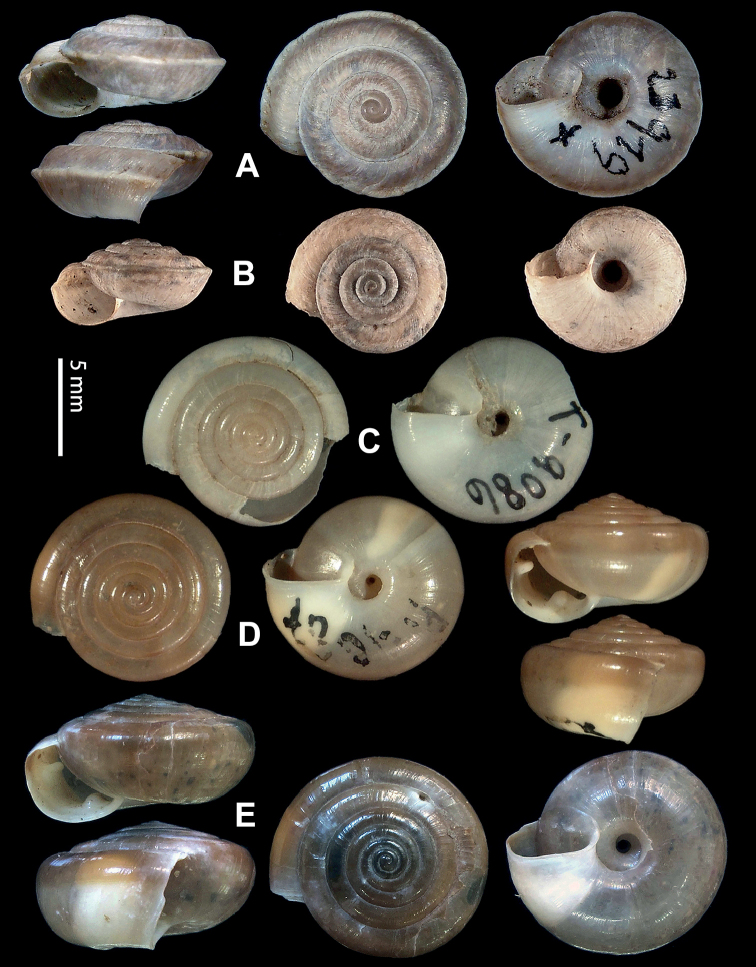
**A, B***Laeocathaicadejeana* (Heude, 1882) **A**SMF 23919, lectotype **B** NHMUK1902.5.13.1-2 **C–E***L.dityla* Möllendorff, 1899 **C**SMF 9086, lectotype **D**SMF 9087, paratype **E** HBUMM00698-spec.1.

###### Distribution.

Sichuan: western region (type locality) including Kangding [康定].

###### Additional information of shell.

Protoconch has small granules. Spiral grooves are absent throughout the shell. Shell surface has tiny scales.

###### Anatomy of genital organs.

Penis as long as dart sac, distally expanded. Vas deferens narrow throughout. Vagina between atrium and dart sac not elongated. Vagina between dart sac and insertion of bursa copulatrix duct ~ 1/2 length of dart sac. Love dart ~ 2 mm long, apically 2-bladed. Accessory sac small but distinguishable from outside, inserting into dart sac at middle part, opening to distal dart chamber. Mucous glands two, each complicatedly branched. Bursa copulatrix duct of equal thickness, as long as dart sac. Bursa copulatrix ovate (Wu 1999: 148, fig. 6.13-1).

##### ﻿ Laeocathaica distinguenda

Taxon classificationAnimaliaStylommatophoraCamaenidae

Möllendorff, 1899

3A59635F-074F-5FB3-9F75-68F7374EB38D

Figs 2A, D , 15 , 16 , 41D, E , 46A, B , 50A , 51 ; Tables 1 , 3

Laeocathaica distinguenda Möllendorff, 1899: 93, pl. 5, fig. 6; – Gude 1902: 6; – Yen 1939: 149, pl. 15, fig. 32; – Yen 1942: 283; – Chen and Zhang 2004: 317, fig. 305.
Laeocathaica subsimilis distinguenda – Wiegmann 1900: 99, pl. 3, fig. 88.
Laeocathaica (Laeocathaica) distinguenda – Zilch 1968: 173; – Richardson 1983: 78.
Laeocathaica amdoana – Páll-Gergely et al. 2022: 38, fig. 5A.

###### Museum material.

SMF 8959, lectotype; Thal des Pui-ho (= Baishuijiang) [白水江] b. Lum-du, Sy-tshuan, China; ex Potanin 906, Slg. O. v. Möllendorff. SMF 95024, paratype; SO-Gansu, NW-China; Slg. C. R. Böttger 1904 (ex Möllendorff!). “*Aegistadistinguenda*”, ZIN RAS No. 7, 3 fms. ZIN RAS No. 22, 1 fma and 1 subadult, between Nanping [南坪 = Jiuzhaigou 九寨沟县] and Sung-Pan (= Songpan), coll. Beresowskij, 1894, det. Möllendorff.

###### New material.

HBUMM00674, numerous fma; Jianshanxiang [尖山乡], Wenxian, Gansu Province, 840 m a.s.l., 33.043291°N, 104.869226°E; 1998-IV-29, coll. Chen, D.-N. HBUMM05473, many fms, Jidushan [基督山] near Baishuijiang, Wenxian, Gansu Province, near point (32.94936°N, 104.699043°E); limestone and broad-leaved woods; 2006-IX-27; coll. Wu, M., Liu, J.-M., Zheng, W. and Gao, Lin-Hui [高林辉]. HBUMM05434, HBUMM05436, 2 fma dissected, Yuxushan [玉虚山], Wenxian, Gansu Province, 32.957259°N, 104.689152°E, shrubs and slate, 2006-IX-27, coll. Wu, M., Liu, J.-M., Zheng, W. and Gao, L.-H. HBUMM05415, 2 fma; hill foot of Yuxushan, Wenxian, Gansu Province; slate and shrubs, dry; 2006-IX-27, coll. Wu, M., Liu, J.-M., Zheng, W. and Gao, L.-H. HBUMM05417, numerous fma, 3 fma dissected, same data as HBUMM05436; DNA voucher HBUMM05407. HBUMM05434, 2 fma; same data as HBUMM05436. HBUMM05479, 2 fma; bank of Baishuijiang, Wenxian, Gansu Province, 32.946383°N, 104.685343°E, limestone and loess, broad-leaved woods, 2006-IX-27, coll. Wu, M., Liu, J.-M., Zheng, W. and Gao, L.-H.; DNA voucher HBUMM05442. HBUMM05554, 1 fma, 4 subadults. HBUMM05565a, many fma. HBUMM05571, many fma and subadults. HBUMM05576b, 1 fma: Hengdan [横丹], Wenxian, Gansu Province; north side of Baishuijiang River, along 212 Guodao, near point (32.864025°N, 104.859517°E); hillside, bushes; 2006-IX-29; coll. Wu, M., Liu, J.-M., Zheng, W. and Gao, L.-H. HBUMM06536, not dissected; town of Wenxian, Gansu Province, 1024 m a.s.l., 32.941111°N, 104.668889°E, 2011-VIII-08, coll. Wu, M., Xu, Qin [徐沁], Budha, Prem; DNA voucher HBUMM06535. HBUMM06579, 2 fma, 4 fms and 1 juv (DNA voucher HBUMM06578). HBUMM06556, 2 fms: Fengchengsi [风成寺], Jiuzhaigouxian, Sichuan Province, 1515 m a.s.l., 33.256280°N, 104.238386°E; 2011-VIII-14, coll.Wu, M., Xu, Q. and Buhda, P.; DNA voucher HBUMM06555. HBUMM06646, 1 fma; Wenxian, Gansu Province, 1269 m a.s.l., 33.091944°N, 104.360833°E; 2011-VIII-9, coll. Wu, M., Xu, Q. and Buhda, P. HBUMM06736, 1 subadult; Eastern bank of Baishuijiang, Lihuacun [梨花村], Jiuzhaigouxian, Sichuan Province, 1425 m a.s.l., 33.267222°N, 104.234722°E; 2011-VI-14, coll.Wu, M., Xu, Q. and Buhda, P. HBUMM08148, 1 fma and several subadults; slope near river, Wenxian, Gansu Province, 807 m, 32.847111°N, 104.88775°E; grass and a few shrubs, on branches and rock cliff; 2017-VIII-6, coll. Sheng, X.-F. etc. HBUMM08429, 1 fma dissected; toward Danpuzhen [丹堡镇] on national road 212, Wenxian, Gansu Province, near 32.872636°N, 104.784372°E, on slope, 2019-X-13, coll. Li, Q.-M.; DNA voucher HBUMM08429a. CZG202008-w5, 8 fms, Zhangzhazhen [彰扎镇], Jiuzhaigouxian, Sichuan Province, near point (33.30674°N, 103.877345°E); 2020-VIII, coll. Chen, Z.-G. CZG202008-w9, Heihexiang [黑河乡], Jiuzhaigouxian, Sichuan Province, near (33.54941°N, 104.041691°E); 2020-VIII, coll. Chen, Z.-G. CZG202008-w10, 5 fms, whitish shells; Shijiba [石鸡坝], Wenxian, Gansu Province, near point (33.067936°N, 104.456784°E); 2020-VIII, coll. Chen, Z.-G.

###### Distribution.

Gansu: Wenxian (type locality), Wudu (type locality), Zhouquxian (type locality); Sichuan: Jiuzhaigouxian (type locality).

###### Additional information of shell.

The protoconch has the widespread granules each of which is ~ 20 – ~ 40 µm long and looks like a hump embedded in a shallow socket. All granules are present on densely arranged fine radial threads. Spiral grooves are shallowly present. The growth lines on teleoconch are more or less distinct but are irregularly arranged.

###### General anatomy.

Eversible head wart present. Jaw arcuate, with 4–7 projecting ribs.

###### Anatomy of genital organs.

Penial sheath moderately long, covering ~ 1/6–1/4 of penis. Penis slightly expanded distally. Inside penis, two penial pilasters forming one Y-shaped fork at proximal 1/3 penis (only indistinct in HBUMM05417); a rather thick pilaster that is made up of numerous thread-like longitudinal folds occupying proximal 1/3–2/3 of penis. Pilasters of median to distal penis weaving into a delicate net that bears regularly arranged diamond-shaped papillae, which are usually lost perhaps because of bad specimen condition. Distally inside penis, fine pilasters merging into 6–9 short and more or less thick folds near opening of epiphallus. Vas deferens narrow throughout. Vagina between atrium and dart sac not elongated. Vagina between dart sac and insertion of bursa copulatrix duct ~ 1/4 length of dart sac. Dart sac ~ 1/2 length of penis. Love dart ~ 9 mm long, apically 2-bladed. Accessory sac small but distinguishable from outside, internally solid, inserting into dart sac at middle part, opening to distal dart chamber. Mucous glands 4 (HBUMM08429, each complicatedly branched) – 12 (HBUMM05479, simply branched). Proximal accessory sacs two, symmetrical or the right one slightly larger, separated dorsally and ventrally, with a few internal pilasters, each with an opening leading to dart chamber near opening of dart chamber. Bursa copulatrix duct of even diameter. Bursa copulatrix ovate.

###### Remarks.

The sculpture on protoconch and teleoconch of this species (Fig. 46A, B) resembles that of *Laeocathaicaamdoana* (Fig. 46G, H). However, in *Laeocathaicadistinguenda*, as in the most congeners of *Laeocathaica*, the part of the vagina between atrium and dart sac apparatus is not elongated.

For more comments, see *Laeocathaicatropidorhaphe*.

##### ﻿ Laeocathaica dityla

Taxon classificationAnimaliaStylommatophoraCamaenidae

Möllendorff, 1899

4015175F-1E97-5075-BF10-C42A914EE6EE

**Figure 14.**
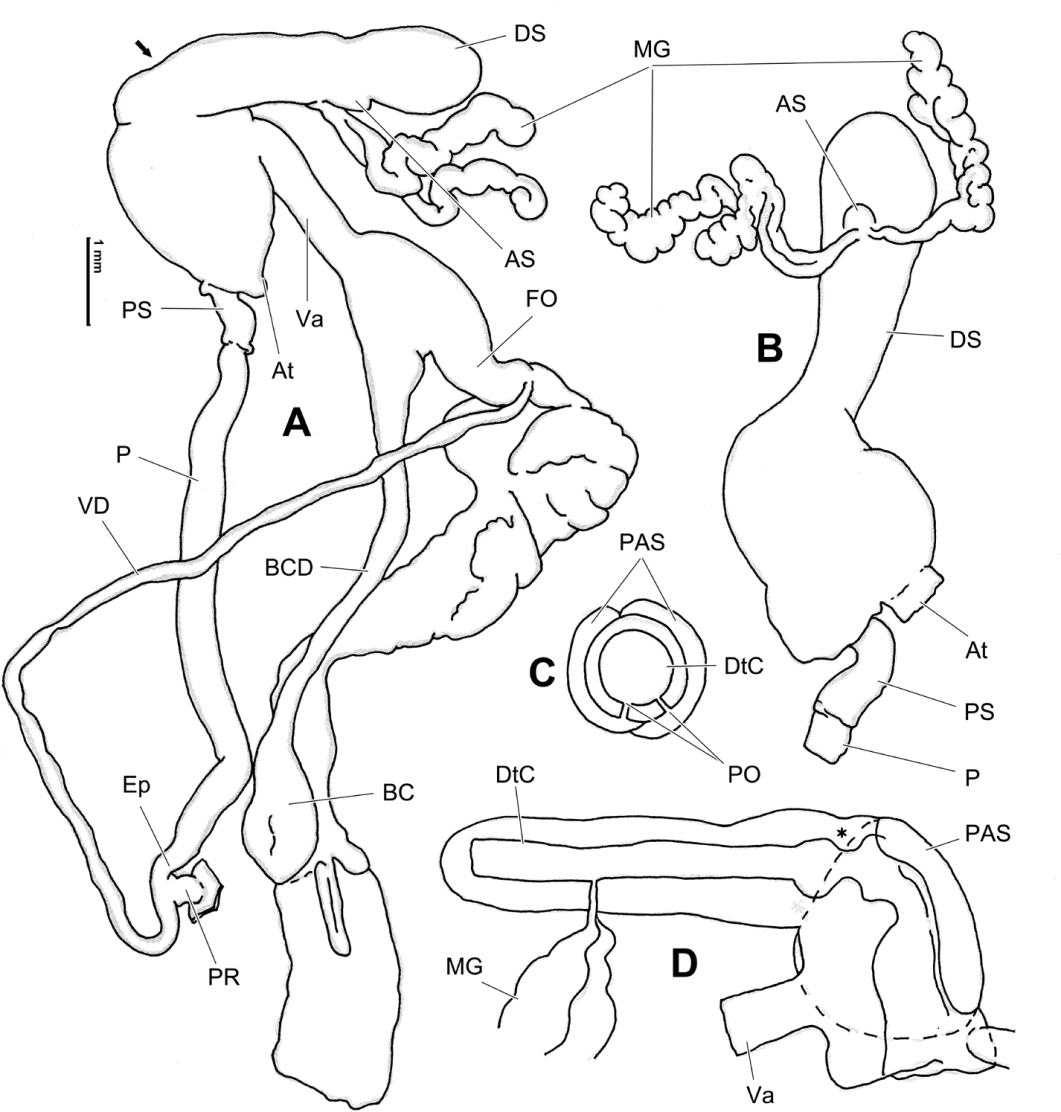
Genital anatomy of *Laeocathaicadityla* Möllendorff, 1899, HBUMM00698-spec.1 **A** general view **B** ventral view of dart sac apparatus **C** cross-section of dart sac at the position arrowed in (**A**) **D** left view of dart sac apparatus. Abbreviations: AS – accessory sac; At – atrium; BC – bursa copulatrix; BCD – bursa copulatrix duct; DS – dart sac; DtC – a chamber containing love dart; Ep – epiphallus; FO – free oviduct; MG – mucous glands; P – penis; PAS – proximal accessory sac; PO – opening of proximal accessory sac leading to dart chamber; PR – penial retractor muscle; PS – penial sheath; Va – vagina; VD – vas deferens. Asterisk * indicates the opening of proximal accessory sac.

Figs 2A , 3 , 13C–E , 14 , 41G, H , 45C, D ; Table 1

Laeocathaica dityla Möllendorff, 1899: 99, pl. 6, fig. 8; – Sturany 1900: 22, pl. 1, figs 4–6; – Wiegmann 1900: 121, pl. 3, figs 108–111; – Gude 1902: 6; – Yen 1939: 150, pl. 15, fig. 42; – Chen and Zhang 2004: 332, fig. 324; – Páll-Gergely et. al.: 57, fig. 16.
Laeocathaica (Laeocathaica) dityla – Zilch 1968: 174; Richardson 1983: 78.

###### Museum material.

SMF 9086, lectotype. SMF 9087, 1 paratype. SMF 9088, 1 paratype. ZIN RAS No.1, 1 fms.

###### New material.

HBUMM00532, 2 juvs; Xinglongcun [兴隆村],Zhongzhaixiang [中寨乡], Wenxian, Gansu Province, near point (33.232415°N, 104.419075°E); 1998-V-19, coll. Chen, D.-N. and Zhang, Guo-Qing [张国庆] HBUMM00698, 1 fma and several juvs, 1 fma dissected; Daigusicun [代古寺村], Diebuxian [迭部县], Gansu Province, near point (34.004495°N, 103.940717°E), 1998-V-10, coll. Chen, D.-N. and Zhang, G.-Q. HBUMM05644, 4 fma and several fms; Jiaogongzhen, Wudu, Gansu Province (33.57°N, 104.64°E), broken limestone rocks, 2006-X-02, coll. Liu, J.-M. and Zheng, W. HBUMM05674, 1 fms; eastern bank of Bailongjiang [白龙江], Lianghekou [两河口], Dangchangxian [宕昌县], Gansu Province, near point (33.697332°N, 104.493015°E); limestone; 2006-X-02, coll. Zheng, W. and Gao, L.-H. HBUMM05685, 1 fma and 1 juv; western bank of Bailongjiang, Lianghekou, Dangchangxian, Gansu Province, near point (33.696791°N, 104.49129°E); limestone; 2006-X-02, coll. Zheng, W. and Gao, L.-H. HBUMM05710, 1 fms; Guantingzhen [官亭镇], Dangchangxian, Gansu Province, near point (33.82428°N, 104.538282°E); limestone hills, along 212 Guodao; 2006-X-3, coll. Zheng, W. and Liu, J.-M. HBUMM08449, 3 fms; Shijiba, Wenxian, Gansu Province, near point (33.067582°N, 104.457685°E); 2020-VIII, coll. Chen, Z.-G.

###### Distribution.

Gansu: Wenxian, Diebuxian, Dangchangxian, Zhouquxian (type locality).

###### Additional information of shell.

Tiny, low and sparsely arranged granules (~ 10 µm) on the smooth protoconch are present but difficult to be observed because of erosion or weathering. Spiral grooves are absent throughout the shell. On teleoconch the growth lines are not observed except on the part just following the protoconch.

**Figure 15.**
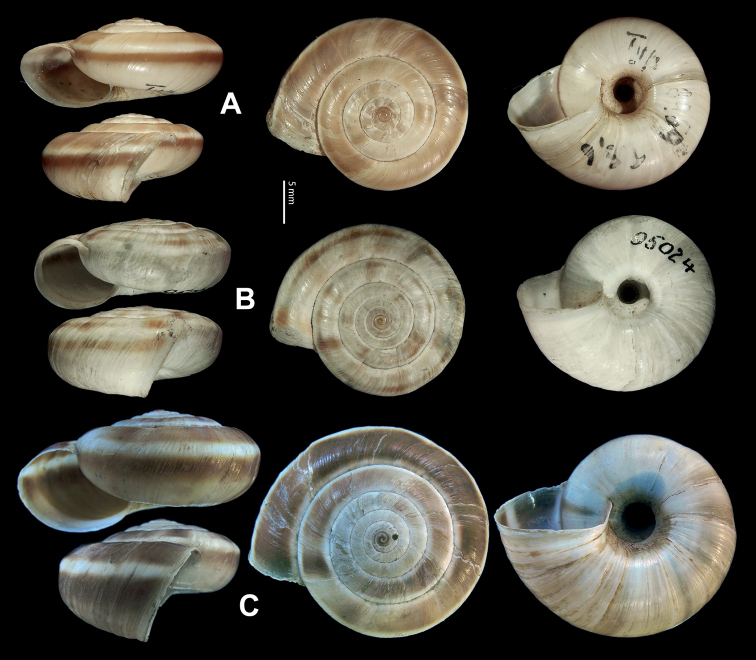
*Laeocathaicadistinguenda* Möllendorff, 1899 **A**SMF 8959, lectotype **B**SMF 95024 **C** HBUMM054361.

###### General anatomy.

Eversible head wart present but not prominent. Jaw arcuate, with four or five projecting ribs.

###### Anatomy of genital organs.

Penial sheath rather short, covering ~ 1/8 of penis. Penis tubular and equally thick. Inside penis, two penial internal pilasters forming one Y-shaped fork at proximal 1/2 of penis, accompanied with another two thicker pilasters. Distal 1/2 of penis occupied by numerous isolated minute diamond-shaped papillae. Vas deferens narrow throughout. Vagina between atrium and dart sac not elongated. Vagina between dart sac and insertion of bursa copulatrix duct approximately as long as dart sac. Dart sac ~ 1/2 length of penis. Love dart ~ 4 mm long, apically 2-bladed and then rounded. Accessory sac small but distinguishable from outside, internally empty but spatially narrow, inserting into dart sac medially, opening to distal dart chamber. Mucous glands two (HBUMM00698-spec.2, spec.3) or three (HBUMM00698-spec.1), each a single tube. Proximal accessory sacs two, dorsally and ventrally touching, without internal pilasters, each with an opening leading to dart chamber near its opening. The right proximal accessory sac somewhat larger and with thicker wall than the left one. Bursa copulatrix duct basally slightly expanded. Bursa copulatrix ovate.

###### Remarks.

This species has a very unique shell, but the anatomy of the terminal genitalia coincides with those of the congeners.

##### ﻿ Laeocathaica dolani

Taxon classificationAnimaliaStylommatophoraCamaenidae

(Pilsbry, 1934)

B4855E0D-AD83-51DD-8796-DCBD854C48EA

Figs 2A , 5C , 7 , 42A, B , 47C, D , 52F ; Table 1

Cathaica (Laeocathaica) dolani Pilsbry, 1934: 16, pl. 3, fig. 4, 4a–c.
Cathaica dolani – Yen, 1938: 446.
Laeocathaica (Laeocathaica) dolani – Richardson 1983: 78.
Laeocathaica dolani – Chen and Zhang 2004: 335, fig. 328; – Páll-Gergely et al. 2022: 59, fig. 18A.

###### New material.

HBUMM00069, 6 fma, 3 fma dissected; Kangding,Sichuan Province, near 29.995149°N, 101.967008°E, 1964-VII-16, coll. Chen, D.-N. HBUMM08439, Manai [马耐], Badixiang [巴底乡], Danbaxian (= Danba), Sichuan Province, 31.11276°N 101.885295°E, 2008-VII-18, coll. Di, Zhi-Yong [邸智勇].

**Figure 16.**
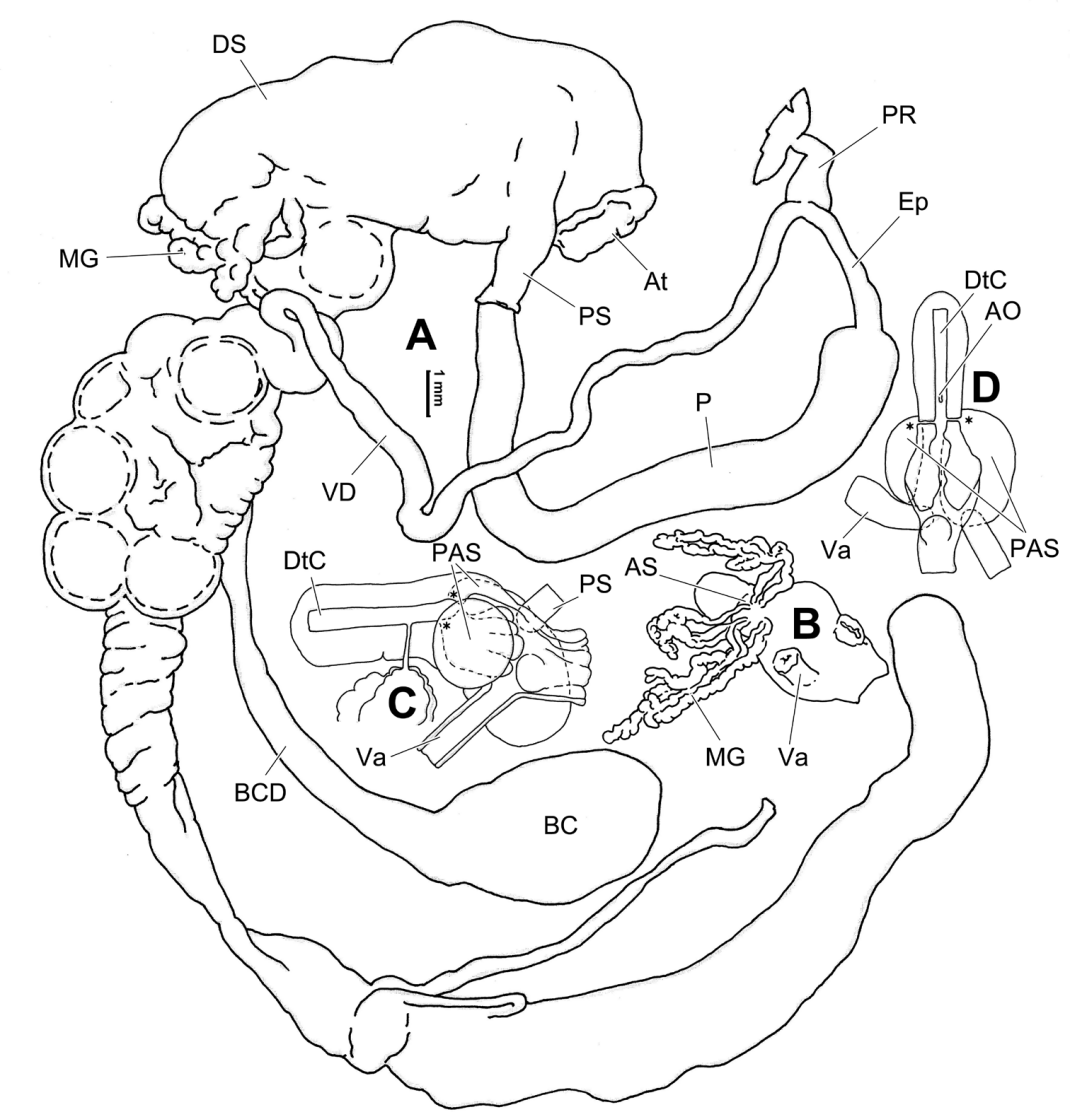
Genital anatomy of *Laeocathaicadistinguenda* Möllendorff, 1899 **A, B** HBUMM05436 **C, D** HBUMM05417 **A** general view **B–D** ventral, left, and apical views of dart sac apparatus. Abbreviations: AO – opening of accessory sac leading to dart chamber; AS – accessory sac; At – atrium; BC – bursa copulatrix; BCD – bursa copulatrix duct; DS – dart sac; DtC – a chamber containing love dart; Ep – epiphallus; MG – mucous glands; P – penis; PAS – proximal accessory sac; PO – opening of proximal accessory sac leading to dart chamber; PR – penial retractor muscle; PS – penial sheath; Va – vagina; VD – vas deferens. Asterisk * indicates the opening of proximal accessory sac.

###### Distribution.

Sichuan: Kangding, Danbaxian (type locality: Rumichangu).

###### Additional information of shell.

The shell is covered with radially arranged low scales of variable length (~ 25 – ~ 300 µm long) everywhere. The size (esp. length) of scales increase from protoconch to body whorl. Spiral grooves are absent throughout the shell.

###### General anatomy.

Eversible head wart present but not prominent. Jaw arcuate, with 3–6 projecting ribs.

###### Anatomy of genital organs.

Penial sheath covering ~ 1/4–1/3 of penis. Penis short, thick, distally expanded. Inside penis, two thick and prominent pilasters fusing into one Y-shaped fork at proximal 1/3–1/2 penis, accompanied with ~ 6 narrower or equally thick pilasters nearby. Pilasters on distal end of penis not merging into thick folds. Vas deferens narrow throughout. Vagina between atrium and dart sac not elongated. Vagina between dart sac and insertion of bursa copulatrix duct ~ 1/2 length of dart sac. Dart sac approximately as long as penis. Love dart ~ 5 mm long, apically with cross-section rhombic. Accessory sac small, internally empty, inserting into dart sac at middle part, opening to distal dart chamber. Mucous glands two (HBUMM00069-spec.1–3), each complicatedly branched. Proximal accessory sac one, large, on right side of dart sac, with an opening leading to dart chamber near its opening. Bursa copulatrix duct basally slightly expanded. Bursa copulatrix elongate, ovate.

###### Remarks.

In *Laeocathaica*, *Laeocathaicadolani* is the only species with only one proximal accessory sac. In addition, this species is unique in the genus that it keeps periostracum scales throughout its lifetime.

##### ﻿ Laeocathaica filippina

Taxon classificationAnimaliaStylommatophoraCamaenidae

(Heude, 1882)

D0AFF918-D783-54B8-BEE3-669023620D3B

**Figure 17.**
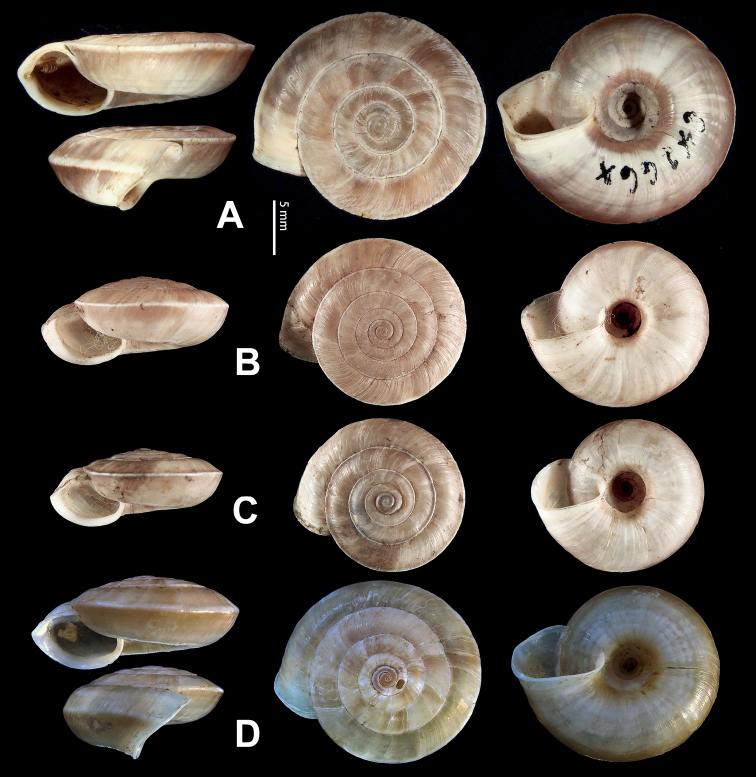
*Laeocathaicafilippina* (Heude, 1882) **A**SMF 24266b **B, C**NHMUK ex Salisburg Acc. No. 2044 **D** HBUMM04166-spec.1.

**Figure 18.**
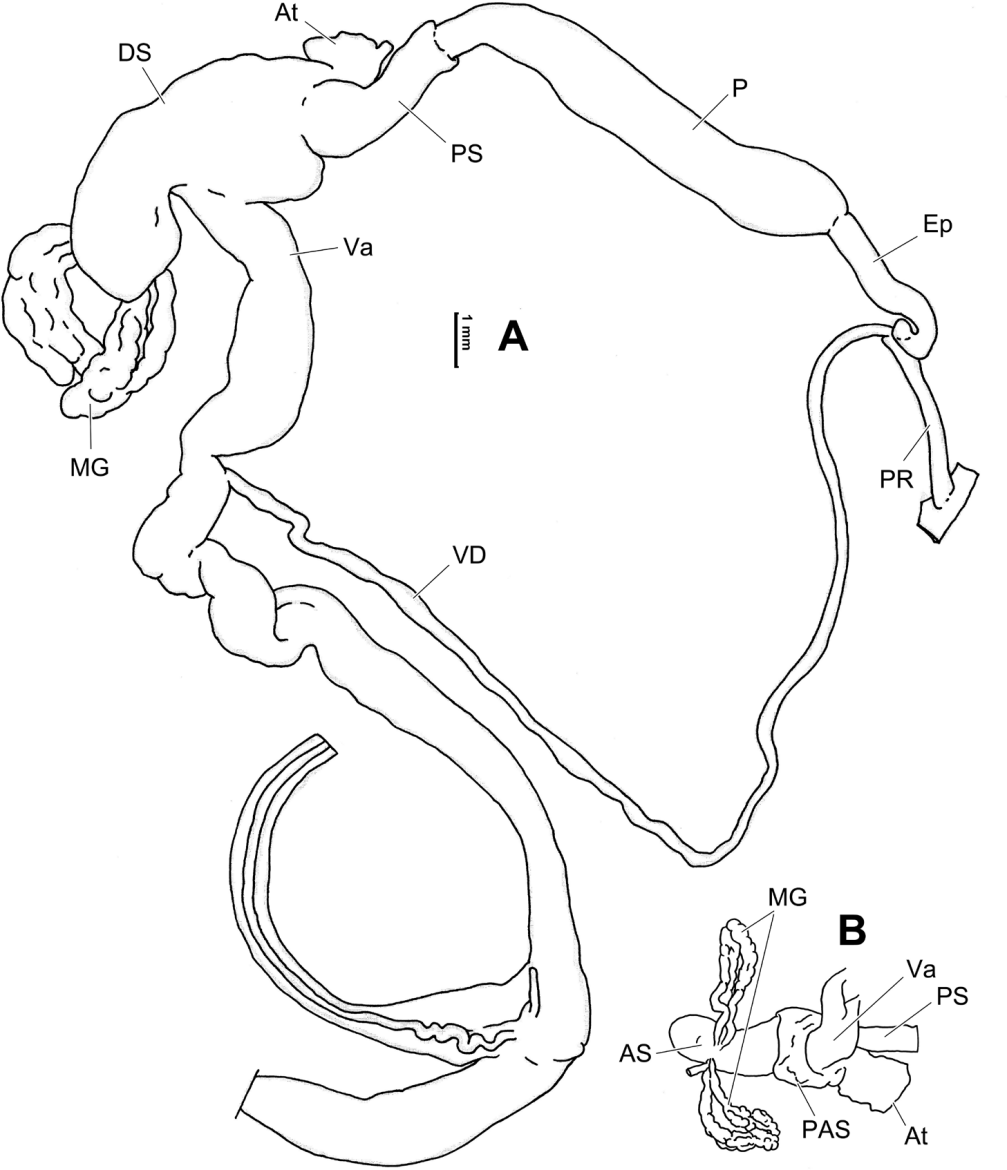
Genital anatomy of *Laeocathaicafilippina* (Heude, 1882), HBUMM04166-spec.1 **A** general view **B** ventral view of dart sac apparatus. Abbreviations: AS – accessory sac; At – atrium; DS – dart sac; Ep – epiphallus; MG – mucous glands; P – penis; PAS – proximal accessory sac; PR – penial retractor muscle; PS – penial sheath; Va – vagina; VD – vas deferens.

Figs 2A , 17 , 18 , 41C , 48G , 48H , 51 ; Tables 1 , 3

Helix filippina Heude, 1882: 23, pl. 20, fig. 19; – Möllendorff 1884: 340, 352; – Ancey 1885: 114; – Standen: 231.
Helix (Plectopylis) subchristinae Ancey, 1882: 44.
Helix (Plectotropis) subchristinae – Ancey 1883: 8.
Helix subchristinae – Möllendorff 1884: 340, 352; – Ancey 1885: 114.
Helix subsimilis var. filippina – Gredler 1884: 264.
Helix (Cathaica) filippina – Pilsbry 1892: 214, pl. 49, figs 34, 35.
Laeocathaica subchristinae – Gude 1902: 6.
Laeocathaica filippina – Möllendorff 1899: 88; – Gude 1902: 6; – Yen 1935: 44; – Páll-Gergely et al. 2022: 59, fig. 19.
Laeocathaica subsimilis filippina – Yen 1939: 148, pl. 15, fig. 29.
Laeocathaica (Laeocathaica) christinae filippina – Zilch 1968: 173; – Richardson 1983: 79.

###### Museum material.

SMF 24266b, four fms; Badung, Hubei, China. SMF 24226, three fms; W-Hupei, China; Slg. K. Hashagen. SMF 24257, two fms; Changyang [长阳], Sytchuan, China; ex B. Schmacker, 1893, Slg. O. Böttger. SMF 95118, five fms; Badung, Hubei, China; Slg. C. R. Böttger, 1904. SMF 24266a, three fms; Badung, Hubei, China; ex Möllendorff, Slg. W. Kobelt. SMF 294296, three fms; Changyang, China; Slg. C. Bosch, ex Sowerby+Fulton. SMF 294295, one fms; Changyang, China; Slg. Ehrmann, ex Sowerby+Fulton. SMF 24227, one fms (labeled with *L.subsimilis*); China; ex? SMF 294292, one fms (*L.filippina*) and two juvs (*L.carinifera*); Sytshuan, China; Slg. C. Bosch ex H. Rolle. NHMUK ex Salisburg Acc. No. 2044.

###### New material.

HBUMM01256b, Fuhusi, Emei [峨嵋], Sichuan; sandstone, humid; 2003-VII-26, coll. Wu, M.; DNA voucher HBUMM01256b. HBUMM04166, numerous fma, 5 fma dissected; Baidicheng, Fengjie County, Chongqing, two kilometers away from the point (241 m a.s.l., 31.020194°N, 109.472833°E); mixed rocks of slate and sandy stones, woods with shrubs and trees, humid; 2004-VII-20, coll. Wu, M; DNA voucher HBUMM05097.

###### Distribution.

Hubei: Badong (type locality), Yichang (Changyang); Sichuan (Emei); Chongqing: Fengjie.

###### Additional information of shell.

Sculpture on the protoconch was present but indistinct because of erosion or weathering. Spiral grooves are absent throughout the shell.

###### General anatomy.

Eversible head wart low. Jaw arcuate, with four or five projecting ribs.

###### Anatomy of genital organs.

Penial sheath moderately long, covering ~ 1/4–1/3 of penis. Penis slightly expanded distally. A pair of penial internal pilasters fusing into one Y-shaped fork at proximal 1/3; ~ 2 larger pilasters present nearby. Distally inside penis, numerous fine pilasters merging into 6 to 8 short but thick folds near opening of epiphallus. Vas deferens narrow throughout. Vagina between atrium and dart sac not elongated. Vagina between dart sac and insertion of bursa copulatrix duct approximately equal to or slightly shorter than 2/3 length of dart sac. Dart sac ~ 1/2 length of penis. Love dart ~ 2 mm long. Accessory sac spherical, internally almost solid, inserting into dart sac medially, opening to distal dart chamber. Mucous glands 5–7, each complicatedly branched. Proximal accessory sacs two, symmetrical, dorsally separated and ventrally touching, with a few internal pilasters, each with an opening leading to proximal dart chamber. Bursa copulatrix duct of even diameter. Bursa copulatrix ovate, small.

###### Remarks.

This species has a spire of variable height, from flatness to dome shape. The umbilicus of this species is obviously narrower than that of *Laeocathaicachristinae*. Furthermore, this species has a pair of ventrally touching proximal accessory sacs instead of ventrally separated ones, which are observed in *L.christinae*.

##### ﻿ Laeocathaica hisanoi

Taxon classificationAnimaliaStylommatophoraCamaenidae

Páll-Gergely, 2022

9F722DA4-05FC-5AC6-AA0E-61A29B25BA8C

Laeocathaica hisanoi Páll-Gergely in Páll-Gergely et al. 2022: 68, fig. 20D.

###### Examined specimens.

None.

###### Distribution.

S Gansu (type locality).

##### ﻿ Laeocathaica leucorhaphe

Taxon classificationAnimaliaStylommatophoraCamaenidae

Möllendorff, 1899

5F71F6FF-285A-58D7-9582-BC75CE2AB91D

Fig. 19A

Laeocathaica leucorhaphe Möllendorff, 1899: 95, pl. 6, fig. 2; – Gude 1902: 6; – Yen 1939: 149, pl. 15, fig. 36; – Chen and Zhang 2004: 323, fig. 312; – Páll-Gergely et al. 2022: 63, fig. 18B.
Cathaica leucorhaphe – Yen, 1938: 446.
Laeocathaica (Laeocathaica) leucorhaphe – Zilch 1968: 174; – Richardson 1983: 78.

###### Museum material.

SMF 9073, lectotype; Sm Tung-ho [铜河 = Daduhe River 大渡河], W-Sytshuan; ex Potanin 312b, Slg. O. v. Möllendorff.

###### Distribution.

W Sichuan (type locality: Daduhe River).

###### Additional information of shell.

The protoconch is finely granulate. Growth lines are indistinct. Spiral grooves are only more distinct on umbilicus side near aperture. Bottom-umbilicus transition changes gently. Peristome is somewhat sinuate.

**Figure 19.**
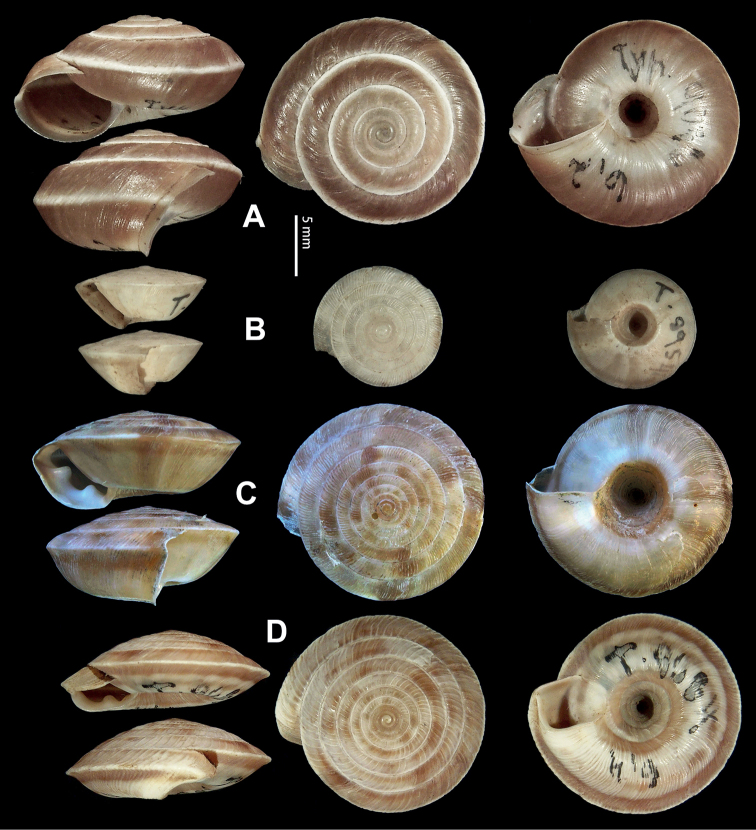
**A***Laeocathaicaleucorhaphe* Möllendorff, 1899, SMF 9073, lectotype **B, C***L.odophora* Möllendorff, 1899 **B**SMF 8954, holotype **C** HBUMM08430-spec.1 **D***L.pewzowi* Möllendorff, 1899, SMF 9084, lectotype.

##### ﻿ Laeocathaica minwui

Taxon classificationAnimaliaStylommatophoraCamaenidae

Páll-Gergely, 2022

27571AFE-5E2C-5F4A-9612-9370C9D14F83

Figs 2A , 11A

Laeocathaica minwui Páll-Gergely in Páll-Gergely et al. 2022: 64, fig. 21.

###### Museum material.

SMF 95019, holotype, one fms; Prov. Sze-chuan, West China; Slg. C. R. Böttger ex Möllendorff ex L. Fuchs. SMF 24255a, five fms; O. Sy-tshuan. Ex. L. Fuchs, Slg. O. v. Möllendorff. The following lots of specimens labeled with *L.subsimilis*: SMF 6920: one fms; Sy-tshuan, W-China; Ex. Möllendorff, Slg. W. Kobelt. SMF 24263, one fms; Kao-cha-hien; Ex. B. Schmacker 1893, Slg. O. Böttger. SMF 42563, two fms; Chang-yang, China; ex B. Schmacker, Slg. O. Böttger.

###### Distribution.

Sichuan (type locality); Hubei (type locality).

###### Additional information of shell.

The protoconch is finely granulate. Spiral grooves are very weakly present. Bottom-umbilicus transition changes gently. Peristome is somewhat sinuate.

##### ﻿ Laeocathaica odophora

Taxon classificationAnimaliaStylommatophoraCamaenidae

Möllendorff, 1899

2681AAF6-5FF2-53D2-ABC0-F606FEDA24B3

**Figure 20.**
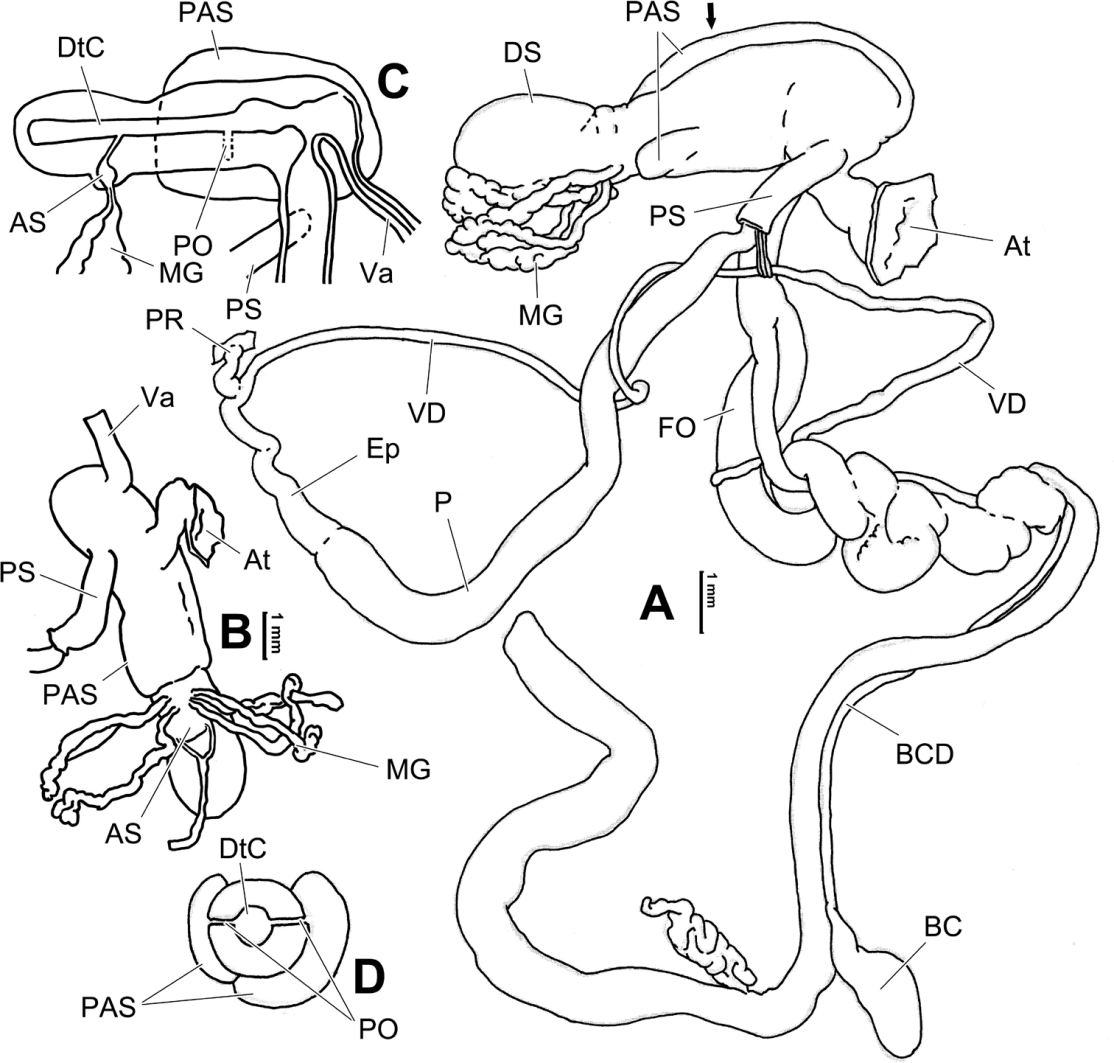
Genital anatomy of *Laeocathaicaodophora* Möllendorff, 1899, HBUMM08430-spec.1 **A** general view **B** ventral view of dart sac apparatus **C** left view of dart sac apparatus **D** cross-section of dart sac at the position arrowed in (**A**). Abbreviations: AS – accessory sac; At – atrium; BC – bursa copulatrix; BCD – bursa copulatrix duct; DS – dart sac; DtC – a chamber containing love dart; Ep – epiphallus; FO – free oviduct; MG – mucous glands; P – penis; PAS – proximal accessory sac; PO – opening of proximal accessory sac leading to dart chamber; PR – penial retractor muscle; PS – penial sheath; Va – vagina; VD – vas deferens.

Figs 2A , 3 , 19B , 19C , 20 , 42E , 47A , 47B , 51 ; Tables 1 , 3

Laeocathaica odophora Möllendorff, 1899: 97, pl. 6, fig. 6; – Yen 1939: 149, pl. 15, fig. 39; – Chen and Zhang 2004: 328, fig. 318; – Páll-Gergely et al. 2022: 63, fig. 18B.
Laeocathaica (Laeocathaica) odophora – Zilch 1968: 174; – Richardson 1983: 78.
Laeocathaica (Laeocathaica) odontophora – Richardson 1983: 78.

###### Museum material.

SMF 8954, holotype, juv; Dshie-dshou [阶州= Wudu], S-Gansu. Slg. O. v. Möllendorff.

###### New material.

HBUMM00566a, 5 juvs; Luotuoxiang [骆驼巷], Bikou [碧口], Wenxian, Gansu Province, 32.827686°N, 105.064656°E; 1998-IV-25, coll. Chen, D.-N. and Zhang, G.-Q. HBUMM04612, 1 fma and 1 juv; 650 m away from Bikou Bridge, Wenxian, Gansu Province; 1998-VI-24, coll. Zhang, Xue-Zhong [张学忠]. HBUMM08158, 3 juvs; slope near river, Wenxian, Gansu Province, 807 m, 32.847111°N, 104.88775°E; grass and a few shrubs, on branches and rock cliff; 2017-VIII-6, coll. Sheng, X.-F., etc.; DNA voucher HBUMM08158a. HBUMM08430, 1 fma, 1fms, 1 juv; 1 fma dissected; near Fengjiaba [冯家坝], national road 212, Wenxian, Gansu Province, near point (33.08672°N, 104.826572°E), on slope, 2019-X-12, coll. Li, Q.-M.; DNA voucher HBUMM08430a.

###### Distribution.

Gansu: Wudu (type locality), Wenxian.

###### Additional information of shell.

The protoconch is smooth except some fine threads at the beginning and the end of the whorls. Spiral grooves are absent. On teleoconch between two adjacent ribs there are several indistinct fine threads.

###### General anatomy.

Eversible head wart present but not prominent. Jaw arcuate, with seven projecting ribs.

###### Anatomy of genital organs.

Penial sheath short, covering ~ 1/6 of penis. Penis tubular and equally thick. Inside penis, ~ 5 low pilasters present proximally, two high pilasters fusing into one Y-shaped fork at proximal 1/4. Fine pilasters on distal end of penis not merging into thick folds. Vas deferens narrow throughout. Vagina between atrium and dart sac not elongated. Vagina between dart sac and insertion of bursa copulatrix duct ~ 1/2 length of dart sac. Dart sac ~ 1/2 length of penis. Love dart ~ 4.5 mm long, apically 2-bladed. Accessory sac small but externally distinguishable, internally solid, inserting into dart sac at distal 1/3, opening to dart chamber. Mucous glands 5 (HBUMM08430-spec.1), each singly tubular or simply branched. Proximal accessory sacs two, asymmetrical with the right one distinctly larger, apically separated and ventrally touching, each centrally with an opening (rounded, diameter ~ 0.2 mm) leading to proximal dart chamber. Bursa copulatrix duct of even diameter. Bursa copulatrix ovate, small.

###### Remarks.

This species has a pair of asymmetrical and ventrally touching proximal accessory sacs that is rarely observed in the genus. In comparison, its conchologically similar species *Laeocathaicazhengpingliui* Wu, sp. nov. has a pair of symmetrical proximal accessory sacs that are ventrally separated from each other.

For more comments, see *Laeocathaicazhengpingliui* Wu, sp. nov.

##### ﻿ Laeocathaica pewzowi

Taxon classificationAnimaliaStylommatophoraCamaenidae

Möllendorff, 1899

086CA1D6-0858-5F29-BEB1-67E8DB90E77E

**Figure 21.**
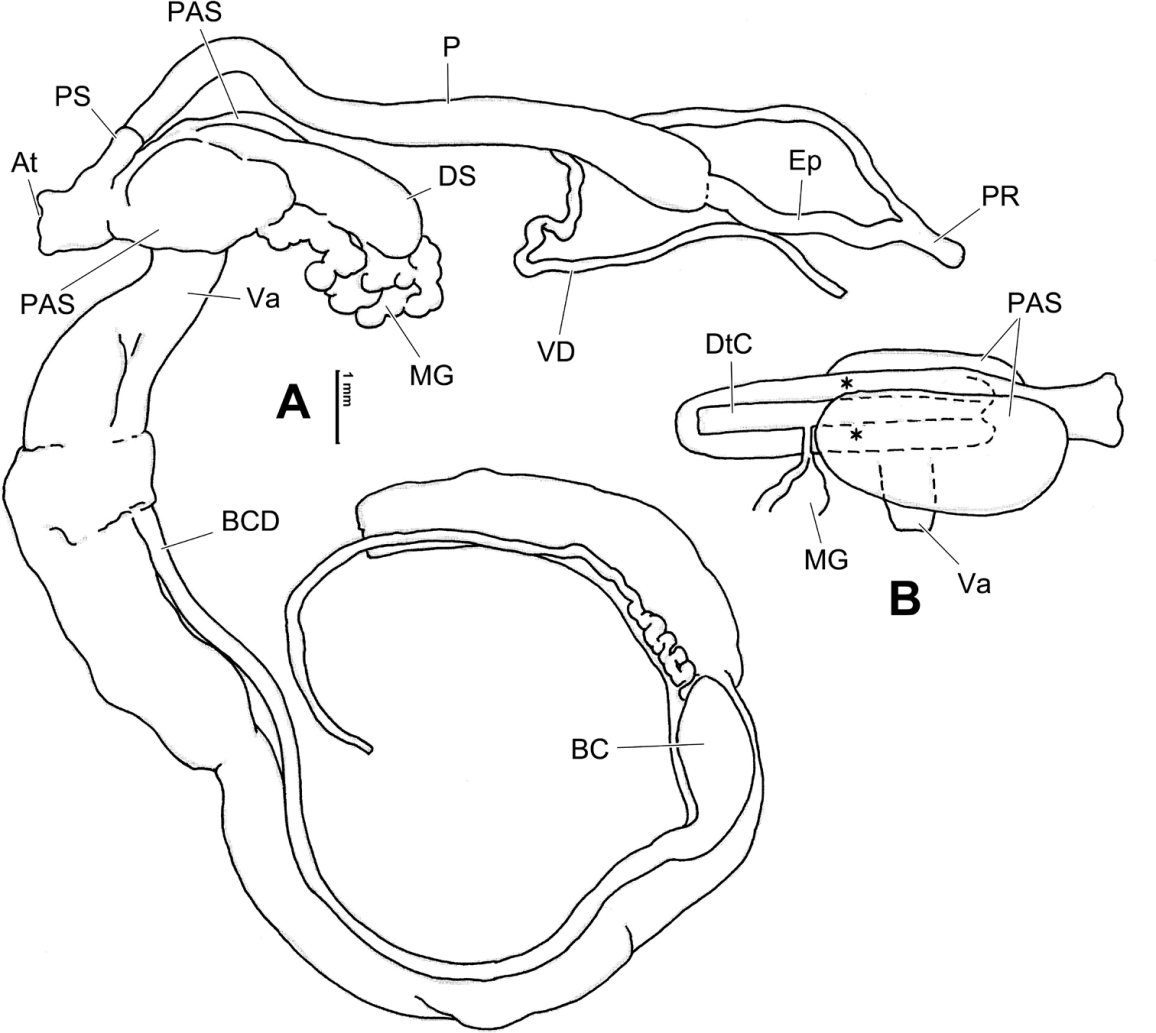
Genital anatomy of *Laeocathaicapewzowi* Möllendorff, 1899, ZIN RAS No. 4, “*Aegistapewzowi* Schalf.” **A** general view **B** left view of dart sac apparatus. Abbreviations: AS – accessory sac; At – atrium; BC – bursa copulatrix; BCD – bursa copulatrix duct; DS – dart sac; DtC – a chamber containing love dart; Ep – epiphallus; MG – mucous glands; P – penis; PAS – proximal accessory sac; PR – penial retractor muscle; PS – penial sheath; Va – vagina; VD – vas deferens. Asterisk * indicates the opening of proximal accessory sac.

Figs 2A , 3 , 19D , 21 , 42F , 47G, H ; Table 1

Laeocathaica pewzowi Möllendorff, 1899: 98, pl. 6, fig. 4, 4a; – Sturany 1900: 22, pl. 2, figs 25–28; – Wiegmann 1900: 115, pl. 3, fig. 104; – Möllendorff 1901: 302; – Gude 1902: 6; – Yen 1939: 150, pl. 15, fig. 40; – Schileyko 2004: 1686, fig. 2174A; – Chen and Gao 2004: 329, fig. 320 (an erroneous figure that should be a Laeocathaicaodophora); – Páll-Gergely et al. 2022: 67, fig. 23C, D.
Laeocathaica (Laeocathaica) pewzowi – Zilch 1968: 174; – Richardson 1983: 78.

###### Museum material.

SMF 9084, lectotype; Wen-hsien, S-Gansu; Potanin 248, 661, 793, Slg. O. v. Möllendorff. SMF 9085, paratypes, four fms; same data as lectotype. SMF 24268, Hung-dan (= Hengdan) b. Wen-hsien; Slg. O. v. Möllendorff, ex Berezowski. ZIN RAS No. 4, “*Aegistapewzowi* Schalf.” 2 fma, locality unknown, coll. Potanin, 1885-IX-8, det. Möllendorff.

###### New material.

HBUMM08452, 1 fms and 4 juvs, Shangdezhen [尚德镇], Wenxian, Gansu Province, near point (32.907414°N, 104.76994°E); 2020-VIII, coll. Chen, Z.-G.

###### Distribution.

Gansu: Wenxian (type locality).

###### Additional information of shell.

Protoconch is smooth and has no granules, perhaps caused by erosion or weathering. Teleoconch has rough ribs between which fine threads are present. Spiral grooves are absent throughout.

###### General anatomy.

Head with flat but distinct eversible head wart. Jaw arcuate, with four projecting ribs.

###### Anatomy of genital organs.

Penial sheath covering ~ 1/9 of penis. Penis tubular and distally thicker. Inside penis, ~ 4 high parallel pilasters on proximal 2/3, at proximal ~ 1/4 two weak pilasters fusing into one Y-shaped fork. Fine pilasters on distal end of penis merging into ~ 7 thin or thick folds. Vas deferens narrow throughout. Vagina between atrium and dart sac not elongated. Vagina between dart sac and insertion of bursa copulatrix duct ~ 1/2 length of dart sac. Dart sac ~ 2/5 length of penis. Accessory sac tiny, internally solid, inserting into dart sac at middle part, opening to dart chamber. Mucous glands four, each singly tubular or simply branched. Proximal accessory sacs two, of similar size and symmetrical, apically separated and ventrally touching, each distally with an opening leading to proximal dart chamber. Bursa copulatrix duct of even diameter. Bursa copulatrix long-ovate.

###### Remarks.

The species has the most common pattern of the dart sac apparatus of the genus, i.e., two equally- sized and ventrally touching proximal accessory sacs, each with an opening leading to the dart chamber; this has been observed in ten of the 21 species anatomically studied herein. This species can be immediately recognized by its particular shell morphology.

##### ﻿ Laeocathaica phaeomphala

Taxon classificationAnimaliaStylommatophoraCamaenidae

Möllendorff, 1899

8B5466A0-C804-56DC-9BBB-5323E948384B

Figs 2A , 3 , 22 , 23 , 42D , 48E, F , 51 , 52D ; Tables 1 , 3

Laeocathaica phaeomphala Möllendorff, 1899: 96, pl. 6, fig. 3; – Wiegmann 1900: 111, pl. 3, figs 101–103; – Gude 1902: 6; – Yen 1939: 149, pl. 15, fig. 37; – Chen and Zhang 2004: 325, fig. 314; – Páll-Gergely et al. 2022: 67, fig. 18C.
Laeocathaica (Laeocathaica) phaeomphala – Zilch 1968: 174; – Richardson 1983: 78.

###### Museum material.

SMF 9089, lectotype; Wenhsien, S-Gansu; Potanin 51b, 72, 741, Slg. O. v. Möllendorff. SMF 9090, paratypes, four fms; the same data as lectotype. ZIN RAS No. 4, 1 fma and 1 subadult, Wen-Xian, 1885-IX-8, coll. Potanin, det. Möllendorff.

**Figure 22.**
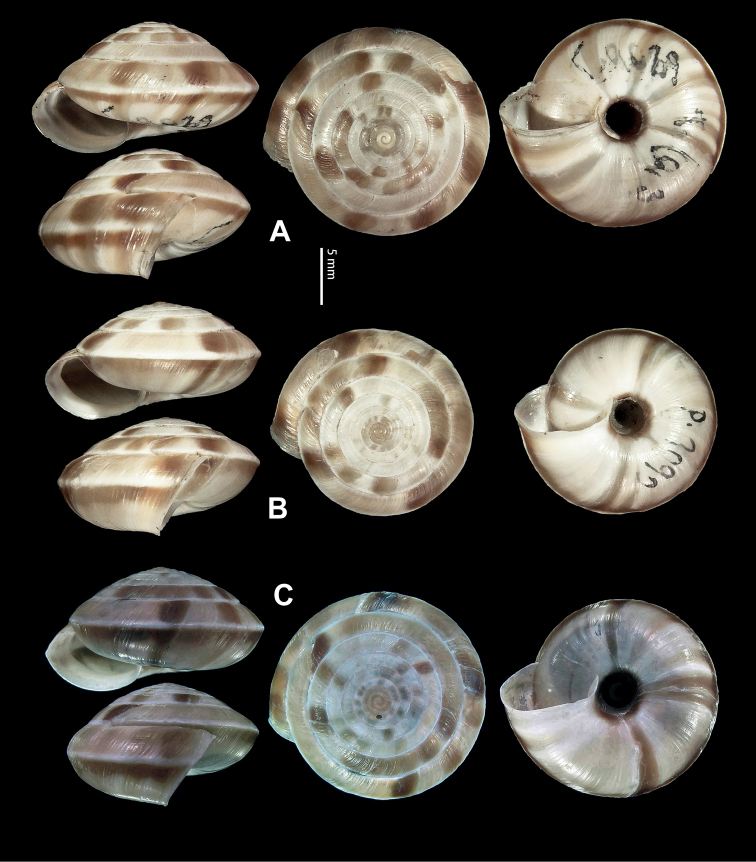
*Laeocathaicaphaeomphala* Möllendorff, 1899 **A**SMF 9089, lectotype **B**SMF 9090, paratype **C** HBUMM05433a-spec.6.

###### New material.

HBUMM05433, 2 fma, 1 fms, and many juvs, all fma dissected; Yuxushan, Wenxian, Gansu Province, 32.957259°N, 104.689152°E, shrubs and slate, 2006-IX-27, coll. Wu, M., Liu, J.-M., Zheng, W. and Gao, L.-H. HBUMM05477, numerous fma; Jidushan nearby Baishuijiang, Wenxian, Gansu Province; limestone and broad-leaved woods; 2006-IX-27; coll. Wu, M., Liu, J.-M., Zheng, W. and Gao, L.-H. HBUMM08424, slope near Wenzhoulu [文州路], Wenxian, Gansu Province, near 32.943151°N, 104.692505°E, on slope; 2019-X-12, coll. Li, Q.-M.; DNA voucher HBUMM08424a. CZG202008-w3, 9 subadults, Shangdezhen, Wenxian, Gansu Province, 2020-VIII, coll. Chen, Z.-G.

**Figure 23.**
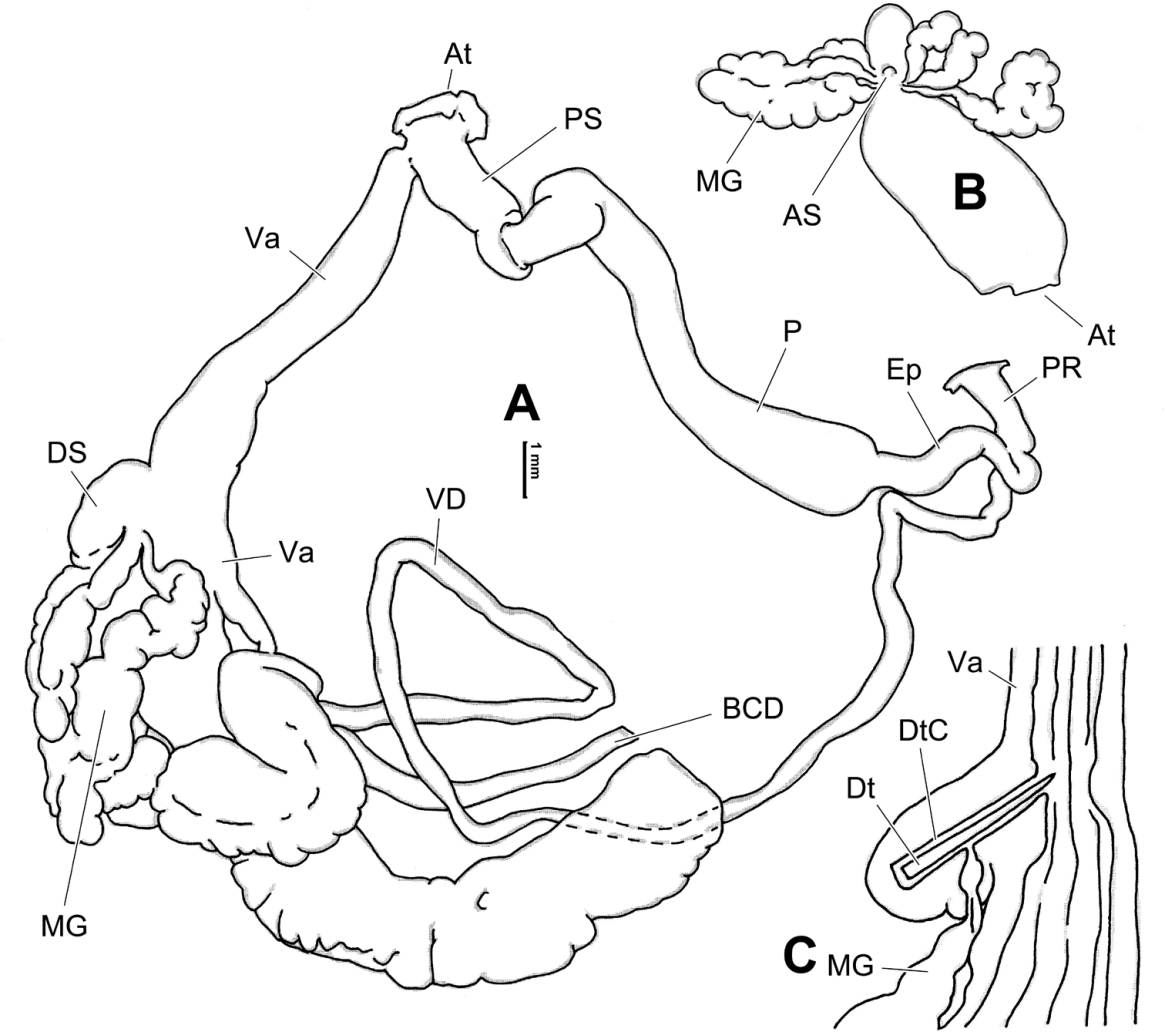
Genital anatomy of *Laeocathaicaphaeomphala* Möllendorff, 1899, HBUMM05433a-spec.6 **A** general view **B, C** ventral and left views of dart sac apparatus. Abbreviations: AS – accessory sac; At – atrium; BCD – bursa copulatrix duct; DS – dart sac; DtC – a chamber containing love dart; Ep – epiphallus; MG – mucous glands; P – penis; PR – penial retractor muscle; PS – penial sheath; Va – vagina; VD – vas deferens.

###### Distribution.

Gansu: Wenxian (type locality).

###### Additional information of shell.

The first 1^1^/_4_ protoconch whorls are almost smooth, and the subsequent 1/4 whorls have dense radially arranged fine threads. Spiral grooves are regularly present throughout body whorl.

###### General anatomy.

Head with flat but distinct eversible head wart. Jaw arcuate, with 5–7 projecting ribs.

###### Anatomy of genital organs.

Penial sheath covering ~ 1/5 of penis. Penis tubular and distally thicker. Inside penis, ~ 7 high parallel pilasters on proximal 1/2, no pilasters fusing into Y-shaped fork. Fine pilasters on distal end of penis merging into 9–12 thin or thick folds. Vas deferens narrow throughout. Vagina between atrium and dart sac extremely elongated, ~ 4× longer than dart sac. Vagina between dart sac and insertion of bursa copulatrix duct approximately as long as dart sac. Dart sac ~ 1/6 length of penis. Love dart ~ 1.5 mm long, apically 2-bladed. Accessory sac tiny, internally empty, inserting into dart sac at middle part, opening to dart chamber. Mucous glands eight, each singly tubular or simply branched. Proximal accessory sac absent. Bursa copulatrix duct equally thick.

###### Remarks.

This species is one of the three species that do not have proximal accessory sac on dart sac apparatus. However, the shell morphology of *Laeocathaicaphaeomphala* differs greatly from that of the other two species *L.polytyla* and *L.tropidorhaphe*.

##### ﻿ Laeocathaica polytyla

Taxon classificationAnimaliaStylommatophoraCamaenidae

Möllendorff, 1899

59689614-5308-5EFF-BB49-6202AE40C409

**Figure 24.**
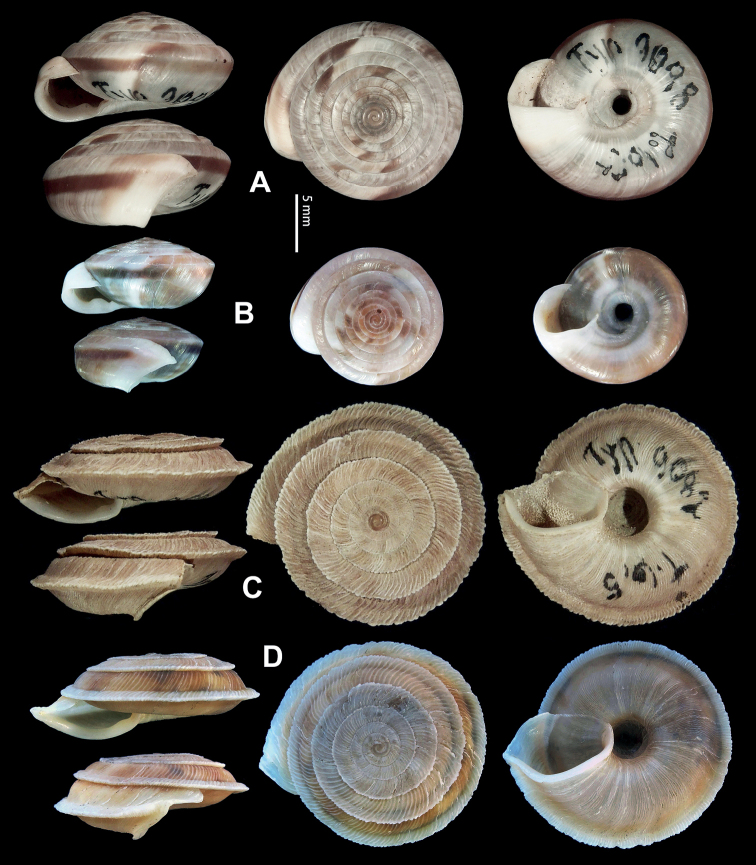
**A, B***Laeocathaicapolytyla* Möllendorff, 1899 **A**SMF 9098, lectotype **B** HBUMM05437-spec.1 **C, D***L.potanini* Möllendorff, 1899 **C**SMF 9082, paratype **D** HBUMM00633-spec.1.

Figs 2A , 3 , 24A, B , 26 , 43A, B , 45K, L , 51 ; Tables 1 , 3

Laeocathaica polytyla Möllendorff, 1899: 98, pl. 6, fig. 7; – Wiegmann 1900: 118, pl. 3, figs 105–107; – Gude 1902: 6; – Yen 1939: 150, pl. 15, fig. 41; – Schileyko 2004: 1686, fig. 2174B–D; – Chen and Zhang 2004: 331, fig. 322; – Páll-Gergely et al. 2022: 68, fig. 20A–C.
Laeocathaica (Laeocathaica) polytyla – Zilch 1968: 174; – Richardson 1983: 78.

###### Museum material.

SMF 9098, lectotype; Nanping, Sung-pan. SMF 9099, paratypes; six fms; Nanping, Sung-pan. Other SMF material see in Yen 1939 (150).

###### New material.

HBUMM00506, 13 fma, 1 dissected; Anchanghexiang [安昌河乡], Wenxian, Gansu Province, 1200 m, near point (33.066003°N, 104.460018°E); 1998-V-19, coll. Chen, D.-N. and Zhang, G.-Q. HBUMM00513, a subadult; Shuanghexiang [双河乡], Jiuzhaigouxian, Sichuan Province, 1100 m, near (33.179715°N, 104.264968°E); 1998-V-18, coll. Chen, D.-N. and Zhang, G.-Q. HBUMM00579, many fms; Jiuzhaigouxian, Sichuan Province; 1998-V-18, coll. Chen D. and Zhang, G.-Q. HBUMM05419, 1 fms; hill foot of Yuxushan, Wenxian, Gansu Province; slate and shrubs, dry; 2006-IX-27, coll. Wu, M., Liu, J.-M., Zheng, W. and Gao, L.-H. HBUMM06490, many fms; Yuxushan, Wenxian, Gansu Province, 1070 m a.s.l., 32.959861°N, 104.678222°E, shrubs and slate, 2011-VIII-07, coll. Wu, M., Xu, Q. and Budha, P. HBUMM06523, many fms and juvs; town of Wenxian, Gansu Province, 1024 m a.s.l., 32.941111°N, 104.668889°E; 2011-VIII-08, coll. Wu, M., Xu, Q. and Budha, P. HBUMM08454, 23 fms, east of the town of Jiuzhaigouxian, Sichuan Province, 2020-VIII, coll. Chen, Z.-G. CZG202008-w6, 1 subadult; Shangdezhen, Wenxian, Gansu Province, near point (32.907414°N, 104.76994°E); 2020-VIII, coll. Chen, Z.-G. CZG202008-w11, 2 fms, near point (32.94936°N, 104.693006°E), town of Wenxian, Gansu Province, 2020-VIII, coll. Chen, Z.-G. HBUMM05437, many fma; 1 fma dissected; Yuxushan, Wenxian, Gansu Province, 32.957259°N, 104.689152°E, shrubs and slate, 2006-IX-27, coll. Wu, M., Liu, J.-M., Zheng, W. and Gao, L.-H.; DNA voucher HBUMM05411. HBUMM06753, 3 fma, 7 fms; Eastern bank of Baishuijiang, Lihuacun, Jiuzhaigouxian, Sichuan Province, 1425 m, 33.267222°N, 104.234722°E; 2011-VI-14, coll. Wu, M., Xu, Q. and Buhda, P.

###### Distribution.

Gansu: Wenxian. Sichuan: Jiuzhaigouxian (type locality).

###### Additional information of shell.

Tiny elongate granules on protoconch, which can be only observed in juvenile shells, are densely and radially arranged. The granules on protoconch are absent in adult shell because of erosion or weathering. On teleoconch the growth lines are indistinct. Spiral grooves are absent throughout.

**Figure 25.**
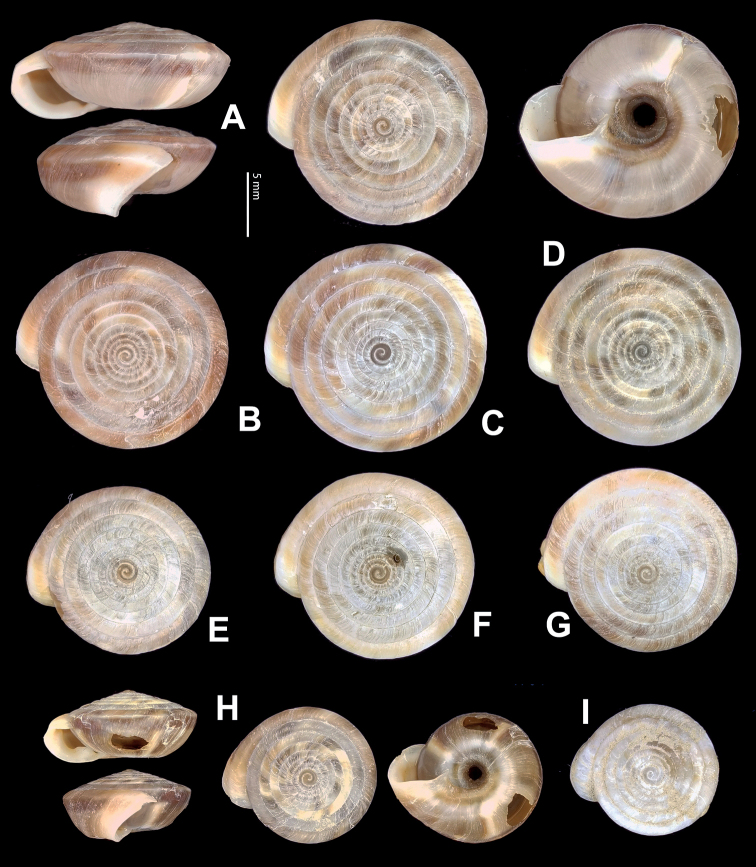
*Laeocathaicaparapolytyla* Wu, sp. nov. **A–E** paratypes HBUMM06640-spec.2–6 **F** holotype, HBUMM06640-spec.1 **G–I** paratypes HBUMM06640-spec.7–9. Both **A, H** show four views **B–G, I** show apical view only.

###### General anatomy.

Eversible head wart prominent. Jaw arcuate, with three projecting ribs.

###### Anatomy of genital organs.

Penial sheath covering ~ 1/4 of penis. Penis proximately tubular and distally expanded. Inside penis, two pilasters fusing into one Y-shaped fork at proximal 1/5, two more pilasters of similar thickness also present. Fine pilasters on distal end of penis merging into ~ 8 thick folds. Vas deferens narrow throughout. Vagina between atrium and dart sac not elongated. Vagina between dart sac and insertion of bursa copulatrix duct ~ 1/2 length of dart sac. Dart sac ~ 1/2 length of penis. Love dart ~ 1 mm long, apically 2-bladed. Accessory sac small but externally distinguishable, internally solid, inserting into dart sac at distal 1/3, opening to dart sac chamber. Mucous glands four or five, each singly tubular or simply branched. Proximal accessory sac absent. Bursa copulatrix duct equally thick.

**Figure 26.**
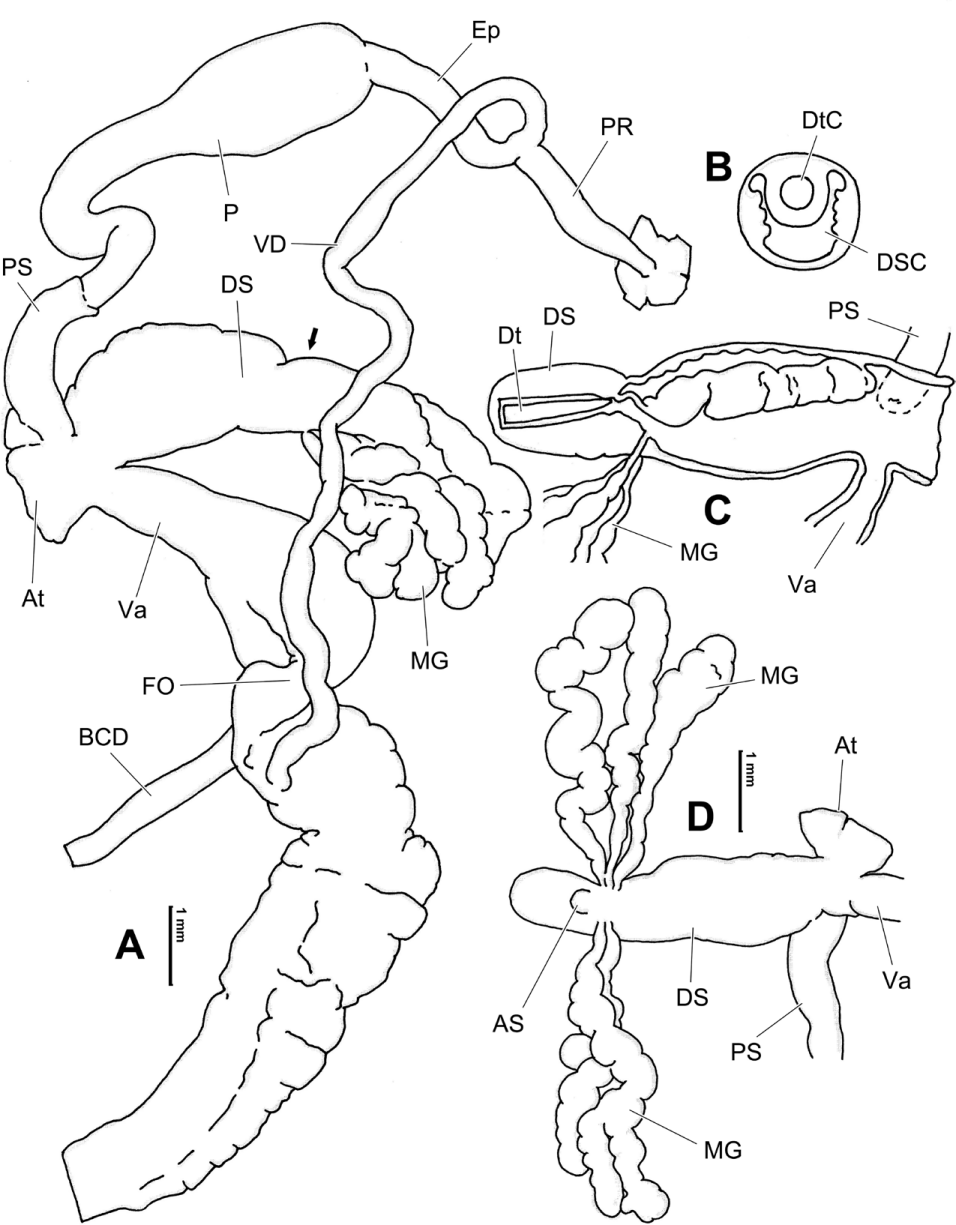
Genital anatomy of *Laeocathaicapolytyla* Möllendorff, 1899, HBUMM05437-spec.1 **A** general view **B** cross-section of dart sac at the position arrowed in (**A**) **C, D** left and ventral side of dart sac apparatus. Abbreviations: AS – accessory sac; At – atrium; BCD – bursa copulatrix duct; DS – dart sac; DSC – dart sac chamber; DtC – a chamber containing love dart; Ep – epiphallus; FO – free oviduct; MG – mucous glands; P – penis; PR – penial retractor muscle; PS – penial sheath; Va – vagina; VD – vas deferens.

###### Remarks.

See *Laeocathaicaparapolytyla* Wu, sp. nov.

##### ﻿ Laeocathaica potanini

Taxon classificationAnimaliaStylommatophoraCamaenidae

Möllendorff, 1899

CD8C9B6D-B1F8-5FE7-8672-6144EFB3162D

Figs 2A , 3 , 24C, D , 28 , 43C , 45A, B , 51 ; Tables 1 , 3

Laeocathaica potanini Möllendorff, 1899: 96, pl. 6, fig. 5; – Wiegmann 1900: 109, pl. 3, figs 98–100; – Gude 1902: 6; – Yen 1939: 149, pl. 15, fig. 38; – Yen 1942: 284; – Chen and Zhang 2004: 326, fig. 316; – Páll-Gergely et al. 2022: 70, fig. 23A, B.
Laeocathaica (Laeocathaica) potanini – Zilch 1968: 174; – Richardson 1983: 79.

###### Museum material.

SMF 9082, lectotype; Wenhsien, S-Gansu; Potanin 251, 587, 734, Slg. O. v. Möllendorff. SMF 9083, paratypes, three fms; Same data as lectotype. SMF 8960, paratype; Hungdan (= Hengdan) b. Wen-hsien, S-Gansu; ex Beresowski, Slg. O. v. Möllendorff.

###### New material.

HBUMM00633, many fma; 4 fma dissected; Yuxushan, Wenxian, Gansu Province, 1000 m a.s.l., near 32.957259°N, 104.689152°E, shrubs and slate, 1998-V-17, coll. Chen, D.-N. and Zhang, G.-Q. HBUMM00700, 5 fma and 2 juvs; Hejiaping [何家坪], Wenxian, Gansu Province, near 32.844062°N, 105.021857°E; 1998-IV-24, coll. Chen, D.-N. and Zhang, G.-Q. HBUMM05423, 4 juvs; hill foot of Yuxushan, Wenxian, Gansu Province; slate and shrubs, dry; 2006-IX-27, coll. Wu, M., Liu, J.-M., Zheng, W. and Gao, L.-H. HBUMM05427, many fma and fms; DNA voucher HBUMM05409. HBUMM05438, 4 fma, 7 fms, 7 juvs: Yuxushan, Wenxian, Gansu Province, 32.957259°N, 104.689152°E, shrubs and slate, 2006- IX-27, coll. Wu, M., Liu, J.-M., Zheng, W. and Gao, L.-H. HBUMM06483, Yuxushan, Wenxian, Gansu Province, 1070 m a.s.l., 32.959861°N, 104.678222°E; shrubs and slate; 2011-VIII-07, coll. Wu, M., Xu, Q. and Budha, P. (not dissected); DNA voucher HBUMM06482. HBUMM06520, 3 fms and 2 juvs; town of Wenxian, Gansu Province, 1024 m a.s.l., 32.941111°N, 104.668889°E; 2011-VIII-08, coll. Wu, M., Xu, Q. and Budha, P. HBUMM08427, slope near X496, Wenxian, Gansu Province, near 32.474345°N, 104.915976°E, on slope; 2019-X-13, coll. Li, Q.-M. (not dissected); DNA voucher HBUMM08427a. CZG202008-w1, 7 fms and many juvs, town of Wenxian, Gansu Province, 2020-VIII, coll. Chen, Z.-G.

**Figure 27.**
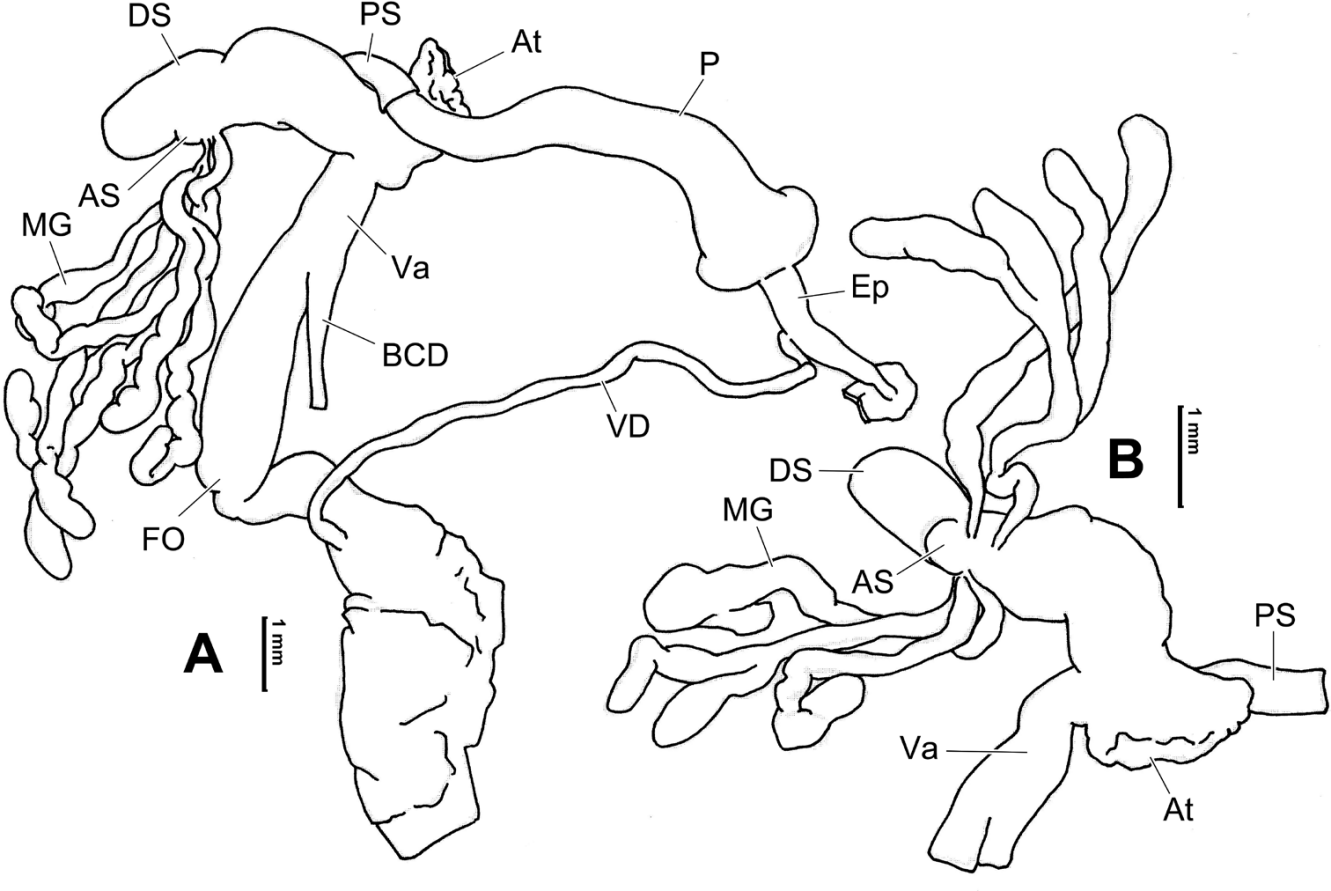
Genital anatomy of *Laeocathaicaparapolytyla* Wu, sp. nov., HBUMM06640-spec.1, holotype **A** general view **B** ventral view of dart sac apparatus. Abbreviations: AS – accessory sac; At – atrium; BCD – bursa copulatrix duct; DS – dart sac; Ep – epiphallus; FO – free oviduct; MG – mucous glands; P – penis; PR – penial retractor muscle; PS – penial sheath; Va – vagina; VD – vas deferens.

###### Distribution.

Gansu: Wenxian (type locality).

###### Additional information of shell.

Granules on protoconch can only be unclearly and partially observed near suture because of erosion or weathering in adults. Granules are short (~ 20 µm long) on fine threads. Spiral grooves are absent throughout. On teleoconch whorls, fine threads are present between every two adjacent ribs.

**Figure 28.**
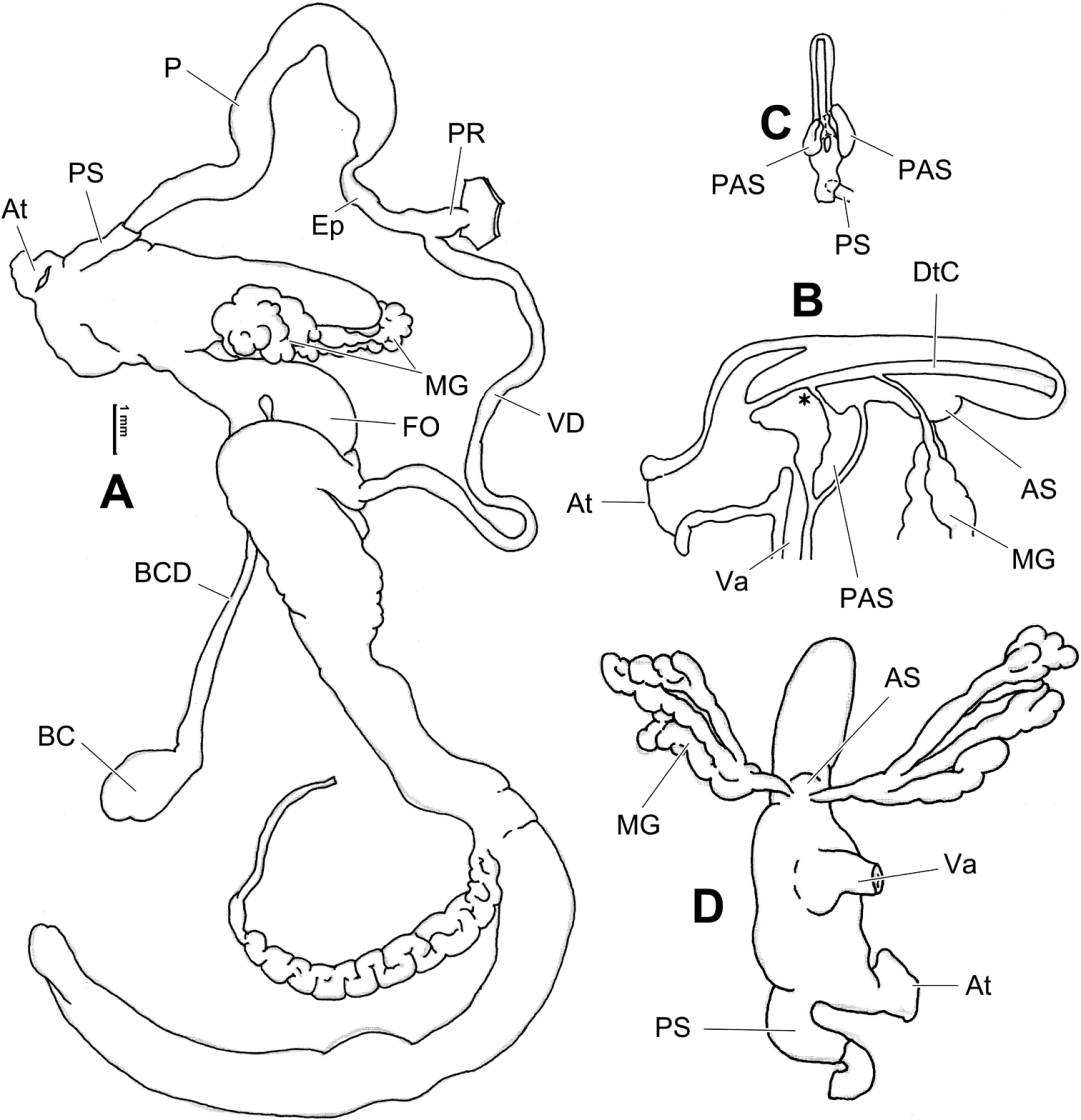
Genital anatomy of *Laeocathaicapotanini* Möllendorff, 1899, HBUMM00633-spec.1 **A** general view **B–D** right, apical, and ventral views of dart sac apparatus. Abbreviations: AS – accessory sac; At – atrium; BC – bursa copulatrix; BCD – bursa copulatrix duct; DS – dart sac; DtC – a chamber containing love dart; Ep – epiphallus; FO – free oviduct; MG – mucous glands; P – penis; PAS – proximal accessory sac; PR – penial retractor muscle; PS – penial sheath; Va – vagina; VD – vas deferens. Asterisk * indicates the opening of proximal accessory sac.

###### General anatomy.

Eversible head wart present. Jaw arcuate, with five projecting ribs.

###### Anatomy of genital organs.

Penial sheath short, covering ~ 1/6 of penis. Penis tubular, somewhat expanded distally. Inside penis, ~ 5 low pilasters present proximally, another two high pilasters fusing into one Y-shaped fork at proximal 1/2. Fine pilasters on distal end of penis merging into ~ 4 thick folds. Vas deferens narrow throughout. Vagina between atrium and dart sac not elongated. Vagina between dart sac and insertion of bursa copulatrix duct ~ 1/2 length of dart sac. Dart sac ~ 1/2 length of penis. Love dart ~ 6 mm long, apically rhombic in cross-section, subsequently 2-bladed. Accessory sac small but externally distinguishable, internally solid, inserting into dart sac at middle part, opening to dart chamber. Mucous glands two (*n* = 3) or three (*n* = 1), each complicatedly branched. Proximal accessory sacs two, symmetrical, apically separated and ventrally touching, each distally with an opening leading to proximal dart chamber. Bursa copulatrix duct basally thick. Bursa copulatrix ovate, small.

##### ﻿ Laeocathaica prionotropis

Taxon classificationAnimaliaStylommatophoraCamaenidae

Möllendorff, 1899

53E2C1D8-5164-5C8C-AAFF-5F2B8BA5389A

**Figure 29.**
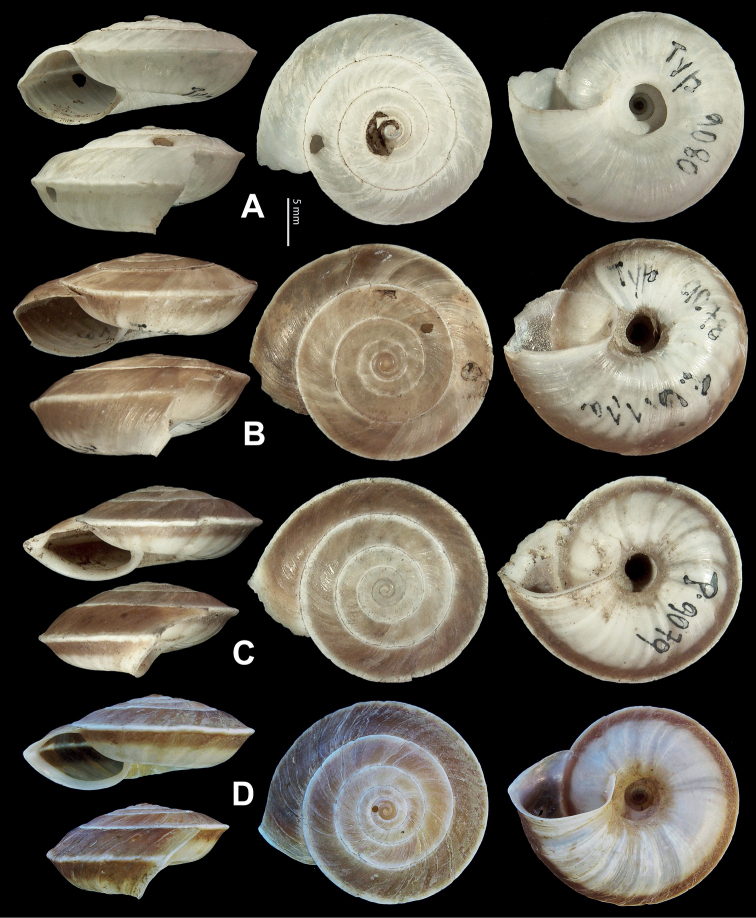
*Laeocathaicaprionotropis* Möllendorff, 1899 **A**SMF 9080, lectotype of *L.prionotropisalbocincta* Möllendorff, 1899 **B**SMF 9078, lectotype **C**SMF 9079, paratype **D** HBUMM05549-spec.1.

**Figure 30.**
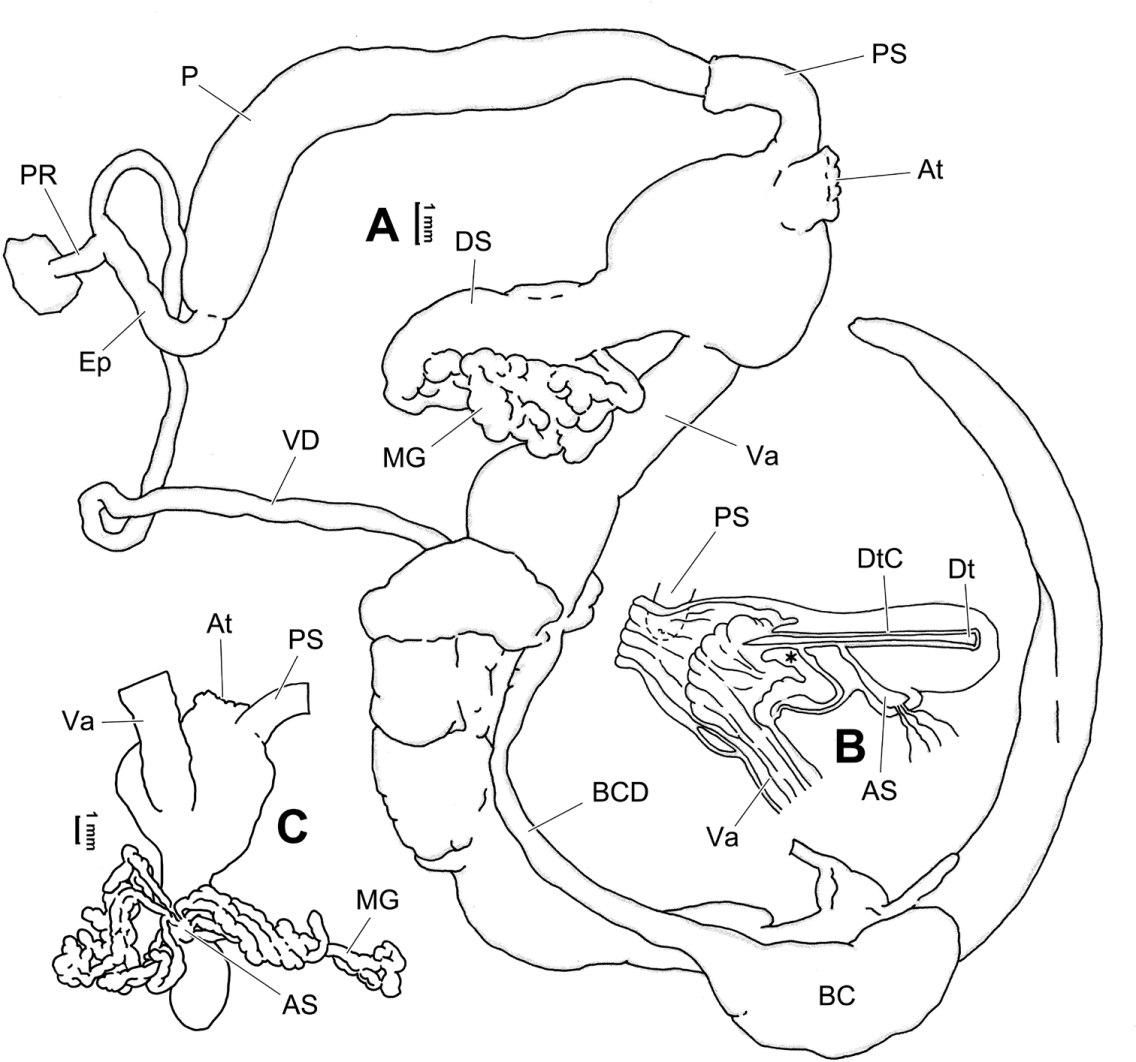
*Laeocathaicaprionotropis* Möllendorff, 1899, HBUMM05549-spec.1 **A** general view **B** right view of dart sac apparatus **C** ventral view of dart sac apparatus. Abbreviations: AS – accessory sac; At – atrium; BC – bursa copulatrix; BCD – bursa copulatrix duct; DS – dart sac; DtC – a chamber containing love dart; Ep – epiphallus; MG – mucous glands; P – penis; PR – penial retractor muscle; PS – penial sheath; Va – vagina; VD – vas deferens. Asterisk * indicates the opening of proximal accessory sac.

Figs 2A , 3 , 29 , 30 , 43D , 44E, F , 51 ; Tables 1 , 3

Laeocathaica prionotropis Möllendorff, 1899: 94, pl. 6, fig. 1, 1a; – Wiegmann 1900: 106, pl. 3, figs 94–97; – Gude 1902: 6; – Yen 1939: 149, pl. 15, fig. 34; – Chen and Zhang 2004: 320, fig. 309; – Páll-Gergely et al. 2022: 70, figs 25, 26.
Laeocathaica prionotropis albocincta Möllendorff, 1899: 95; – Gude 1902: 6; – Yen 1939: 149, pl. 15, fig. 35.
Cathaica prionotropis albocincta – Yen 1938: 446.
Laeocathaica (Laeocathaica) prionotropis – Zilch 1968: 174; – Richardson 1983: 79.
Laeocathaica (Laeocathaica) prionotropis albocincta – Zilch 1968: 175; – Richardson 1983: 79.

###### Museum material.

ZIN RAS No. 7, 2 subadults with soft parts, Wen-Xian, coll. Potanin, 1885-IX-6–8, det. Möllendorff. SMF 9078, lectotype. SMF 9079, 2 paratypes. *Laeocathaicaprionotropisalbocincta* Möllendorff, 1899: SMF 9080, lectotype; Tung-ho, W-Sy-tshuan; ex Potanin 312a, Slg. O. v. Möllendorff. SMF 9081, paratype, juv; ex Potanin 312b, Slg. O. v. Möllendorff.

###### New material.

HBUMM00422, 3 fms; Yanwuba [演武坝], Wenxian, Gansu Province, 1200 m a.s.l., 32.936494°N, 104.557695°E; 1998-IV-23, coll. Chen, D.-N. and Zhang, G.-Q. HBUMM00687, Shanggou [上垢], Shawanxiang [沙湾乡], Dangchangxian, Gansu Province, near 33.625712°N, 104.570162°E; 1998-V-6, coll. Chen, D.-N. and Zhang, G.-Q. HBUMM05165, 3 fma, 2 fms; Yuxushan, Wenxian, Gansu Province, near 32.957259°N, 104.689152°E, shrubs and slate, 1998-V-17, coll. Chen, D.-N. and Zhang, G.-Q. HBUMM05496, numerous fma and juvs, 1 dissected; 5 km away from Hejiawan [何家湾] Bridge, along the road to Bikou, Wenxian, Gansu Province, near point (32.769664°N, 105.22781°E), shrubs, limestones, loess, near farmland, 2006- IX-28, coll. Wu, M., Liu, J.-M., Zheng, W. and Gao, L.-H. HBUMM05549, many fma and juvs; Hengdan, Wenxian, Gansu Province; north side of Baishuijiang River, along 212 Guodao, near point (32.864025°N, 104.859517°E); hillside, bushes; 2006-IX-29; coll. Wu, M., Liu, J.-M., Zheng, W. and Gao, L.-H. HBUMM05559, many fma; HBUMM05777, 7 fma and 2 juvs, 1 dissected; same data as HBUMM05549. HBUMM05756, juvs; Zhouquxian [舟曲县], Gansu Province, near point (33.797295°N, 104.38151°E); limestone, broad-leaved trees and shrubs; 2006-X-5, coll. Zheng, W. and Liu, J.; 5742. HBUMM05564, many fma and juvs; Hengdan, Wenxian, Gansu Province; north side of Baishuijiang River, along 212 Guodao, near point (32.864025°N, 104.859517°E); top of hill, bushes; 2006-IX-29; coll. Wu, M., Liu, J.-M., Zheng, W. and Gao, L.-H. HBUMM05689, Guantingzhen, Dangchangxian, Gansu Province, near point (33.82428°N, 104.538282°E); limestone hills, along 212 Guodao; 2006-X-3, coll. Zheng, W. and Liu, J.-M. HBUMM05765, many fma and juvs; HBUMM05764b, 1 fma and 1 fms: Bikou, along the road from Datang Hydropower Station to Hejiawan Bridge; 2006-IX-28, coll. Liu, J.-M. and Zheng, W. HBUMM05766, many fma; same data as HBUMM05765. HBUMM05774, 5 fma, 4 juvs; same data as HBUMM05765. HBUMM06533, 1 fma; town of Wenxian, Gansu Province, 1024 m a.s.l., 32.941111°N, 104.668889°E, 2011-VIII-08, coll. Wu, M., Xu, Q. and Budha, P.; DNA voucher HBUMM06534. HBUMM08120, many juvs; Zhuantangzhen [篆塘镇], 2 juv shells, Chongqing, near point (28.898641°N, 106.675179°E); 2014-X-10, coll. Du, Li [杜莉] and Lai, Yitong [来益同]. HBUMM08150, slope near river, Wenxian, Gansu Province, 807 m, 32.847111°N, 104.88775°E; grass and a few shrubs, on branches and rock cliff; 2017-VIII-6, coll. Sheng, X.-F., etc.; DNA voucher HBUMM08150a. HBUMM08299, 5 fma and 1 juv, 1 dissected; Bikou, Wenxian, Gansu Province; 2019-IV, coll. Li, Q.-S. HBUMM05549, many fma; 1 fma dissected; Hengdan, along national road 212, Wenxian, Gansu Province, near 32.855948°N, 104.865229°E, shrubs on hill slope, 2006-IX-29, coll. Wu, M., Liu, J.-M., Zheng, W. and Gao, L.-H. HBUMM05469, many fma; 1 fma dissected; bank of Baishuijiang, Wenxian, Gansu Province, 32.946383°N, 104.685343°E, limestone and loess, broad-leaved woods, 2006-IX-27, coll. Wu, M., Liu, J.-M., Zheng, W. and Gao, L.-H.; DNA voucher HBUMM05440. HBUMM08366, 1 fma dissected; Gansu Province, 2019, coll. Li, Q.-S.; DNA voucher HBUMM08366a. HBUMM08423, 1 fma dissected; slope near Wenzhoulu, Wenxian, Gansu Province, near 32.943151°N, 104.692505°E, on slope, 2019-X-12, coll. Li, Q.-M.; DNA voucher HBUMM08423a. HBUMM08421, 1 fma dissected; slope near X496, Wenxian, Gansu Province, near 32.474345°N, 104.915976°E, on slope, 2019-X-13, coll. Li, Q.-M.; DNA voucher HBUMM08421a. CZG202008-w4, 2 fms, Yuleixiang [玉垒乡], Wenxian, Gansu Province, near point (32.83335°N, 105.036382°E); 2020-VIII, coll. Chen, Z.-G. CZG202008-w7, 1 subadult, town of Jiuzhaigouxian, Sichuan Province, near point (33.260369°N, 104.248974°E); 2020-VIII, coll. Chen, Z.-G. CZG202008-w8, 1 fms, roadside, from Longnan to Wenxian, Gansu Province, 2020-VIII, coll. Chen, Z.-G.

###### Distribution.

Chongqing. Gansu: Dangchangxian, Wenxian (type locality), Zhouquxian. Sichuan: Jiuzhaigouxian.

###### Additional information of shell.

Tiny granules (~ 25 – ~ 40 µm) on the protoconch are regularly and densely arranged. Each granule is like a hump deposited in a shallow socket. On adult shell the protoconch granules are not clear, perhaps because of erosion. On teleoconch, tiny ear-shaped scales are arranged along growth lines. On umbilical side, scale scars are regularly arranged. Spiral grooves are only present on apical side of body whorl. The shell of specimens HBUMM08366 and HBUMM08421 are not typical because the periphery is angular rather than sharply carinate.

###### General anatomy.

Eversible head wart of normal size. Jaw arcuate, with five projecting ribs (HBUMM05469).

###### Anatomy of genital organs.

Penial sheath short, proximally covering ~ 1/5 penis. Penis tubular, slightly expanded distally. Inside penis, three or four low pilasters present proximally, two fusing into one Y-shaped fork at proximal 1/2, another two high pilasters fusing into one Y-shaped fork at proximal 1/3 with cross expanded to be a large lump (~ 1 × 1 × 2 mm^3^). Fine pilasters on distal end of penis merging into more than eight thick but short folds. Vas deferens narrow throughout. Vagina between atrium and dart sac not elongated. Vagina between dart sac and insertion of bursa copulatrix duct ~ 1/3–1 length of dart sac. Dart sac ~ 1/2 (HBUMM05469) to 2/3 (HBUMM05549) length of penis. Love dart ~ 8 mm long, apically rhombic in cross-section. Accessory sac small but externally distinguishable, internally solid, inserting into dart sac at distal 1/3, opening to dart chamber. Mucous glands three (HBUMM08423), four (HBUMM05469, HBUMM08421), or eight (HBUMM05549), each simply or complicatedly branched. Proximal accessory sacs two, symmetrical, apically separated and ventrally touching, internally with a few low pilasters, each with a distal opening leading to proximal dart chamber. Bursa copulatrix duct of even diameter. Bursa copulatrix ovate, small.

###### Remarks.

The most distinctive character of the genitalia of *Laeocathaicaprionotropis* is the lump formed by the fusing pilasters in the penis.

The undetermined juvenile specimens from Chongqing (HBUMM08120) conchologically resemble *Laeocathaicaprionotropis*. However, their locality is far from the distribution area of *L.prionotropis* on the South Gansu Plateau [甘南高原].

##### ﻿ Laeocathaica stenochone

Taxon classificationAnimaliaStylommatophoraCamaenidae

Möllendorff, 1899

2563D1EB-7966-572D-8B60-C1C12871CE5E

**Figure 31.**
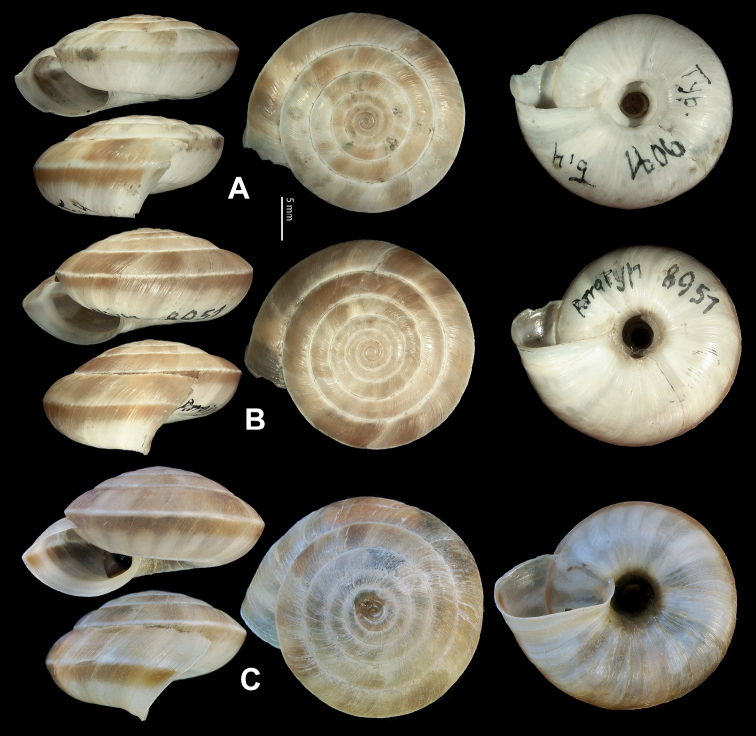
*Laeocathaicastenochone* Möllendorff, 1899 **A**SMF 9071, lectotype **B**SMF 8951, paratype **C** HBUMM05495-spec.1.

**Figure 32.**
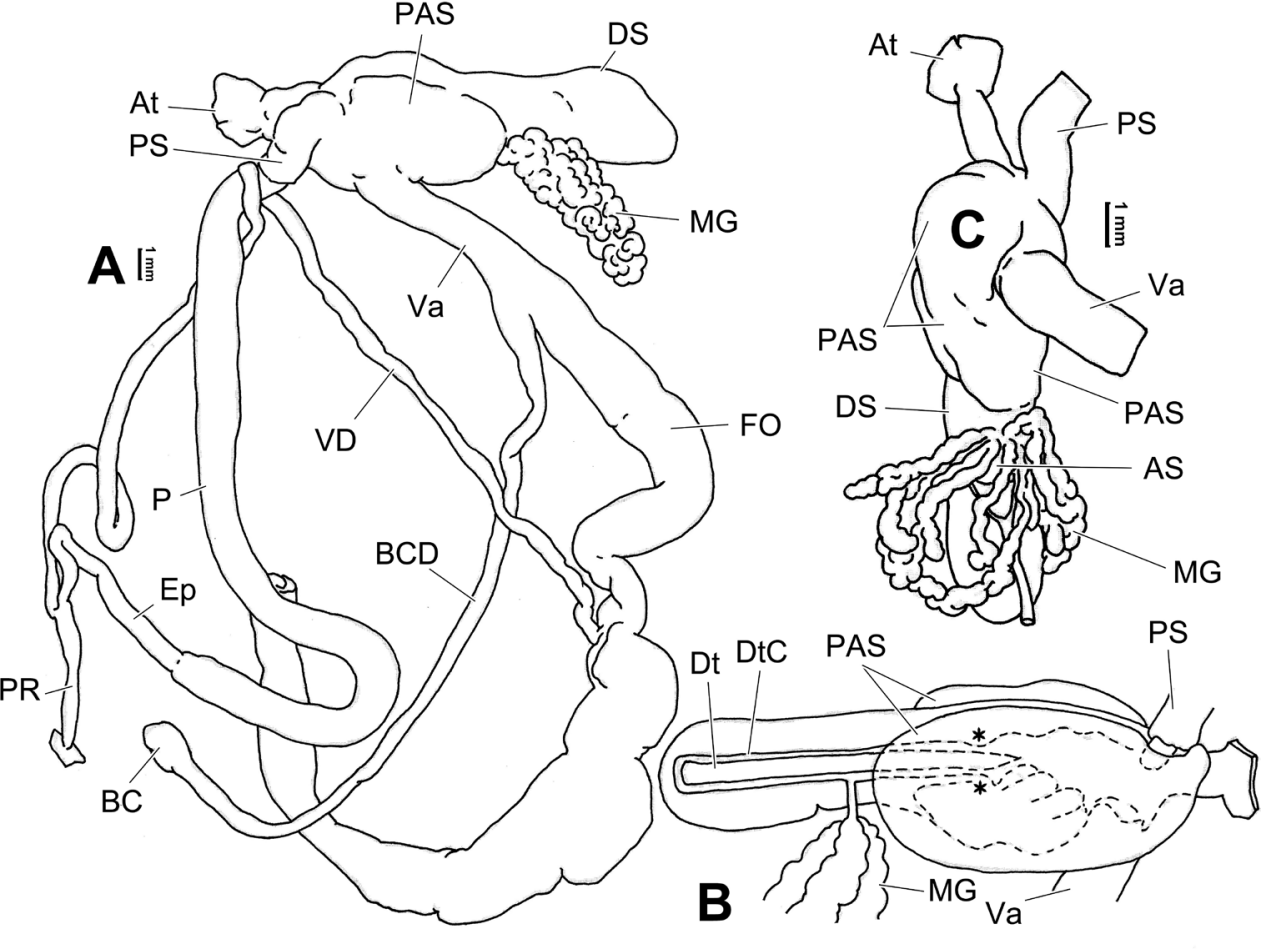
Genital anatomy of *Laeocathaicastenochone* Möllendorff, 1899, HBUMM05495-spec.1 **A** general view **B** left view of dart sac apparatus **C** ventral view of dart apparatus. Abbreviations: AS – accessory sac; At – atrium; BC – bursa copulatrix; BCD – bursa copulatrix duct; DS – dart sac; Dt – love dart; DtC – a chamber containing love dart; Ep – epiphallus; FO – free oviduct; MG – mucous glands; P – penis; PAS – proximal accessory sac; PR – penial retractor muscle; PS – penial sheath; Va – vagina; VD – vas deferens. Asterisk * indicates the opening of proximal accessory sac.

Figs 2A, C , 31 , 32 , 43E, F , 46C, D , 50B , 51 ; Tables 1 , 3

Laeocathaica stenochone Möllendorff, 1899: 91, pl. 5, fig. 4; – Wiegmann 1900: 100, pl. 3, figs 89,90; – Gude 1902: 6; – Yen 1939: 148, pl. 15, fig. 30; – Yen 1942: 283; – Chen and Zhang 2004: 314, fig. 301.
Laeocathaica subsimilis – Sturany 1900: 21 (Möllendorff 1901: 302).
Laeocathaica (Laeocathaica) stenochone – Zilch 1968: 175; – Richardson 1983: 79.
Laeocathaica carinifera – Páll-Gergely et al. 2022: 50, fig. 12D.

###### Museum material.

ZIN RAS No. 5, *Laeocathaicastenochone* Möllendorff., 1 fully mature soft part and 1 subadult, Zwischen dem Dorf Yu-Lin-guan und der Stadt Wen-hsien, 1885-IX-6–8, coll. Potanin, det. Möllendorff. SMF 9071, lectotype; Hsi-gu-tsheng (= town of Zhouquxian) [西固城], SO-Gansu, China; ex Potanin 577, Slg. O. v. Möllendorff. SMF 8951, paratype; Zw. Yue-ling-guan u. Wen-hsien; Slg. O. v. Möllendorff, ex potanin 730. SMF 9072, paratype, A shell with immature aperture; same data as lectotype. SMF 24270, paratype, not full matured at aperture; ?Sy-tshuan; ex Beresowski 908c, Slg. O. v. Möllendorff.

###### New material.

HBUMM05495, many fma; 1 fma dissected; 5 km away from Hejiawan Bridge, along the road to Bikou, Wenxian, Gansu Province, near point (32.769664°N, 105.22781°E), shrubs, limestones, loess, near farmland, 2006-IX-28, coll. Wu, M., Liu, J.-M., Zheng, W. and Gao, L.-H.; DNA voucher HBUMM05492. HBUMM05764, HBUMM05764a, HBUMM05767, HBUMM05769, HBUMM05772, HBUMM05774c: many fma and juvs; Bikou, along the road from Datang Hydropower Station to Hejiawan Bridge; 2006-IX-28, coll. Liu, J.-M. and Zheng, W. HBUMM08431, 1 fma dissected; near Shichuanba [石川坝], Wenxian, Gansu Province, near 33.17534°N, 105.019362°E; 2019-X-12, coll. Li, Q.-M.; DNA voucher HBUMM08431a.

###### Distribution.

Gansu: Huixian [徽县], Wenxian, Wudu, Zhouqu (type locality).

###### Additional information of shell.

The protoconch is radially and regularly covered with dense thick granules (~ 25 – ~ 55 µm long) on the first 1^1^/_4_ whorls. On the remaining protoconch whorls granules are indistinct and replaced by crowded radial threads. Spiral grooves are only indistinctly present on the apical side of body whorl.

###### General anatomy.

Eversible head wart indistinct. Jaw arcuate, with three projecting ribs.

###### Anatomy of genital organs.

Penial sheath very short, covering ~ 1/10 of penis. Penis tubular, equally thick. Inside penis, two high pilasters forming one Y-shaped fork at proximal 1/5, besides with two pilasters parallelly merging into one thickest pilaster. Fine pilasters on distal end of penis merging into ~ 6 thick folds, among which one is thicker than the others. Vas deferens narrow throughout. Vagina between atrium and dart sac not elongated. Vagina between dart sac and insertion of bursa copulatrix duct ~ 1/2 length of dart sac. Dart sac ~ 1/2 length of penis. Love dart ~ 9 mm long, apically 2-bladed, subsequently rounded. Accessory sac small, internally solid, inserting into dart sac at middle part, opening to dart chamber. Mucous glands ~ 5, each complicatedly branched. Proximal accessory sacs two, symmetrical, separated apically and touching ventrally, each with an opening leading to proximal dart chamber near dart chamber opening. Bursa copulatrix duct of even diameter. Bursa copulatrix ovate, very small.

###### Remarks.

Geographically, the distribution of *Laeocathaicacarinifera* in the Yangtze valley in Chongqing and Sichuan does not overlap those of *L.stenochone* and *L.qingchuanensis* Wu, sp. nov., both of which occur along the Bailongjiang River (Fig. 2C). In terms of shell morphology, *Laeocathaicastenochone* close to *L.carinifera* and *L.qingchuanensis* Wu, sp. nov. can be distinguished (Fig. 50B). *Laeocathaicastenochone* shows a particular protoconch and teleoconch sculpture (Fig. 46C, D), which is completely different from those of *L.carinifera* (Fig. 45I, J). In molecular analysis based on present combination of 16S + ITS2 sequences (Fig. 51), *Laeocathaicastenochone* (voucher HBUMM08431a) cannot be distinguished from *L.carinifera* (voucher HBUMM05103, HBUMM05131) because they share the same ITS2 sequence and show only 3-site difference in 16S sequence, suggesting they are possibly genetically very close species. However, *L.stenochone* and *L.carinifera* cannot be treated as one species (see Discussion).

Compared to *Laeocathaicacarinifera*, *L.stenochone* has a symmetrical dart sac apparatus, short penial sheath, and significantly longer and evenly slender penis (Fig. 32), in which the Y-shaped fork is present more proximally (Fig. 43E, F).

The specimens HBUMM08433 (8 fma, Wufengxi [五凤溪], Jintang County [金堂县], Chengdu [成都], Sichuan Province; coll. Li, Q.-M., 2019-XI; DNA voucher HBUMM08433a) that show a very similar shell to *Laeocathaicastenochone* could represent a different species to *L.carinifera* and *L.stenochone* due to a different genital trait.

##### ﻿ Laeocathaica tropidorhaphe

Taxon classificationAnimaliaStylommatophoraCamaenidae

Möllendorff, 1899

ABDDE866-1BE9-58A5-96D8-F8618DD6D3E2

**Figure 33.**
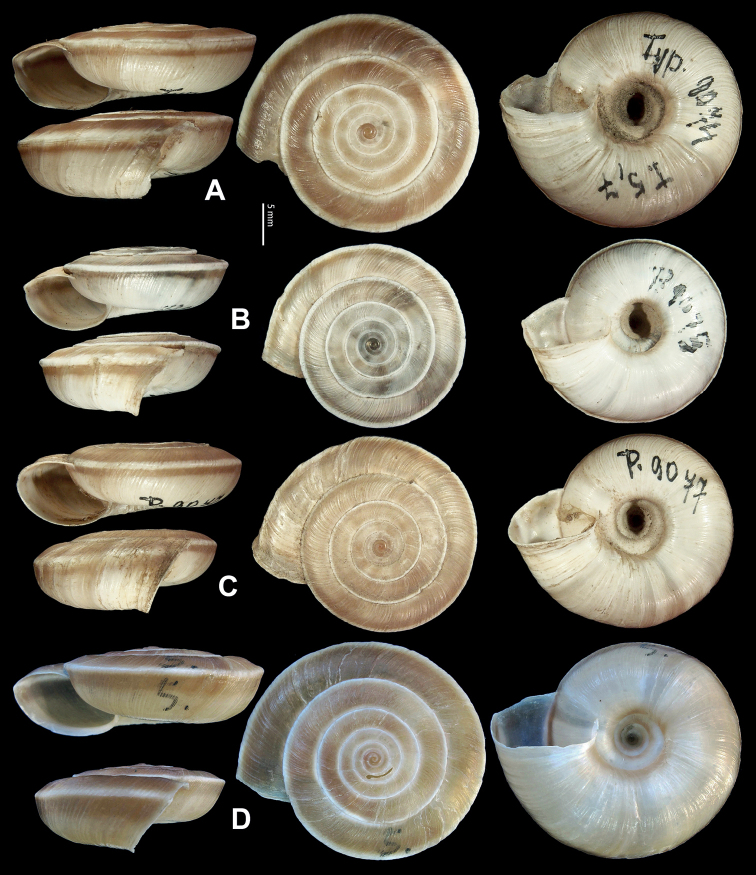
*Laeocathaicatropidorhaphe* Möllendorff, 1899 **A**SMF 9074, lectotype **B**SMF 9075, paratype **C**SMF 9077, paratype **D** HBUMM05664.

**Figure 34.**
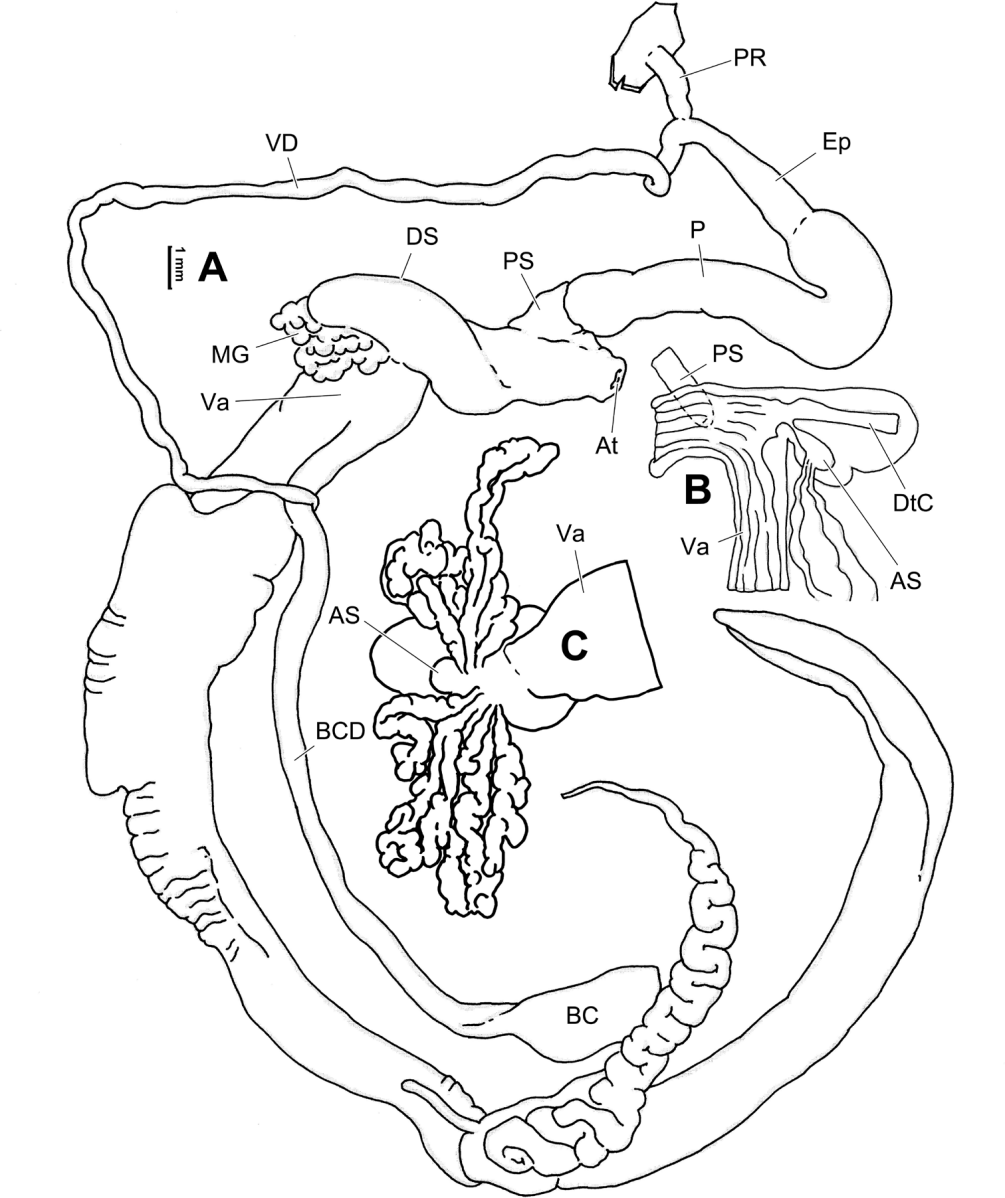
Genital anatomy of *Laeocathaicatropidorhaphe* Möllendorff, 1899 **A** general view **B** right view of dart sac apparatus, HBUMM05664-spec.1 **C** ventral view of dart apparatus **A, C** HBUMM05619-spec.1. Abbreviations: AS – accessory sac; At – atrium; BC – bursa copulatrix; BCD – bursa copulatrix duct; DS – dart sac; DtC – a chamber containing love dart; Ep – epiphallus; MG – mucous glands; P – penis; PR – penial retractor muscle; PS – penial sheath; Va – vagina; VD – vas deferens.

Figs 2A, D , 33 , 34 , 44A, B , 46I, J , 50A , 51 ; Tables 1 , 3

Laeocathaica tropidorhaphe Möllendorff, 1899: 94, pl. 5, fig. 7; – Gude 1902: 6; – Yen 1939: 149, pl. 15, fig. 33; – Yen 1942: 284; – Chen and Zhang 2004: 319, fig. 307.
Laeocathaica (Laeocathaica) tropidorhaphe – Zilch 1968: 175; – Richardson 1983: 79.
Laeocathaica dangchangensis Chen & Zhang, 2004: 339, 443, fig. 332.
Laeocathaica amdoana – Páll-Gergely et al. 2022: 38, fig. 7A, B, D.

###### Museum material.

SMF 9074, lectotype; Zw. Li-dshia-pu u. Hsi-gu-tsheng, S. O. Gansu; ex Potanin 923, Slg. O. v. Möllendorff. SMF 9075, paratypes, four fms; Tan-tshang (= Dangchang), SO-Gansu; ex Potanin 545, 623, 808b. SMF 9076, paratype, one fms; Dshie-dshou; ex Potanin 119, Slg. O. v. Möllendorff. SMF 9077, paratypes, two fms; the same data as lectotype. SMF 95126, paratype, one fms; SO-Gansu, NW-China; Slg. C. R. Böttger 1904 (ex Möllendorff!). SMF 24269, one fms labeled with “*L.stenochone*”; Tan-tschan; Slg. O. v. Möllendorff.

###### New material.

HBUMM00486, 3 fma; Daigusicun, Diebuxian, Gansu Province, 2000 m a.s.l., near 34.004644°N, 103.939651°E; 1998-V-10, coll. Chen, D.-N. and Zhang, G.-Q. HBUMM00519, 2 fma, 1 fms; Jinpingxiang [锦屏乡], Wudu, Gansu Province, 33.411281°N, 104.793036°E; 1998-V-11, coll. Chen, D.-N. and Zhang, G.-Q. HBUMM00526, 2 fma; Lijiexiang [立节乡], Zhouquxian, Gansu Province, 33.901549°N, 104.076052°E; 1998-V-10, coll. Chen, D.-N. and Zhang, G.-Q. HBUMM00527; 2 fma; Shanggou, Shawanxiang, Dangchangxian, Gansu Province, near 33.625712°N, 104.570162°E; 1998-V-6, coll. Chen, D.-N. and Zhang, G.-Q. HBUMM00693, 12 fma, 1 fma dissected: Wangguanxiang [望关乡], Kangxian [康县], Gansu Province, near point (33.461266°N, 105.378423°E); 1998-V-5, coll. Chen, D.-N. and Zhang, G.-Q. HBUMM05664, many fma, 1 fma dissected; east bank of Bailongjiang, Lianghekou, Dangchang, Gansu Province, near 33.695553°N, 104.496612°E, limestone, 2006-X-02, coll. Liu, J.-M. and Zheng, W. HBUMM05619, many fma, 1 fma dissected; Foyazhen [佛崖镇], Wudu, Gansu Province, 33.44°N, 105.27°E, limestone hill with thick shrubs and broad-leaved woods, 2006-X-02, coll. Liu, J.-M. and Zheng, W.; DNA voucher HBUMM05617. HBUMM06621, 2 fma; near Zhangzhazhen, Jiuzhaigouxian, Sichuan Province, 2100 m a.s.l., 33.299167°N, 103.859444°E; 2011-VIII-11, coll.Wu, M., Xu, Q. and Buhda, P.; DNA voucher HBUMM06620. HBUMM08425, 1 fma dissected; near Yangshanwan No. 2 Bridge, along the national road 212, Gansu Province, on slope, 2019-X-11, coll. Li, Q.-M.; DNA voucher HBUMM08425a. HBUMM05621, numerous fma, 1 fma dissected; Foyazhen, Wudu, Gansu Province, 33.44°N, 105.27°E, limestone hill with thick shrubs and broad-leaved woods, 2006-X-02, coll. Liu, J.-M. and Zheng, W. HBUMM05719, numerous fma, 1 fma dissected; Dangchang, Gansu Province, 2006-X-04, coll. Liu, J.-M. and Zheng, W.; DNA voucher HBUMM05716. HBUMM00450 (3 fma), HBUMM05664 (many fma, 1 fma dissected), Wangguanxiang, Kangxian, Gansu Province; 1998-V-1, coll. Chen, D.-N. and Zhang, G.-Q. HBUMM00508, 4 fma, 1 dissected; Xinglongcun, Zhongzhaixiang, Wenxian, Gansu Province; 1998-V-19, coll. Chen, D.-N. and Zhang, G.-Q. HBUMM00625, 1 fms and 2 shells of subadult, Erlangshan [二郎山], Zhouquxian, Gansu Province; 1998-V-9, coll. Chen, D.-N. and Zhang, G.-Q. HBUMM05166, 2 fma and 2 juvs; Zhongzhaixiang, Wenxian, Gansu Province; 1998-V-19, coll. Chen, D.-N. and Zhang, G.-Q. HBUMM05600, many fma and juvs; southern slope of Beishan, Wudu, Gansu Province, limestone hill with sparse shrubs; 2006-X-02, coll. Liu, J.-M. and Zheng, W. HBUMM05608, numerous fma; northern slope of Beishan, Wudu, Gansu Province, limestone hill with sparse shrubs; 2006-IX-IX-30, coll. Liu, J.-M. and Zheng, W. HBUMM05664, HBUMM05669, HBUMM05684 (many fma, 8 dissected): eastern bank of Bailongjiang, Lianghekou, Dangchangxian, Gansu Province, near point (33.697332°N, 104.493015°E); limestone; 2006-X-02, coll. Zheng, W. and Liu, J. HBUMM05691, HBUMM05692, HBUMM05693b, HBUMM05694: Guantingzhen, Dangchangxian, Gansu Province, near point (33.82428°N, 104.538282°E); limestone hills, along 212 Guodao; 2006-X-3, coll. Zheng, W. and Liu, J.-M.; DNA voucher HBUMM05688. HBUMM08369, 4 subadults; Gansu Province, 2019, coll. Li, Q.-S. CZG202008-w2, 4 subadults Shangdezhen, Wenxian, Gansu Province, near point (32.907414°N, 104.76994°E), 2020-VIII, coll. Chen, Z.-G. CZG202107-w1, 12 fms and 8 subadults, east of the town of Jiuzhaigouxian, Sichuan Province, near point (33.259523°N, 104.245884°E); 2021-VII, coll. Chen, Z.-G.

###### Distribution.

Gansu: Dangchangxian (type locality), Diebuxian, Kangxian, Wenxian, Wuduxian, Zhouquxian (type locality). Sichuan: Jiuzhaigouxian.

###### Additional information of shell.

Protoconch has regularly arranged fine granules (each ~ 30 µm long) and fine radial threads, both of which are usually obscured by erosion or weathering. Apical and umbilical sides of body whorl have densely and regularly arranged spiral grooves.

###### General anatomy.

Eversible head wart present. Jaw arcuate, with five projecting ribs.

###### Anatomy of genital organs.

Penial sheath covering ~ 1/6–1/5 of penis. Penis tubular, slightly thick distally. Inside penis, two adjacent pairs of pilasters forming two Y-shaped forks at proximal 1/4. Fine pilasters on distal end of penis merging into ~ 9 more or less thick short folds. Vas deferens narrow throughout. Vagina between atrium and dart sac elongated. Vagina between dart sac and insertion of bursa copulatrix duct ~ ^1^/_2_–1 length of dart sac. Dart sac ~ 1/3 length of penis. Love dart ~ 3 mm long, apically rhombic in cross-section. Accessory sac small, internally empty, inserting into dart sac proximally, opening to dart chamber. Mucous glands 7 (HBUMM05664) – 10 (HBUMM05619), each simply or complicatedly branched. Proximal accessory sacs absent. Bursa copulatrix duct of even diameter. Bursa copulatrix ovate, small.

###### Remarks.

In terms of shell shape, this species has an obviously depressed spire and more or less sharp carina or angulation above periphery compared to the sympatric (Fig. 2D) *Laeocathaicaamdoana* and *L.distinguenda*. Their shell differences are also indicated by the average shapes showed in Fig. 50A. The aperture of *Laeocathaicatropidorhaphe*, more or less stretched outwards, is narrower than that of *L.distinguenda* (Fig. 50A). *Laeocathaicadangchangensis* Chen & Zhang, 2004 shows a typical shell of *L.tropidorhaphe* (Fig. 50A).

The systematic affinity between *Laeocathaicaamdoana*, *L.distinguenda* and *L.tropidorhaphe* is supported by the phylogeny resulting from the present analyses (Fig. 51), which also indicates that the character state of the presence of proximal accessory sacs may have repeated on different branches of Clade M (*Laeocathaica*) (Fig. 51).

For more comments, see *Laeocathaicaamdoana*.

#### ﻿New taxa of *Laeocathaica*

##### ﻿ Laeocathaica parapolytyla

Taxon classificationAnimaliaStylommatophoraCamaenidae

Wu sp. nov.

4EB26675-F8C9-58C6-BCC1-DA0DCC8EA910

https://zoobank.org/C78DF85F-7C31-47A3-921D-BE2D72DF128A

Figs 2A, B , 25 , 27 , 42G, H , 48I, J ; Tables 1 , 2 [近多结射带蜗牛]

###### Type material.

***Holotype*** HBUMM06640-spec.1, 1 dissected; town of Wenxian, Gansu Province, 1269 m a.s.l., near point (32.944391°N, 104.685604°E); 2011-VIII-09, coll. Wu, M., Xu, Q. and Budha, P.; DNA voucher HBUMM06639. ***Paratypes*** HBUMM06640-spec.2–9 (apex of spec.9 was removed for SEM observation), same data as holotype. HBUMM00532, Xinglongcun, Zhongzhaixiang, Wenxian, Gansu Province, near point (33.232415°N, 104.419075°E); 1998-V-19, coll. Chen, D.-N. and Zhang, G.-Q.

###### Measurement of holotype.

Shell height 6.9 mm, maximum diameter 15.6 mm, aperture height 3.9 mm, aperture breadth 5.8 mm, umbilicus diameter 5.0 mm, protoconch whorls 1^1^/_2_, whorls 8^7^/_8_.

###### Diagnosis.

Protoconch with dense radially-arranged threads, visible through umbilicus. Beneath carina a clear chestnut band present. Mucous glands four or five. Penis with two pairs of pilasters fusing into two Y-shaped forks. Vagina between atrium and dart sac not elongated. Proximal accessory sac absent.

###### Description of shell.

Sinistral, depressed, thin but somewhat solid. Shell with 8^1^/_8_–9^3^/_8_ fairly flat whorls. Suture impressed. Protoconch 1^1^/_2_–1^3^/_4_ whorls, with densely arranged radial threads that may be invisible because of weathering or erosion. Periphery distinctly angulate. Growth lines indistinct. Spiral grooves are absent throughout. Aperture oblique, roundly square, descending in front. Peristome not expanded and indistinctly reflexed at lower part. Within aperture a ring-like thickening present, basally with a flat tooth. Columella oblique. Umbilicus abruptly broadened after penultimate whorl, ~ 1/4 of maximum diameter. Protoconch visible through umbilicus. Shell apically in intermittent yellowish white and pale chestnut patches except white carina. In umbilical view shell distinctly paler in greyish yellow, just beneath carina a clear chestnut band present, umbilical region brownish.

**Table 2.** Shell measurements (range, mean ± s.d.) of the new species of *Laeocathaica* Möllendorff, 1899 described in this work (length in mm).

Species	*n*	Shell height	Maximum diameter	Aperture height	Aperture breadth	Umbilicus diameter	Protoconch whorls	Whorls
*L.parapolytyla* Wu, sp. nov.	8	5.5–8.4 7.2±1.05	11.4–17.2 15.1±2.23	2.8–4.6 3.9±0.66	4.2–6.8 5.8±0.95	3.0–5.0 4.1±0.65	1.500–1.750 1.594±0.1108	8.125–9.375 8.875±0.4432
*L.qiminglii* Wu, sp. nov.	2	5.2–5.3 5.2±0.03	12.6–13.2 12.9±0.44	2.0–2.4 2.2±0.28	3.9–4.2 4.0±0.24	5.2–5.4 5.3±0.13	1.500	7.625–7.750 7.688±0.0883
*L.zhengpingliui* Wu, sp. nov.	10	7.9–10.0 9.2±0.77	17.2–20.1 19.0±1.1	4.6–6.8 5.8±0.61	5.9–8.1 7.2±0.67	6.0–7.9 6.7±0.56	1.500–1.750 1.600±0.0986	8.625–9.250 8.838±0.2361
*L.cheni* Wu, sp. nov.	9	8.6–10.4 9.3±0.56	16.5–19.7 18.7±1.05	5.0–6.5 6.0±0.48	6.0–8.1 7.3±0.62	4.8–7.1 6.2±0.72	1.500–1.625 1.609±0.0442	9.000–9.625 9.219±0.0.2086
*L.qingchuanensis* Wu, sp. nov.	6	10.7–13.9 12.3±1.19	20.7–27.5 24.5±2.10	6.2–8.5 7.6±0.78	9.0–11.9 10.7±0.99	4.1–6.4 5.1±0.59	1.500–1.625 1.510±0.0347	6.125–7.000 6.500±0.2447
*L.qishilii* Wu, sp. nov.	8	7.4–8.2 7.9±0.31	21.3–23.7 22.8±0.79	5.6–6.9 6.2±0.49	9.8–11.4 10.5±0.47	5.0–6.1 5.6±0.35	1.500–1.625 1.516±0.0442	5.500–5.875 5.703±0.1325
*L.nordsiecki* Wu, sp. nov.	5	3.6–3.8 3.7±0.08	10.3–10.8 10.5±0.18	2.2–3.0 2.7±0.31	2.9–3.3 3.1±0.16	4.6–5.0 4.8±0.13	1.500–1.625 1.600±0.0560	5.000–5.125 5.100±0.0559

###### General anatomy.

Eversible head wart prominent. Jaw arcuate, with four projecting ribs.

###### Anatomy of genital organs.

Penial sheath covering ~ 1/5 of penis. Penis distally fairly expanded. Inside penis, two high pilasters fusing into one Y-shaped fork at proximal 1/4, another pair of pilasters fusing into one Y-shaped fork at middle part. Fine pilasters on distal end of penis merging into ~ 7 thick and short folds. Epiphallic papilla absent. Vas deferens narrow throughout. Vagina between atrium and dart sac not elongated. Vagina between dart sac and insertion of bursa copulatrix duct ~ 1/2 length of dart sac. Dart sac ~ 1/2 length of penis. Accessory sac small but externally distinguishable, internally solid, ventrally inserting into dart sac at distal 1/3, together with mucous glands opening to dart chamber. Mucous glands four or five, each singly tubular or bifurcated. Proximal accessory sac absent. Bursa copulatrix duct equally thick.

###### Etymology.

The name of this new species is made up of *para*- meaning similar to and *polytyla* from *Laeocathaicapolytyla* Möllendorff, 1899, which is conchologically close to the new species.

###### Ecology.

On rocks of local hill.

###### Distribution.

Only known from the type locality.

###### Remarks.

The new species exhibits a large intraspecific change in size (Fig. 25), which is also showed in *Laeocathaicapolytyla* (Fig. 24A, B). *Laeocathaicaparapolytyla* Wu, sp. nov. looks like a flattened and sharply carinate *L.polytyla*, but has slightly different coloring and coarser growth lines. The terminal genitalia of these two species are similar, but in the new species the penis internally has two pairs of pilasters that fuse into two Y-shaped forks, while in *Laeocathaicapolytya* the penis has only one Y-shaped fork formed by adjacent pilasters.

##### ﻿ Laeocathaica qishilii

Taxon classificationAnimaliaStylommatophoraCamaenidae

Wu sp. nov.

30C7BD1A-D6A3-5ACC-99F3-9B276BA888CB

https://zoobank.org/DE713AA0-C437-4118-ABD7-97941F207D7D

**Figure 35.**
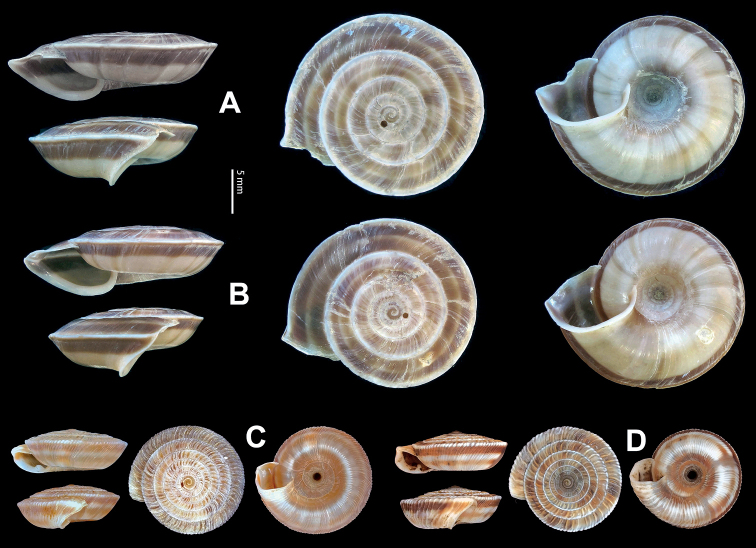
**A, B***Laeocathaicaqishilii* Wu, sp. nov. **A** holotype, HBUMM08298-spec.1 **B** paratype, HBUMM08298-spec.2 **C, D***L.qiminglii* Wu, sp. nov. **C** holotype, HBUMM08422-spec.1 **D** paratype, HBUMM08448-spec.1.

**Figure 36.**
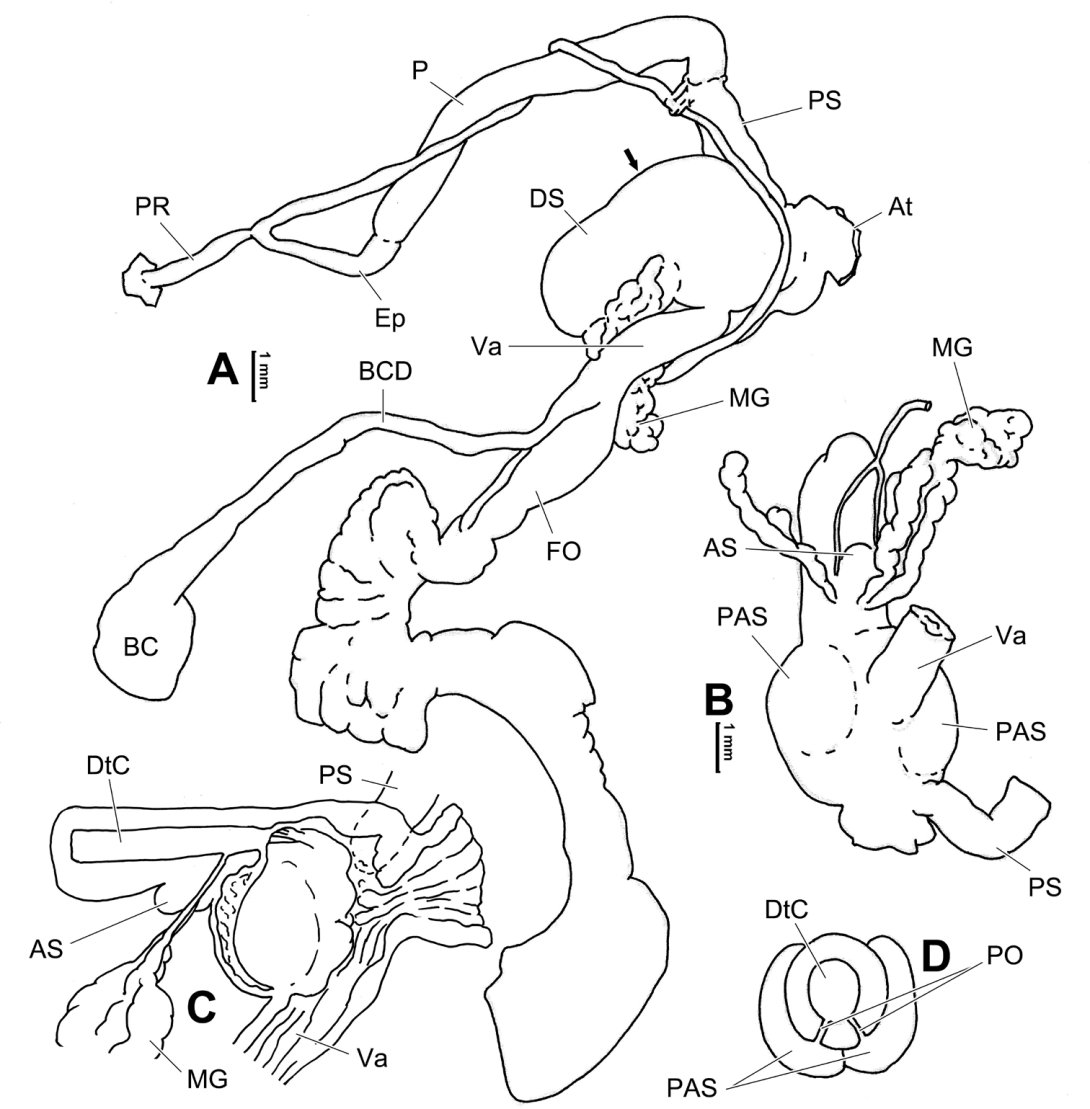
Genital anatomy of *Laeocathaicaqishilii* Wu, sp. nov., HBUMM08298-spec.1, holotype **A** general view **B** ventral view of dart sac apparatus **C** left view of dart sac apparatus **D** cross-section of dart sac at the position arrowed in (**A**). Abbreviations: AS – accessory sac; At – atrium; BC – bursa copulatrix; BCD – bursa copulatrix duct; DS – dart sac; DtC – a chamber containing love dart; Ep – epiphallus; FO – free oviduct; MG – mucous glands; P – penis; PAS – proximal accessory sac; PO – opening of proximal accessory sac leading to dart chamber; PR – penial retractor muscle; PS – penial sheath; Va – vagina; VD – vas deferens.

Figs 2A, B , 4 , 35A, B , 36 , 44C , 48A, B , 49A, B ; Tables 1 , 2 [奇石射带蜗牛]

###### Type material.

***Holotype*** HBUMM08298-spec.1, fma, border of Jiuzhaigou County and Wen County, near point (33.14376°N, 104.246674°E); 2019-IV, coll. Li, Q.-S. ***Paratypes*** HBUMM08298-spec.2–8, 7 fma, 3 fma dissected; same data as holotype. HBUMM06779 and HBUMM06778, 2 fma and 1 fms, 1 fma dissected; Shijiba, Wen County, 1193 m a.s.l., 33.102222°N, 104.335556°E, 2011-VIII-10, coll. Wu, M., Xu, Q. and Budha, P.

###### Measurement of holotype.

Shell height 8.1 mm, maximum diameter 23.7 mm, aperture height 5.6 mm, aperture breadth 10.6 mm, umbilicus diameter 5.6 mm, protoconch whorls 1^1^/_2_, whorls 5^5^/_8_.

###### Diagnosis.

Protoconch with decussate radial and spiral threads. Umbilicus broad and deep, through which protoconch is visible. A bright band present between carina and suture. Beneath carina a dark chestnut band present. Mucous glands two. Penis with two proximal thick internal pilasters fusing into a Y-shaped fork at proximal 1/3. Vagina between atrium and dart sac not elongated. Two proximal accessory sacs on both sides of dart sac, with two pores leading to opening of dart chamber.

###### Description of shell.

Sinistral, depressed, thin but somewhat solid. Shell with 5^1^/_2_–5^7^/_8_ fairly flat whorls. Suture impressed. Protoconch 1^1^/_2_–1^5^/_8_ whorls, with decussate radial and spiral threads, on the first whorl of which may be invisible because of weathering or erosion. Growth lines fine, more or less clear. Aperture oblique, peach-shaped, descending. Peristome expanded and reflexed at lower part. Columella oblique. Umbilicus broad, ~ ^1^/_3_ of maximum diameter. Protoconch visible through umbilicus. Shell apically in chestnut except white carina, after the first three or four whorls a bright band present between carina and adjacent suture. In umbilical view shell distinctly paler in greyish yellow and just beneath carina a chestnut band present.

###### General anatomy.

Eversible head wart present. At mantle edge leaf-shaped appendage absent. On internal body wall of head region between ommatophorous insertions with neither glands nor tiny pits. Body greyish brown, central dorsum with pale longitudinal stripes. Sole dirty white. Jaw arcuate, with 3–5 more or less projecting ribs.

###### Anatomy of genital organs.

Penial sheath short but well developed. Penis of equal thickness, externally simple. Inside penis, two very thick longitudinal pilasters fusing into a Y-shaped fork at proximal 1/3, accompanied with another two thick pilasters, these plasters then change into numerous fine pilasters that distally merge into three short but thick folds near opening of epiphallus. Epiphallic papilla absent. Vas deferens narrow throughout. Vagina between atrium and dart sac not elongated. Accessory sac spherical, solid, inserting into dart sac medially, opening to distal dart chamber. Mucous glands two, each complicatedly branched. Proximal accessory sacs two, symmetrical, dorsally separated and ventrally touching, each with a pore leading to proximal dart chamber. Love dart ~ 6 mm long, apically 2-bladed, medially round-hexagonal. Bursa copulatrix duct equally narrow. Bursa copulatrix pear-shaped.

###### Etymology.

This new species is named after Mr Li, Qi-Shi, who made this work possible with his field work.

###### Ecology.

This species is found under rotten wood.

###### Distribution.

Only known from the type locality.

###### Remarks.

This new species has a unique protoconch on which decussate radial and spiral threads are present compared to the granulation on this part in the other *Laeocathaica* species. The new species is conchologically close to *Laeocathaicaprionotropis*, but the former species is apically more depressed, more broadly umbilicate, has a more expanded peristome, a more elongated aperture due to the more prominent carina, is apically evenly brown, and has a smooth shell surface instead of the finely scaly surface in the latter species. The new species shares the inner structure of the dart sac with *L.prionotropis*. However, they differ in the internal structure of penis: in *L.prionotropis*, partial pilasters merge into a tubercle, which is missing in the new species. They also differ in the number of tubes of the mucous glands.

##### ﻿ Laeocathaica qiminglii

Taxon classificationAnimaliaStylommatophoraCamaenidae

Wu sp. nov.

0F282EF9-FCA0-5274-BFC0-E2E577622069

https://zoobank.org/F7DA5306-7753-4358-BFBC-815547FB064F

Figs 2A, B , 35C, D , 48C, D , 49D , 51 , Tables 1 , 2 , 3 [启明射带蜗牛]

###### Type material.

***Holotype*** HBUMM08422-spec.1, fms, a slope near X496 (32.969442°N, 104.654191°E), Wenxian, Gansu Province; 2019-X-13; coll. Li, Q.-M.; DNA voucher HBUMM08422a. ***Paratypes*** HBUMM08422-spec.2, 1 animal with mature shell but immature genitalia, dissected; same data as holotype. HBUMM08448, 7 fms, east of town of Wenxian, 2020-VIII, coll. Chen, Z.-G.

###### Measurement of holotype.

Shell height 5.2 mm, maximum diameter 13.2 mm, aperture height 2.0 mm, aperture breadth 4.2 mm, umbilicus diameter 5.4 mm, protoconch whorls 1^1^/_2_, whorls 7^5^/_8_.

###### Diagnosis.

Protoconch without granules. Umbilicus extremely broad, ~ ^1^/_2_ of maximum diameter. Shell evenly pale brown with umbilicus side paler.

###### Description of shell.

Sinistral, fairly depressed, solid. Shell with 7^5^/_8_–7^3^/_4_ fairly flat whorls. Suture impressed. Spire depressed-cone-shaped. Protoconch 1^1^/_2_ whorls, smooth on the first whorl where sculpture may be erased by weathering or erosion, followed by sparse radial threads. Protoconch visible through umbilicus. Growth lines unclear. Spiral grooves absent. After ~ 2^1^/_2_ whorls, with regularly spaced thick ribs between which are many fine threads. Aperture oblique, peach-shaped, descending in front. Body whorl sharply carinate above periphery. Peristome seldom expanded and only slightly reflexed at lower part. Columella very oblique. Umbilicus very broad, ~ ^1^/_2_ of maximum diameter. Shell evenly pale brown with umbilicus side paler.

###### General anatomy.

Eversible head wart small but prominent. At mantle edge leaf-shaped appendage absent. On internal body wall of head region between ommatophorous insertions with neither glands nor tiny pits. Body greyish brown, central dorsum with pale longitudinal stripes. Sole dirty white. Jaw arcuate, with five projecting ribs.

###### Anatomy of genital organs.

Penial sheath present. Vagina between atrium and dart sac not elongate. Mucous glands four (observations of the genitalia of this species are only based on the paratype HBUMM08422-spec.2).

###### Etymology.

This new species is named after the collector Mr. Qiming Li.

###### Ecology.

In October, this species was found in the crevices of broken stones on dry slope.

###### Distribution.

Only known from the type locality.

###### Remarks.

This species has the strongest ribs on the shell surface and the broadest umbilicus of all species of *Laeocathaica*.

##### ﻿ Laeocathaica zhengpingliui

Taxon classificationAnimaliaStylommatophoraCamaenidae

Wu sp. nov.

16C587B4-309A-5E09-B489-40415C74B751

https://zoobank.org/3F6DC3CB-8F91-4E64-93F6-96174797F396

**Figure 37.**
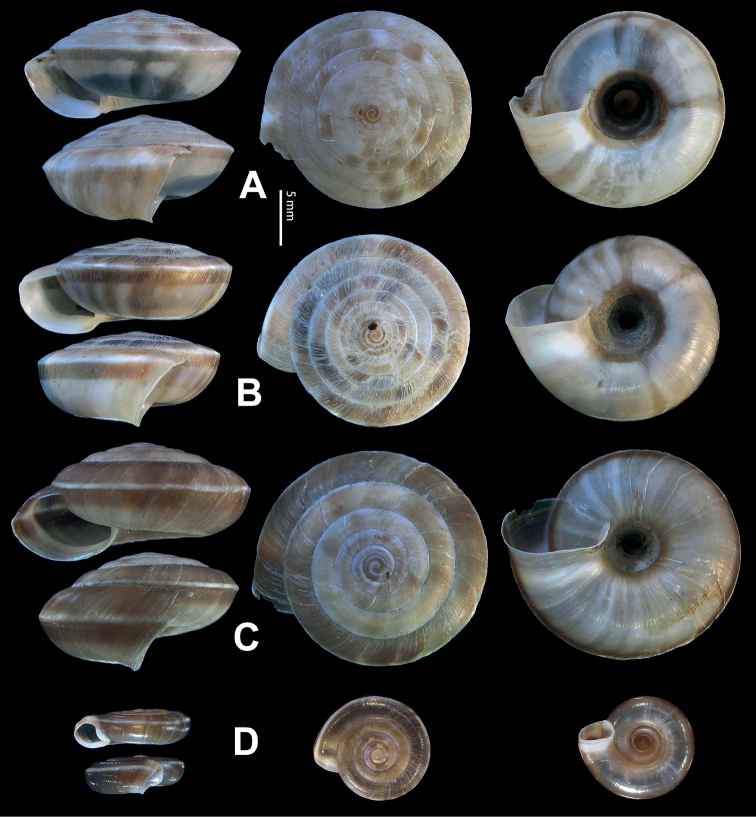
**A***Laeocathaicazhengpingliui* Wu, sp. nov., HBUMM05553-spec.1, holotype **B***L.cheni* Wu, sp. nov., HBUMM05553b-spec.1, holotype **C***L.qingchuanensis* Wu, sp. nov., HBUMM01179a-spec.1, holotype **D***L.nordsiecki* Wu, sp. nov., HBUMM08446-spec.1, holotype.

**Figure 38.**
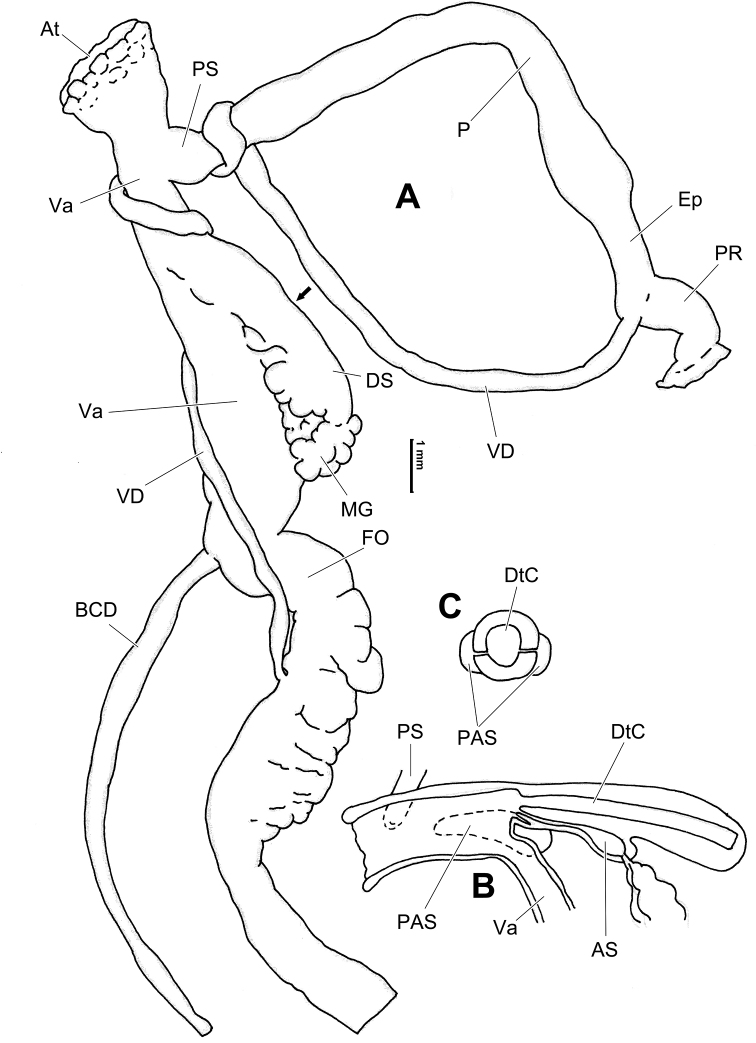
Genital anatomy of *Laeocathaicazhengpingliui* Wu, sp. nov., HBUMM05553-spec.1, holotype **A** general view **B** right view of dart apparatus **C** cross-section of dart sac at the position arrowed in (**A**). Abbreviations: AS – accessory sac; At – atrium; BCD – bursa copulatrix duct; DS – dart sac; DtC – a chamber containing love dart; Ep – epiphallus; FO – free oviduct; MG – mucous glands; P – penis; PAS – proximal accessory sac; PR – penial retractor muscle; PS – penial sheath; Va – vagina; VD – vas deferens.

Figs 2A , 2B , 37A , 38 , 44D , 47K , 47L ; Tables 1 , 2 [正平射带蜗牛]

###### Type material.

***Holotype*** HBUMM05553-spec.1, fma, Hengdan, Wenxian, Gansu Province; north side of Baishuijiang River, along 212 Guodao, near point (32.864025°N, 104.859517°E); hillside, bushes; 2006-IX-29; coll. Wu, M., Liu, J.-M., Zheng, W. and Gao, L.-H.; DNA voucher HBUMM05527. ***Paratypes*** HBUMM05553-spec.2–49, 49 shells including 3 fms (1 broken), 15 juvs and 41 fma, same data as holotype. Ten shells randomly selected from 43 fully mature shells were measured. 2 fma dissected (anatomy no. sp2). HBUMM05565b, 1 fms without protoconch, 9 juvs; Hengdan, Wenxian, Gansu Province; north side of Baishuijiang River, along 212 Guodao, top of hill; 2006-IX-29, Wu, M., Liu, J.-M., Zheng, W. and Gao, L.-H. HBUMM05576c, 1 fma with partially broken shell, dissected; same data as HBUMM05565b.

###### Measurement of holotype.

Shell height 10.0 mm, maximum diameter 19.6 mm, aperture height 5.6 mm, aperture breadth 7.5 mm, umbilicus diameter 7.0 mm, protoconch whorls 1^5^/_8_, whorls 9^1^/_4_.

###### Diagnosis.

Protoconch without granules. Umbilicus more than ^1^/_3_ maximum diameter, through which protoconch is visible. Beneath carina a chestnut band present. Palatal with two blunt teeth. Mucous glands six. Proximal 4/5 of penis with ~ 6 thick internal pilasters. Two adjacent pairs of penial pilasters fusing into two Y-shaped forks at distal 2/3 of penis. Vagina between atrium and dart sac moderately elongated. Proximal accessory sacs two, separate, symmetrical, each with a pore leading to opening of accessory sac.

###### Description of shell.

Sinistral, depressed, thin but somewhat solid. Shell with 8^5^/_8_–9^1^/_4_ fairly flat whorls. Suture impressed. Protoconch 1^1^/_2_–1^3^/_4_ whorls, with very fine axial striae which may be invisible on the first whorl possibly by weathering or erosion. Growth lines thick or rib-like above but fine beneath carina. Above periphery a sharp whitish carina present. Aperture oblique, peach-shaped, slightly descending in front. On ring-like thickening within aperture, a blunt tooth present near columella and another one near carina. Peristome almost not expanded, just minutely reflexed at lower part. Columella oblique. Umbilicus with a tint of pale brown, broadly conical, more than 1/3 of maximum diameter. Protoconch visible through umbilicus. Whorls apically in yellowish white with intermittent brownish patches. In umbilical view shell yellowish white with several brownish patches, and just beneath carina a chestnut band present.

###### General anatomy.

Eversible head wart lowly present. At mantle edge leaf-shaped appendage absent. On internal body wall of head region between ommatophorous insertions with neither glands nor tiny pits. Body greyish brown, central dorsum with pale longitudinal stripes. Sole dirty white. Jaw arcuate, with ~ 6 more or less projecting ribs.

###### Anatomy of genital organs.

Penial sheath very short. Penis distally slightly expanded, externally simple. Penis of proximal 4/5 internally with ~ 6 thick longitudinal pilasters, two adjacent pairs of which fuse into two Y-shaped forks at distal 2/3; pilasters then branching into fine pilasters that are connected to form network. Epiphallic papilla absent. Vas deferens narrow throughout. Vagina between atrium and dart sac somewhat elongated. Accessory sac spherical, empty, inserting into dart sac at middle part, opening near the opening of dart chamber. Mucous glands six, each a single tube or simply branched. Proximal accessory sacs two, separate, symmetrical, each with a pore leading to opening of accessory sac/dart chamber. Love dart ~ 5 mm long, rounded and bladeless throughout. Bursa copulatrix duct equally narrow.

###### Etymology.

This new species is named after Mr. Liu, Zheng-Ping [刘正平], an amateur Chinese conchologist.

###### Ecology.

This species is found on exposed slate rocks of hill side.

###### Distribution.

This species is only known from the type locality.

###### Remarks.

The new species is conchologically close to *Laeocathaicaodophora*; however, its carina is blunter, the aperture has only two weak teeth near the columella instead of two strong apertural teeth in the latter species, and the umbilicus is significantly broader. Regarding genitalia, the new species has two separated proximal accessory sacs of equal size, while in *Laeocathaicaodophora* two proximal accessory sacs are ventrally adjacent and separated only by a very thin membrane, and the much smaller right proximal accessory sac makes *L.odophora* have an asymmetrical dart sac.

##### ﻿ Laeocathaica cheni

Taxon classificationAnimaliaStylommatophoraCamaenidae

Wu sp. nov.

20A61D03-F1AD-5369-92C9-B45322C7D8FE

https://zoobank.org/C1A30E6B-FB66-4BB1-9C24-908CCCCFFA6B

**Figure 39.**
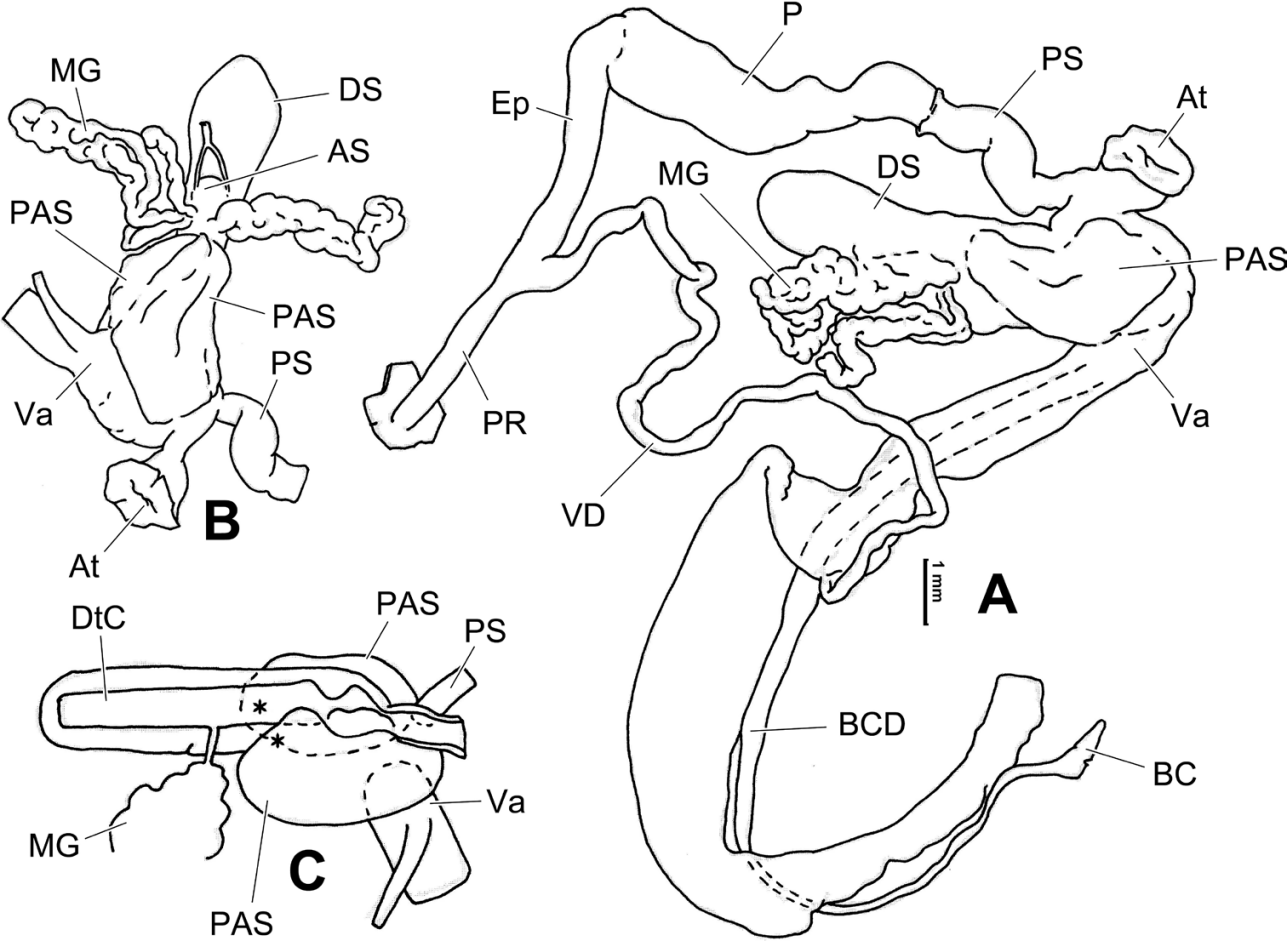
Genital anatomy of *Laeocathaicacheni* Wu, sp. nov., HBUMM05553b-spec.1, holotype **A** general view **B** ventral view of dart apparatus **C** left view of dart sac apparatus. Abbreviations: AS – accessory sac; At – atrium; BC – bursa copulatrix; BCD – bursa copulatrix duct; DS – dart sac; DtC – a chamber containing love dart; Ep – epiphallus; MG – mucous glands; P – penis; PAS – proximal accessory sac; PR – penial retractor muscle; PS – penial sheath; Va – vagina; VD – vas deferens. Asterisk * indicates the opening of proximal accessory sac.

Figs 2A, B , 37B , 39 , 44F–H , 46K, L , 49F , 51 ; Tables 1 , 2 , 3 [陈氏射带蜗牛]

###### Type material.

***Holotype*** HBUMM05553b-spec.1, fma, dissected; Hengdan, Wenxian, Gansu Province; north side of Baishuijiang River, along 212 Guodao, near point (32.864025°N, 104.859517°E); hillside, bushes; 2006-IX-29; coll. Wu, M., Liu, J.-M., Zheng, W. and Gao, L.-H. ***Paratypes*** HBUMM05553b-spec. 2–7, 8 fma, 1 dissected (anatomy no. sp3); same data as holotype. HBUMM05576a- spec. 1–2, 2 fms, South bank of Baishuijiang River, Hengdan, Wenxian, Gansu Province; near point (32.863381°N, 104.854879°E); hilltop, bushes; 2006-IX-29; coll. Wu, M., Liu, J.-M., Zheng, W. and Gao, L.-H. HBUMM08420, 1 fma with fully mature shell but immature genitalia, dissected, a slope near X496 (32.969442°N, 104.654191°E), Wenxian, Gansu Province; 2019-X-13; coll. Li, Q.-M.; DNA voucher HBUMM08420a. HBUMM08428, 3 fma (1 dissected) and 1 juv, same data as HBUMM08420; DNA voucher HBUMM08428a. HBUMM08368, 2 fma and 3 juvs, not dissected; Gansu Province; 2019; coll. Li, Q.-S.; DNA voucher HBUMM08368a.

###### Measurement of holotype.

Shell height 9.0 mm, maximum diameter 19.3 mm, aperture height 5.6 mm, aperture breadth 7.3 mm, umbilicus diameter 7.1 mm, protoconch whorls 1^5^/_8_, whorls 9^1^/_8_.

###### Diagnosis.

Protoconch with elongated granules. Umbilicus suddenly narrowed from penultimate whorl, more than ^1^/_3_ maximum diameter, through which protoconch is visible. Beneath carina a chestnut band present. Mucous glands four. Proximal 1/2 of penis with ~ 5 thin internal pilasters, two of them fusing into a Y-shaped fork at middle part. Distal region of penis with tongue-shaped papillae. Vagina between atrium and dart sac not elongated. Proximal accessory sacs two, symmetrical, each with a pore leading to dart chamber.

###### Description of shell.

Sinistral, depressed, somewhat solid. Shell with 9–9^5^/_8_ fairly flat whorls. Suture impressed. Protoconch 1^1^/_2_–1^5^/_8_ whorls, with fine granules (each ~ 20 µm long) almost invisible because of weathering or erosion. Growth lines thick or rib-like above but fine below carina. Above periphery a whitish carina present. Aperture oblique, peach-shaped, descending. On ring-like thickening within aperture, a very blunt tooth present near columella. Peristome almost not expanded, just minutely reflexed at lower part. Columella oblique. Umbilicus brownish, more than 1/3 of maximum diameter, suddenly narrowed from penultimate whorl. Protoconch visible through umbilicus. Whorls apically yellowish brown with intermittent darker brown patches. In umbilical view shell yellowish white with several brownish patches, and just beneath carina a thick chestnut band present.

###### General anatomy.

Eversible head wart weakly present. At mantle edge leaf-shaped appendage absent. On internal body wall of head region between ommatophorous insertions with neither glands nor tiny pits. Body greyish brown, central dorsum with pale longitudinal stripes. Sole dirty white. Jaw arcuate, with ~ 8 more or less projecting ribs.

###### Anatomy of genital organs.

Penial sheath short but well developed. Penis distally expanded, externally simple. Inside penis, proximal 1/2 with ~ 5 internal pilasters, two of which fuse into one Y-shaped fork at middle of penis; distal 1/2 with tongue-shaped (HBUMM05553b) or diamond-shaped papillae (HBUMM08428) that are erect or inclined towards atrium. Fine pilasters on distal end of penis merging into ~ 6 more or less thick short folds. Epiphallic papilla absent. Vas deferens narrow throughout. Vagina between atrium and dart sac not elongated. Accessory sac tiny, internally solid, inserting into dart sac medially, opening to dart chamber. Mucous glands four, each a single tube or simply branched. Proximal accessory sacs two, symmetrical, dorsally separated and ventrally touching each other, each with a ventral pore leading to proximal dart chamber. Love dart ~ 7 mm long, apically 2-bladed or rhombic (HBUMM08428-spec.1) then rounded. Bursa copulatrix duct equally narrow.

###### Etymology.

This new species is named in honor of Prof Chen, De-Niu, who works on land mollusks in the Institute of Zoology, Chinese Academy of Sciences, Beijing.

###### Ecology.

This species is found on exposed slate rocks of the hill side.

###### Distribution.

Only known from the type locality.

###### Remarks.

The new species is conchologically close to *L.zhengpingliui* Wu, sp. nov., but the new species has a much more depressed spire and relatively much larger aperture. In the genitalia, the middle part of penis, i.e., the distal 1/2 of the penis of the new species is occupied with regular tongue/diamond-shaped papillae (Fig. 44F, H), while in *L.zhengpingliui* Wu, sp. nov., the middle part is clearly short (only ~ 1/6 of the penis length) and does not have such tongue/diamond-shaped papillae (Fig. 44D).

##### ﻿ Laeocathaica qingchuanensis

Taxon classificationAnimaliaStylommatophoraCamaenidae

Wu sp. nov.

054008F0-3B01-5BDF-BF5C-D5CA214D6AF6

https://zoobank.org/771F6786-2918-4B14-8591-1614D3C2699C

**Figure 40.**
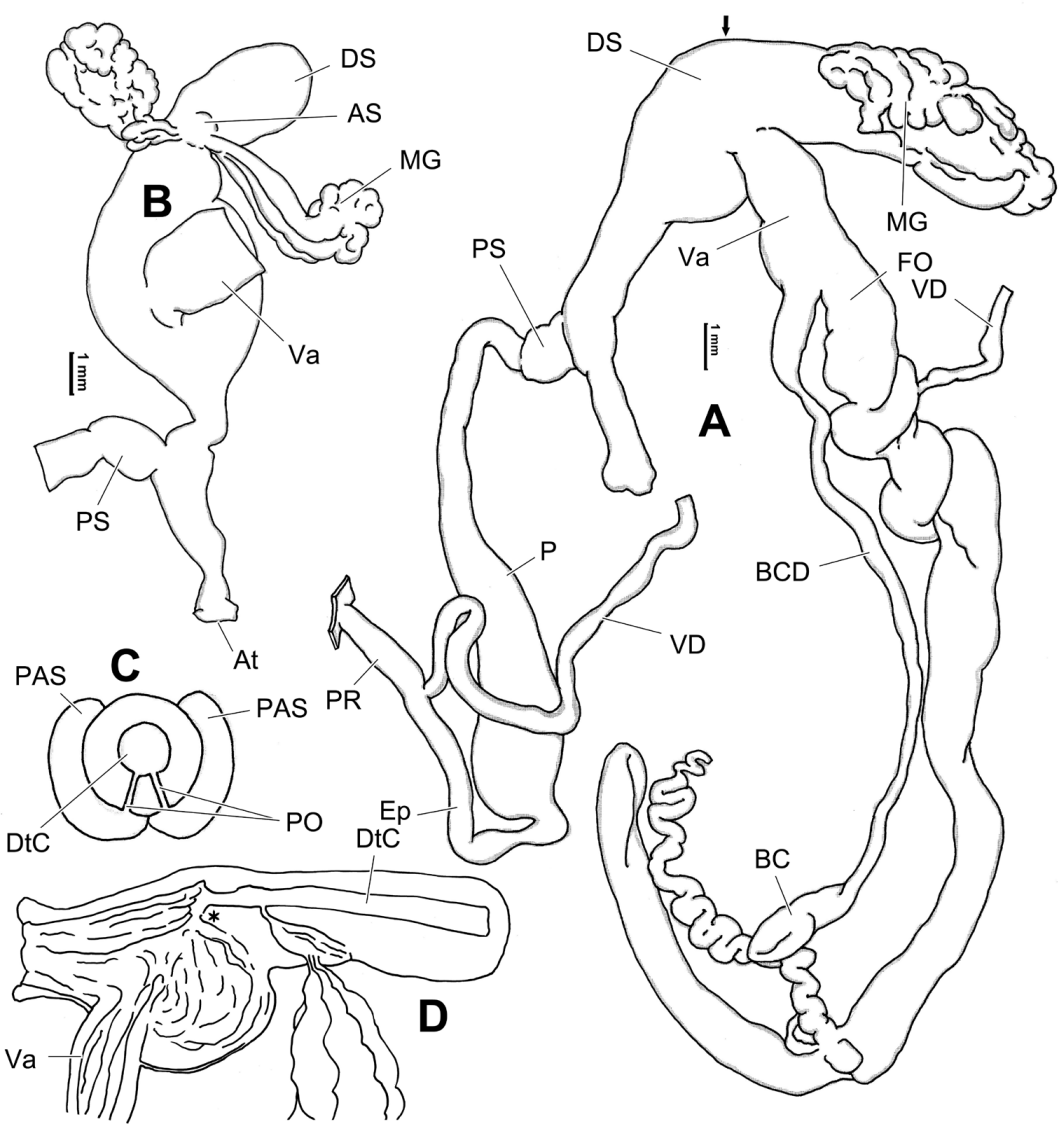
Genital anatomy of *Laeocathaicaqingchuanensis* Wu, sp. nov., HBUMM01179a-spec.1, holotype **A** general view **B** ventral view of dart apparatus **C** cross-section of dart sac at the position arrowed in (**A**) **D** right view of dart sac apparatus. Abbreviations: AS – accessory sac; At – atrium; BC – bursa copulatrix; BCD – bursa copulatrix duct; DS – dart sac; DtC – a chamber containing love dart; Ep – epiphallus; FO – free oviduct; MG – mucous glands; P – penis; PAS – proximal accessory sac; PO – opening of proximal accessory sac leading to dart chamber; PR – penial retractor muscle; PS – penial sheath; Va – vagina; VD – vas deferens. Asterisk * indicates the opening of proximal accessory sac.

Figs 2A, C , 37C , 40 , 44E , 45E, F , 49E , 50B , Tables 1 , 2 [青川射带蜗牛]

###### Type material.

***Holotype*** HBUMM01179a-spec.1, fma, Dagou [大沟] Nature Reserve, Qingchuan County [青川县], Guangyuan, Sichuan Province; limestone and slate, hill foot along stream, from point A (837 m a.s.l., 32.594°N, 105.230638°E) to point B (941 m a.s.l., 32.600888°N, 105.217806°E); 2003-VII-13; coll. Wu, M. ***Paratypes*** HBUMM01179a-spec. 2–12, 6 fma (1 protoconch lost) and 5 juvs, 3 fma dissected (anatomy no. sp5); same data as holotype. HBUMM03001, 2 fma and 8 juvs, not measured and not dissected; same coll. data as holotype. HBUMM08195, 1 dissected, Qingchuan, Guangyuan, Sichuan Province, 490 m (SC-QC-040), 32.525556°N, 105.591472°E; near roots of grasses and on rocks, treeless; 2017-VIII-6, coll. Sheng, X.-F. etc.; DNA voucher HBUMM08195a. HBUMM08196, 6 fma and 3 fms,coll. data as HBUMM08195; DNA voucher HBUMM08196a. HBUMM08200, 12 fma and 1 subadult, not dissected, not measured; Qingchuan, Guangyuan, Sichuan Province, 511 m (SC-GY-043), 32.238889°N, 106.08525°E; on rocks and grass leaves, very thin litter layer; 2017-VIII-6, coll. Sheng, X.-F. etc.

**Table 3.** Voucher information and the GenBank accession numbers of the species in the phylogenetic study. An asterisk (*) indicates obtained from NCBI. The generic assignment for species follows MolluscaBase (2021a–p; 2022a–c).

Taxa	GenBank accession numbers 16S/ITS2	Museum voucher	Voucher information
***Acusta* E. von Martens, 1860**			
*Acustasieboldiana* (L. Pfeiffer, 1850)	ON261686/ON261774	HBUMM04882_2	Fukuoka, Japan; coll. Guo, J.-Y. [郭建英], 2004-X-12
	ON261687/ON261775	HBUMM04882_3	
*Acustarhodostoma* (Möllendorff, 1884)	ON261688/ON261776	HBUMM05089c2	Haikou, Hainan; coll. Wu, M., 2005-VIII-15
	ON261689/ON261777	HBUMM05089c4	
***Aegista* Albers, 1850**			
*Aegistachinensis* (Philippi, 1845)	ON261690/ON261778	HBUMM06480	Zhenjiang, Jiangsu; coll. Wu, M., Xu, Q., 2011-VI-12
***Bradybaena* H. Beck, 1837**			
*Bradybaenabrevispira* (H. Adams, 1870)	ON261691/ON261779	HBUMM05104_2	Zhongxian, Chongqing; coll. Wu, M., 2004-VII-16
	ON261692/ON261780	HBUMM05122_2	Fengjie, Chongqing; coll. Wu, M., 2004-VII-18
	ON261693/ON261781	HBUMM05122_3	
	ON261694/ON261782	HBUMM05112_2	Guangan [广安], Sichuan; coll. Wu, M.
*Bradybaenacontroversamonotaeniata* Pilsbry, 1934	ON261695/ON261783	HBUMM01031_2	Maoxian, Sichuan; coll. Wu, M., 2001-X-11
	ON261696/ON261784	HBUMM01031_3	
*Bradybaenaerispachychila* (Möllendorff, 1899)	ON261697/ON261785	HBUMM05493_3	Wenxian, Gansu; coll. Wu, M., 2006-IX-28
*Bradybaenaqixiaensis* Wu & Asami, 2017	MW810081*/ON261787	HBUMM06900_1/HBUMM06900_2	Qixiashan [栖霞山], Jiangsu; coll. Wu, M.
*Bradybaenasimilaris* (Rang, 1831)	HQ245444*/AY014138*	/	Koehler, unpublished data; Wade et al. 2001
*Bradybaenastrictotaenia* (Möllendorff, 1899)	ON261701/ON261790	HBUMM05406_2	Wenxian, Gansu; coll. Wu, M., 2006-IX-27
	ON261702/ON261791	HBUMM05406_3	
	ON261699/ON261788	HBUMM05446_2	Wenxian, Gansu; coll. Wu, M., 2006-IX-27
	ON261700/ON261789	HBUMM05446_3	
*Bradybaenasueshanensis* Pilsbry, 1934	ON261703/ON261792	HBUMM06603	Jiuzhaigouxian, Sichuan; coll. Wu, M., 2011-VIII-11
*Bradybaenatwenhuaensis* (Ping & Yen, 1932)	ON261704/ON261793	HBUMM08276a1	Changbaishan [长白山], Jilin; coll. Shen, X.-F., 2018-VIII-18
	ON261705/ON261794	HBUMM08276a2	
***Buliminidius* Heude, 1890**			
*Buliminidiusachatininus* (Möllendorff, 1899)	ON261758/ON261848	HBUMM06678	Wenxian, Gansu; coll. Wu, M., 2011-VIII-9
*Buliminidiushirsutus* (Möllendorff, 1899)	ON261759/ON261849	HBUMM06676	Wenxian, Gansu; coll. Wu, M., 2011-VIII-9
***Camaenella* Pilsbry, 1893**			
*Camaenellaplatyodon* (L. Pfeiffer, 1846)	ON261706/ON261795	HBUMM05089a_2	Haikou [海口], Hainan; coll. Wu, M., 2005-VIII-15
	ON261707/ON261796	HBUMM05089a_3	
	ON261708/ON261797	HBUMM05089a_4	
***Cathaica* Möllendorff, 1884**			
*Cathaicaottoi* Pilsbry, 1934	ON261709/ON261798	HBUMM08358a1	Wenchuanxian, Sichuan; coll., Liu, Z.-P., 2019-VII-5
	ON261710/ON261799	HBUMM08358a3	
*Cathaicafasciola* (Draparnaud, 1801)	ON261711/ON261800	HBUMM05168_1	Haidian [海淀], Beijing; coll. Wu, M., 2006-VII
	ON261712/ON261801	HBUMM05368_3	Qingdao [青岛], Shandong; coll. Wu, M., 2006-X-17
*Cathaicagansuica* Möllendorff, 1899	ON261714/ON261803	HBUMM05602cb	Wudu, Gansu; coll. Liu, J.-M. and Zheng, W., 2006-IX-30
	ON261713/ON261802	HBUMM05655	Dangchang, Gansu; coll. Liu, J.-M. and Zheng, W., 2006-X-2
*Cathaicaochthephiloides* Möllendorff, 1899	ON261715/ON261804	HBUMM06560	Jiuzhaigouxian, Sichuan; coll. Wu, M., 2011-VIII-14
*Cathaicapulveratricula* (Martens, 1882)	ON261716/ON261805	HBUMM08208a	Dingxi, Gansu; coll. Shen, X.-F., 2017-VIII-4
***Karaftohelix* Pilsbry, 1927**			
*Karaftohelixmiddendorffi* (Gerstfeldt, 1859)	ON261698/ON261786	HBUMM05933	forest between Vladivostok and Artion cities, Russia; coll. Sayenko, E.M., 2005-VI-26
***Helix* Linnaeus, 1758**			
*Helixpomatia* Linnaeus, 1758	KR705016*/ KR705093*	/	Europe
***Laeocathaica* Möllendorff, 1899**			
*Laeocathaicaamdoana* Möllendorff, 1899	ON261746/ON261835	HBUMM08432a1	See in the text
	ON261741/ON261830	HBUMM08432a2	
*Laeocathaicacarinifera* (H. Adams, 1870)	ON261718/ON261807	HBUMM05103_2	See in the text
	ON261719/ON261808	HBUMM05103_3	
	ON261720/ON261809	HBUMM05131_2	See in the text
	ON261721/ON261810	HBUMM05131_3	
*Laeocathaicacheni* Wu, sp. nov.	ON261727/ON261816	HBUMM08368a	See in the text
	ON261728/ON261817	HBUMM08368a3	
	ON261729/ON261818	HBUMM08368a4	
	ON261751/ON261840	HBUMM08428a2	See in the text
	ON261737/ON261826	HBUMM08428a4	
*Laeocathaicadistinguenda* Möllendorff, 1899	ON261717/ON261806	HBUMM05407_3	See in the text
*Laeocathaicafilippina* (Heude, 1882)	ON261730/ON261819	HBUMM01256b_2	See in the text
	ON261731/ON261820	HBUMM01256b_3	
	ON261732/ON261821	HBUMM05097_2	See in the text
*Laeocathaicaodophora* Möllendorff, 1899	ON261736/ON261825	HBUMM08430a1	See in the text
*Laeocathaicaphaeomphala* Möllendorff, 1899	ON261742/ON261831	HBUMM08424a2	See in the text
*Laeocathaicapolytyla* Möllendorff, 1899	ON261722/ON261811	HBUMM05411_4	See in the text
*Laeocathaicapotanini* Möllendorff, 1899	ON261723/ON261812	HBUMM05409_2	See in the text
	ON261724/ON261813	HBUMM05409_3	
	ON261725/ON261814	HBUMM05409_4	
*Laeocathaicaprionotropis* Möllendorff, 1899	ON261726/ON261815	HBUMM05440_3	See in the text
	ON261744/ON261833	HBUMM08421a1	See in the text
	ON261749/ON261838	HBUMM08421a3	
	ON261748/ON261837	HBUMM08421a4	
	ON261739/ON261828	HBUMM08423a1	See in the text
	ON261747/ON261836	HBUMM08423a2	
*Laeocathaicaqiminglii* Wu, sp. nov.	ON261740/ON261829	HBUMM08422a1	See in the text
*Laeocathaicastenochone* Möllendorff, 1899	ON261743/ON261832	HBUMM08431a1	See in the text
	ON261738/ON261827	HBUMM08431a2	
	ON261750/ON261839	HBUMM08431a3	
*Laeocathaicatropidorhaphe* Möllendorff, 1899	ON261733/ON261822	HBUMM05688	See in the text
	ON261735/ON261824	HBUMM05617	See in the text
	ON261734/ON261823	HBUMM05716	See in the text
	ON261745/ON261834	HBUMM08425a1	See in the text
***Metodontia* Möllendorff, 1886**			
*Metodontiahouaiensis* (Crosse, 1882)	ON261752/ON261841	HBUMM05164_2	Quyang [曲阳], Hebei; coll. Wu, M., Wu, Q., 2006-V-4
	ON261753/ON261842	HBUMM05164_3	
	ON261754/ON261843	HBUMM05164_4	
***Nesiohelix* Kuroda & Emura, 1943**			
*Nesiohelixmoreletiana* (Heude, 1882)	MW810080*/ON261844	HBUMM06796	Hangzhou [杭州], Zhejiang; coll. Wu, M., Xu, Q., 2012-V-21
***Plectotropis* E. von Martens, 1860**			
*Plectotropissterilis* (Heude, 1890)	ON261755/ON261845	HBUMM04909_2	Badong, Hubei; coll. Wu, M., Wu, Q., Qi, G., 2004-VII-31
	ON261756/ON261846	HBUMM04909_3	
***Pseudiberus* Ancey, 1887**			
*Pseudiberusstrophostoma* (Möllendorff, 1899)	ON261757/ON261847	HBUMM06586b	Jiuzhaigouxian, Sichuan; coll. Wu, M., 2011-VIII-14
***Pseudobuliminus* Gredler, 1886**			
*Pseudobuliminuspiligerus* (Möllendorff, 1899)	ON261760/ON261850	HBUMM05412_2	Wenxian, Gansu; coll. Wu, M., 2006-IX-27
*Pseudobuliminussubcylindricus* (Möllendorff, 1899)	ON261761/ON261851	HBUMM06720	Jiuzhaigouxian, Sichuan; coll. Wu, M., 2011-VIII-12
***Satsuma* A. Adams, 1868**			
*Satsumaguandi* Zhang, Zhu & Lyu, 2020	MW804648*/ON261852	HBUMM08239a_1	Shaoguan [韶关], Guangdong; coll. Yu, Di [余迪]
	MW804647*/ON261853	HBUMM08239a_2	
***Stilpnodiscus* Möllendorff, 1899**			
*Stilpnodiscusentochilus* Möllendorff, 1899	ON261762/ON261854	HBUMM05451	Wenxian, Gansu; coll. Wu, M., 2006-IX-27
*Stilpnodiscusmoellendorffi* Wu, 2001	ON261763/ON261855	HBUMM05439_2	Wenxian, Gansu; coll. Wu, M., 2006-IX-27
*Stilpnodiscus* sp.	ON261764/ON261856	HBUMM05491c_2	Wenxian, Gansu; coll. Wu, M., 2006-IX-28
*Stilpnodiscusvernicinus* Möllendorff, 1899	ON261765/ON261857	HBUMM05528	Wenxian, Gansu; coll. Wu, M., 2006-IX-29
***Traumatophora* Ancey, 1887**			
*Traumatophoratriscalpta* (E. von Martens, 1875)	ON261766/ON261858	HBUMM08302a	Wuhan, Hubei; coll. Chen, Zhe-Yu [陈哲宇], 2019-III-20
***Trichobradybaena* Wu & Guo, 2003**			
*Trichobradybaenasubmissa* (Deshayes, 1874)	ON261767/ON261859	HBUMM01221_3	Meitan, Guizhou; coll. Wu, M., 2003-VIII-2
	ON261768/ON261860	HBUMM04904_2	Badong, Hubei; coll. Wu, M., 2004-VIII
	ON261769/ON261861	HBUMM04904_3	
	ON261770/ON261862	HBUMM05095_2	Zigui, Hubei; coll. Wu, M., 2004-VIII-2
	ON261771/ON261863	HBUMM05095_3	
	ON261772/ON261864	HBUMM05123_2	Fengjie, Chongqing; coll. Wu, M., 2004-VII-18
	ON261773/ON261865	HBUMM05132_2	Wuxi, Chongqing; coll. Wu, M., 2004-VII-22

###### Measurement of holotype.

Shell height 10.8 mm, maximum diameter 21.7 mm, aperture height 6.1 mm, aperture breadth 9.2 mm, umbilicus diameter 5.1 mm, protoconch whorls 1^1^/_2_, whorls 6^1^/_4_.

**Figure 41.**
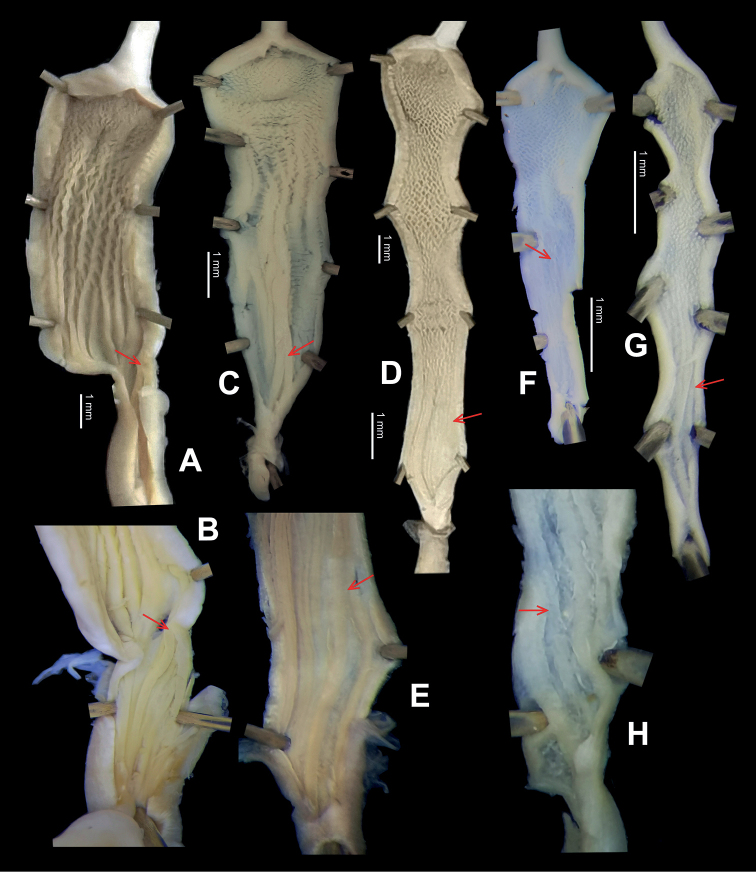
Internal view of penis **A, B***Laeocathaicaamdoana* Möllendorff, 1899, HBUMM05640-spec.1 **A** general view **B** proximal penis **C***L.filippina* (Heude, 1882), HBUMM04166-spec.1, general view **D, E***L.distinguenda* Möllendorff, 1899, HBUMM05436-spec.1 **D** general view **E** proximal penis **F***L.carinalis* Chen & Zhang, 2004, HBUMM08300-spec.1, general view **G, H***L.dityla* Möllendorff, 1899, HBUMM00698-spec.1 **G** general view **H** proximal penis. Arrows indicate the position where two penial internal pilasters fuse into one distal pilaster.

###### Diagnosis.

Protoconch with fine granules. Umbilicus moderately broad, through which protoconch is visible. Bluntly carinate slightly above periphery. Beneath carina a brown band present. Mucous glands 6–8. Approximately 1/2 of penis with three proximal thick internal pilasters, Y-shaped fork formed by adjacent pilasters absent. Vagina between atrium and dart sac moderately elongated. Proximal accessory sacs two, dorsally separated and ventrally touching, each with a pore leading to dart sac chamber near entrance of dart chamber.

**Figure 42.**
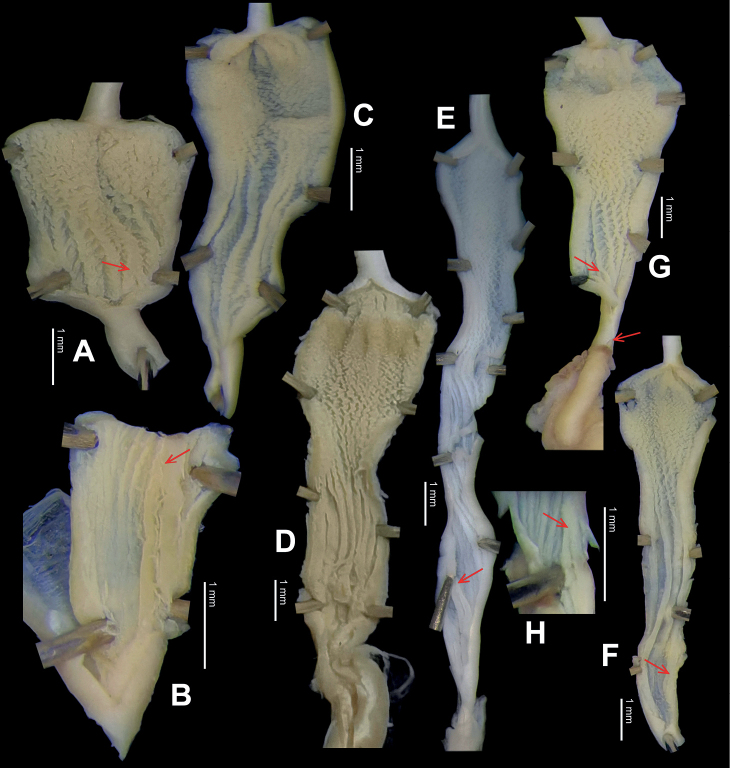
Internal view of penis **A, B***Laeocathaicadolani* (Pilsbry, 1934), HBUMM00069-spec.1 **A** general view **B** proximal penis **C***L.christinae* (H. Adams, 1870), HBUMM01251a-spec.1, general view **D***L.phaeomphala* Möllendorff, 1899, HBUMM05433-spec.1 **E***L.odophora* Möllendorff, 1899, HBUMM08430-spec.1 **F***L.pewzowi* Möllendorff, 1899, ZIN RAS No. 4, “*Aegistapewzowi* Schalf.” **G, H***L.parapolytyla* Wu, sp. nov., HBUMM06640-spec.1, holotype **G** general view **H** proximal part. Arrows indicate the position where two penial internal pilasters fuse into one distal pilaster.

###### Description of shell.

Sinistral, depressed, thin but somewhat solid. Shell with 6^1^/_8_–7 slightly convex whorls. Suture impressed. Protoconch 1^1^/_2_–1^5^/_8_ whorls, fine granules (each ~ 30 – ~ 50 µm long) distinctly present. Growth lines fine and unclear. Aperture oblique, peach-shaped, descending. Within aperture with a white thickening. Peristome expanded and reflexed at lower part. Columella oblique. Umbilicus moderately broad, ~ ^1^/_4_ of maximum diameter. Protoconch visible through umbilicus. Shell apically in pale brown with some intermittent darker patches. Carina slightly above periphery, blunt, white; beneath which a brown band present. Whorls apically in yellowish white with intermittent brownish patches. In umbilical view shell yellowish white with several white radial striations.

**Figure 43.**
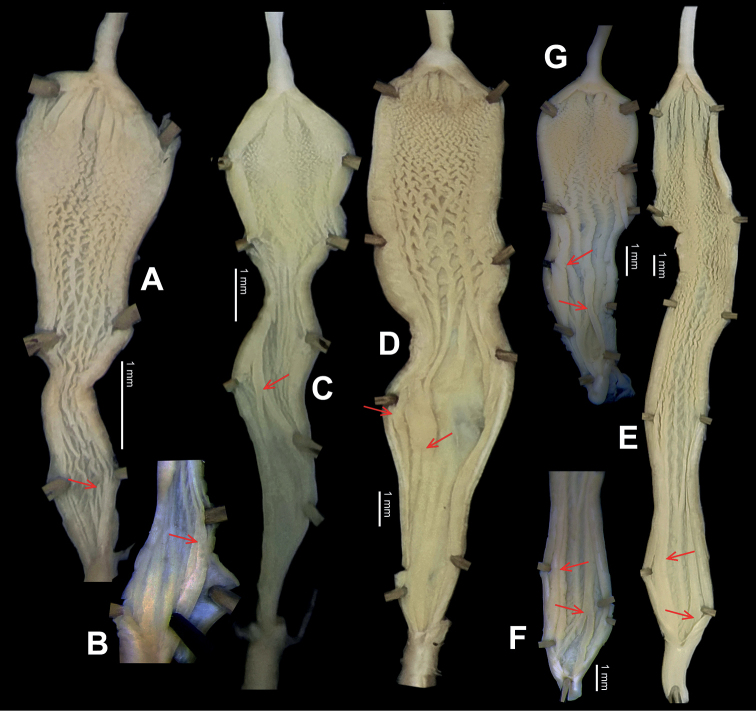
Internal view of penis **A, B***Laeocathaicapolytyla* Möllendorff, 1899, HBUMM05437-spec.1 **A** general view **B** proximal penis **C***L.potanini* Möllendorff, 1899, HBUMM00633-spec.1, general view **D***L.prionotropis* Möllendorff, 1899, HBUMM05549-spec.1, general view **E, F***L.stenochone* Möllendorff, 1899, HBUMM05495-spec.1 **E** general view **F** proximal penis **G***L.carinifera* (H. Adams, 1870), HBUMM08443-spec.2, general view. Arrows indicate the position where two penial internal pilasters fuse into one distal pilaster.

###### General anatomy.

Eversible head wart lowly present. At mantle edge leaf-shaped appendage absent. On internal body wall of head region between ommatophorous insertions with neither glands nor tiny pits. Body greyish brown, central dorsum with pale longitudinal stripes. Sole dirty white. Jaw arcuate, with 4–6 projecting ribs.

###### Anatomy of genital organs.

Penial sheath short. Penis distally swollen, externally simple. Proximal 1/2 penis with four thick internal pilasters which do not fusing, pilasters then branching into numerous fine pilasters that merge into ~ 6 short but thick (thickest in the genus) folds near opening of epiphallus. Vas deferens narrow throughout. Vagina between atrium and dart sac moderately elongated. Accessory sac spherical, internally with high pilasters and fairly solid inside, inserting into dart sac medially, opening to distal dart chamber. Mucous glands 6–8, each single tube or simply branched. PAS two, dorsally separated and ventrally touching, each with a pore leading to dart chamber near dart chamber opening. Proximal bursa copulatrix duct slightly expanded. Bursa copulatrix elongate ovate.

###### Etymology.

This new species is named after name of the type locality Qingchuan, Sichuan Province.

###### Ecology.

This species is found on slate rocks covered with mosses in a humid mountainous environment.

###### Distribution.

Only known from the type locality.

###### Remarks.

**Figure 44.**
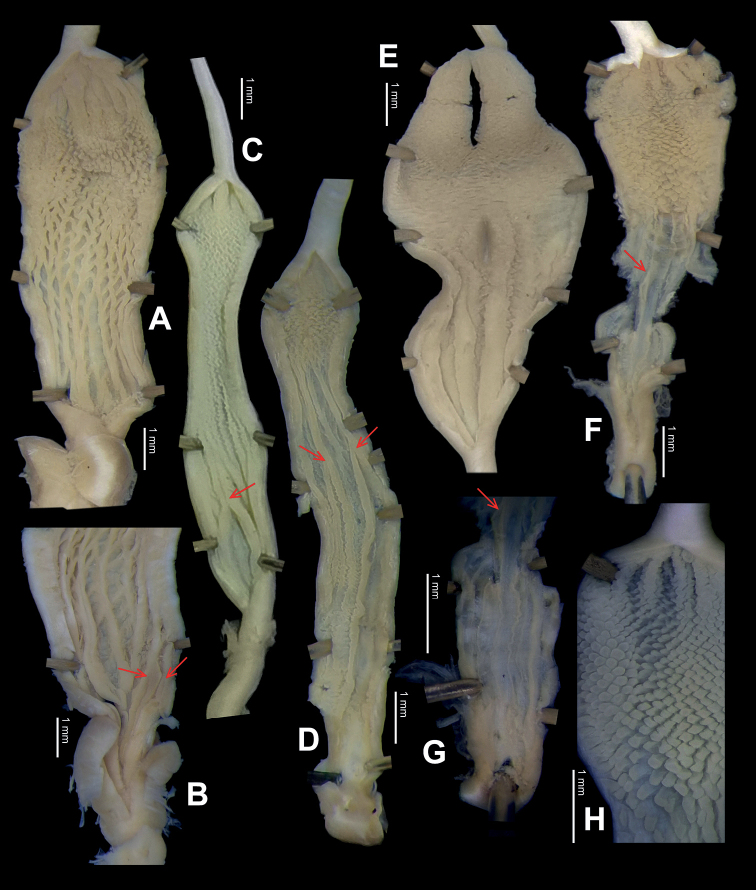
Internal view of penis **A, B***Laeocathaicatropidorhaphe* Möllendorff, 1899 **A** general view, HBUMM05619-spec.1 **B** proximal penis, HBUMM05664-spec.1 **C***L.qishilii* Wu, sp. nov., general view, HBUMM08298-spec.1, holotype **D***L.zhengpingliui* Wu, sp. nov., general view, HBUMM05553-spec.1, holotype **E***L.qingchuanensis* Wu, sp. nov., general view, HBUMM01179-spec.1, holotype **F–H***L.cheni* Wu, sp. nov. **F, G** HBUMM05553b-spec.1, holotype **F** general view **G** proximal penis **H** HBUMM08428-spec.1, paratype, partial internal view of distal penis. Arrows indicate the position where two penial internal pilasters fuse into one distal pilaster.

**Figure 45.**
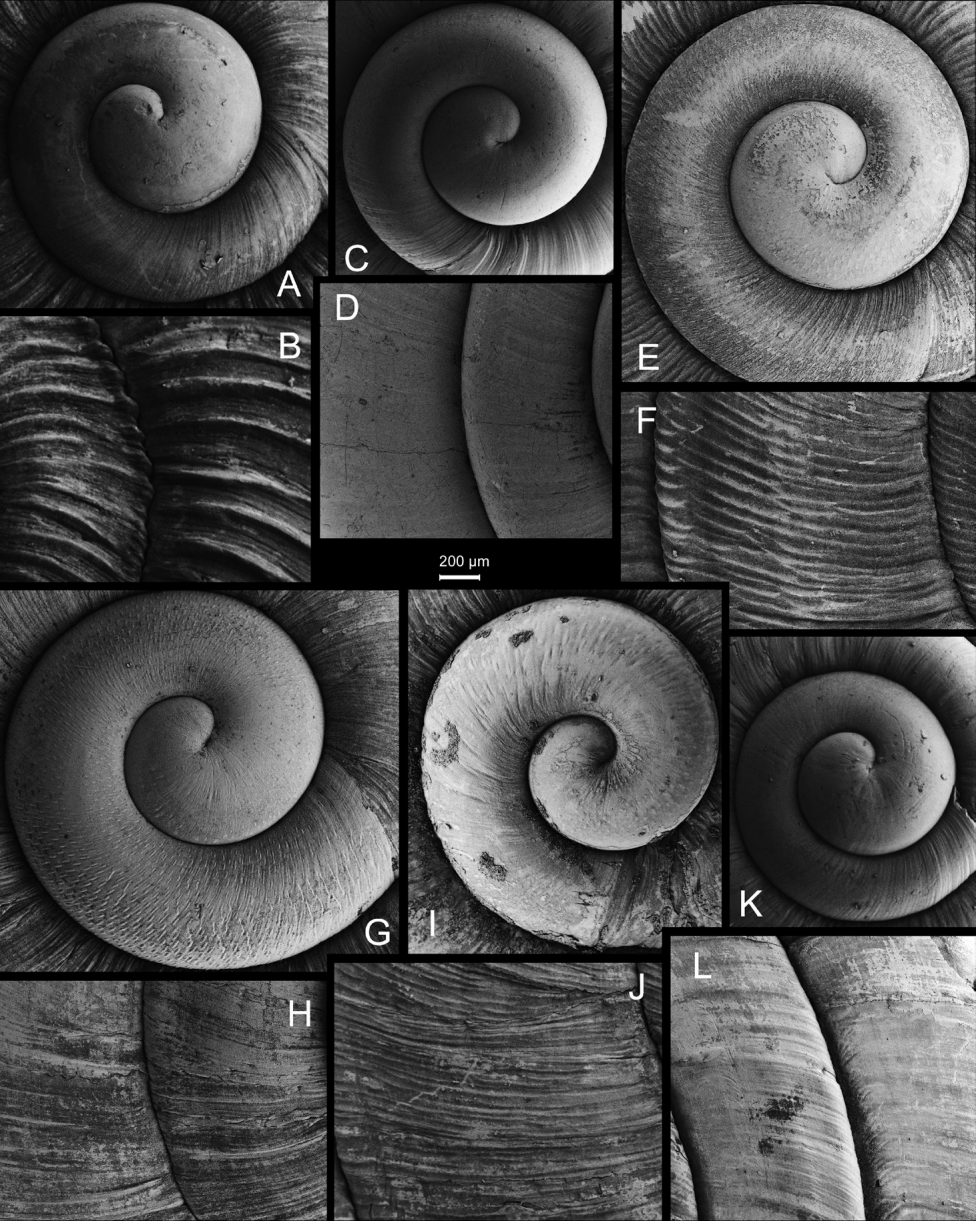
SEM micrographs **A, B***Laeocathaicapotanini* Möllendorff, 1899, HBUMM00633 **A** protoconch **B** teleoconch **C, D***L.dityla* Möllendorff, 1899, HBUMM00698 **C** protoconch **D** teleoconch **E, F***L.qingchuanensis* Wu, sp. nov., HBUMM01179a-spec.6, paratype **E** protoconch **F** teleoconch **G, H***L.christinae* (H. Adams, 1870), HBUMM01251a **G** protoconch **H** teleoconch **I, J***L.carinifera* (H. Adams, 1870), HBUMM04162 **I** protoconch **J** teleoconch **K, L***L.polytyla* Möllendorff, 1899, HBUMM05437 **K** protoconch **L** teleoconch.

*Laeocathaicaqingchuanensis* Wu, sp. nov. is close to *L.stenochone* and *L.carinifera* in general shell shape, aperture shape and coloration, but the new species has a relatively higher shell (Fig. 50B) and the slimmest protoconch granules (Fig. 45E). The new species differs from *Laeocathaicastenochone* and *L.carinifera* in that it has both a long vaginal section above the dart sac and the inner structures of penis, where proximal parallel penial pilasters do not form the Y-shaped fork (Fig. 44E). In addition, compared to *Laeocathaicacarinifera* (Fig. 10), the new species has a very short penial sheath and a pair of symmetrical proximal accessory sac (Fig. 40). Among the aforementioned three species, *Laeocathaicastenochone* has a relatively longer penis.

##### ﻿ Laeocathaica nordsiecki

Taxon classificationAnimaliaStylommatophoraCamaenidae

Wu sp. nov.

4436028B-1012-56E5-AD42-AC719F02AC3C

https://zoobank.org/BBBD09C3-661E-4E70-AA52-59A2B8E818A3

Figs 2A, B , 37D , 47E, F , 49C ; Table 2 [诺氏射带蜗牛]

###### Type material.

***Holotype*** HBUMM08446-spec.1, fully mature shell with immature genital system, Guoyuanxiang [郭元乡], Jiuzhaigouxian, Sichuan Province; near point (33.125506°N, 104.329876°E); 2021-IX-24, coll. Chen, Z.-G. ***Paratypes*** HBUMM08446-spec.2–3, 2 fms; HBUMM08446-spec.4–5, 2 subadults; same data of holotype. HBUMM08447-spec.1–2, 2 fms and 3 living juvs (in rearing), Qinglongcun [青龙村], Guoyuanxiang, Jiuzhaigouxian, Sichuan Province; (33.085615°N, 104.348250°E); 2020-VII, coll. Chen, Z.-G.

###### Measurement of holotype.

Shell height 3.7 mm, maximum diameter 10.8 mm, aperture height 3.0 mm, aperture breadth 3.3 mm, umbilicus diameter 5.0 mm, protoconch whorls 1^1^/_2_, whorls 5^1^/_8_.

###### Diagnosis.

Protoconch with dense fine granules. Umbilicus very broad, ~ ^1^/_2_ of maximum diameter. Body whorl shouldered above periphery. On shoulder with a white band. Peristome continuous. Shell strongly glossy as in *Stilpnodiscus*, almost transparent.

**Figure 46.**
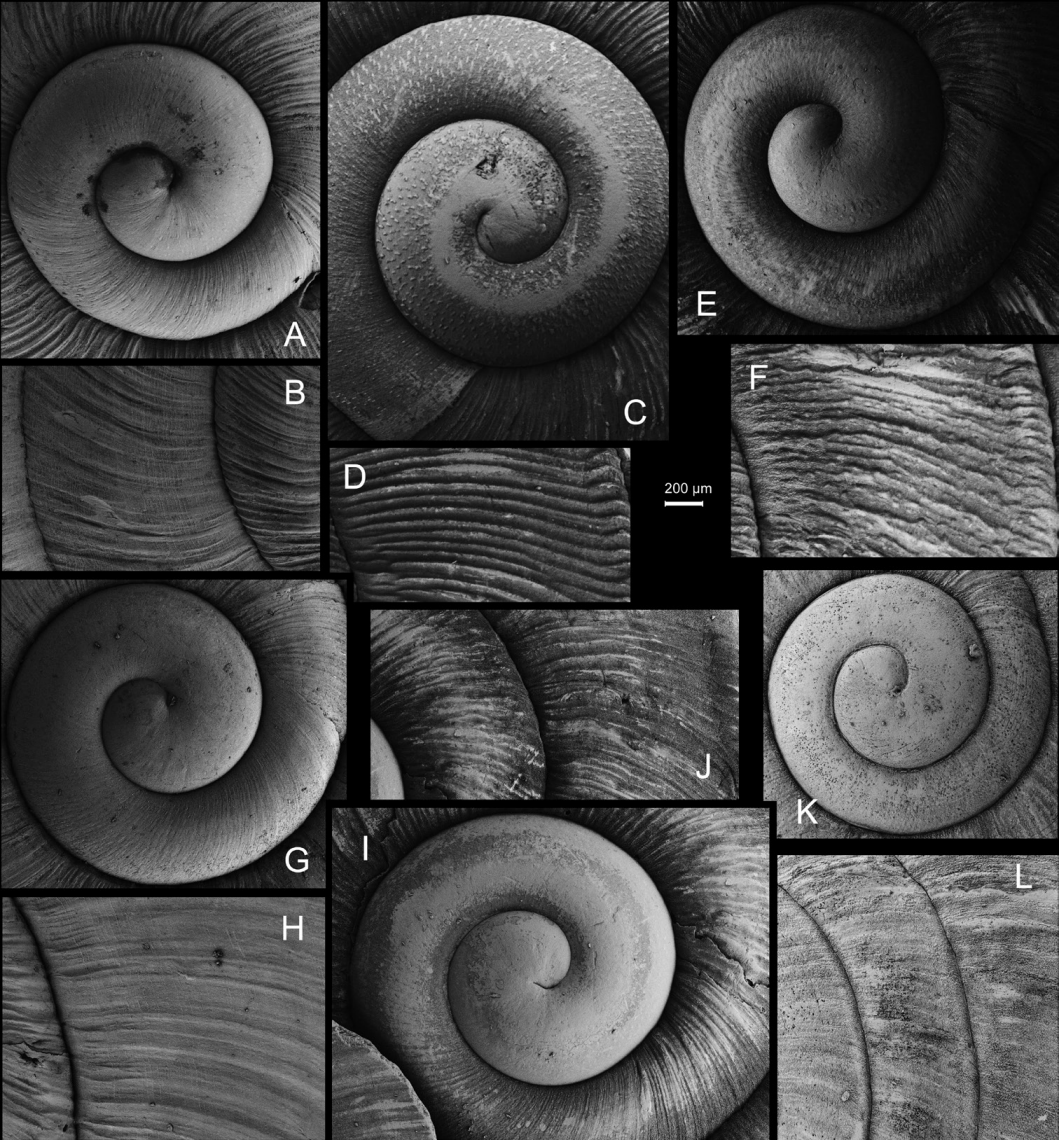
SEM micrographs **A, B***Laeocathaicadistinguenda* Möllendorff, 1899, HBUMM05479 **A** protoconch **B** teleoconch **C, D***L.stenochone* Möllendorff, 1899, HBUMM05495 **C** protoconch **D** teleoconch **E, F***L.prionotropis* Möllendorff, 1899, HBUMM05549 **E** protoconch **F** teleoconch **G, H***L.amdoana* Möllendorff, 1899, HBUMM05640 **G** protoconch **H** teleoconch **I, J***L.tropidorhaphe* Möllendorff, 1899, HBUMM05664 **I** protoconch **J** teleoconch **K, L***L.cheni* Wu, sp. nov., HBUMM08368, paratype, juvenile **K** protoconch **L** teleoconch.

###### Description of shell.

Sinistral, depressed, thin but somewhat solid. Shell with 5–5^1^/_8_ convex whorls. Suture impressed. Protoconch 1^1^/_2_–1^5^/_8_ whorls, densely with tiny granules (each ~ 10 µm long) which are obscured by erosion or weathering. Growth lines indistinct. Shell in pale brown, strongly glossy, almost transparent. Body whorl shouldered above periphery, with a white band on shoulder. Aperture oblique, round, abruptly descending in front. A white thickening within aperture and thickened callus forming a continuous peristome. Peristome expanded and slightly reflexed at lower part. Columella oblique. Umbilicus very broad, ~ ^1^/_2_ of maximum diameter. Protoconch visible through umbilicus.

**Figure 47.**
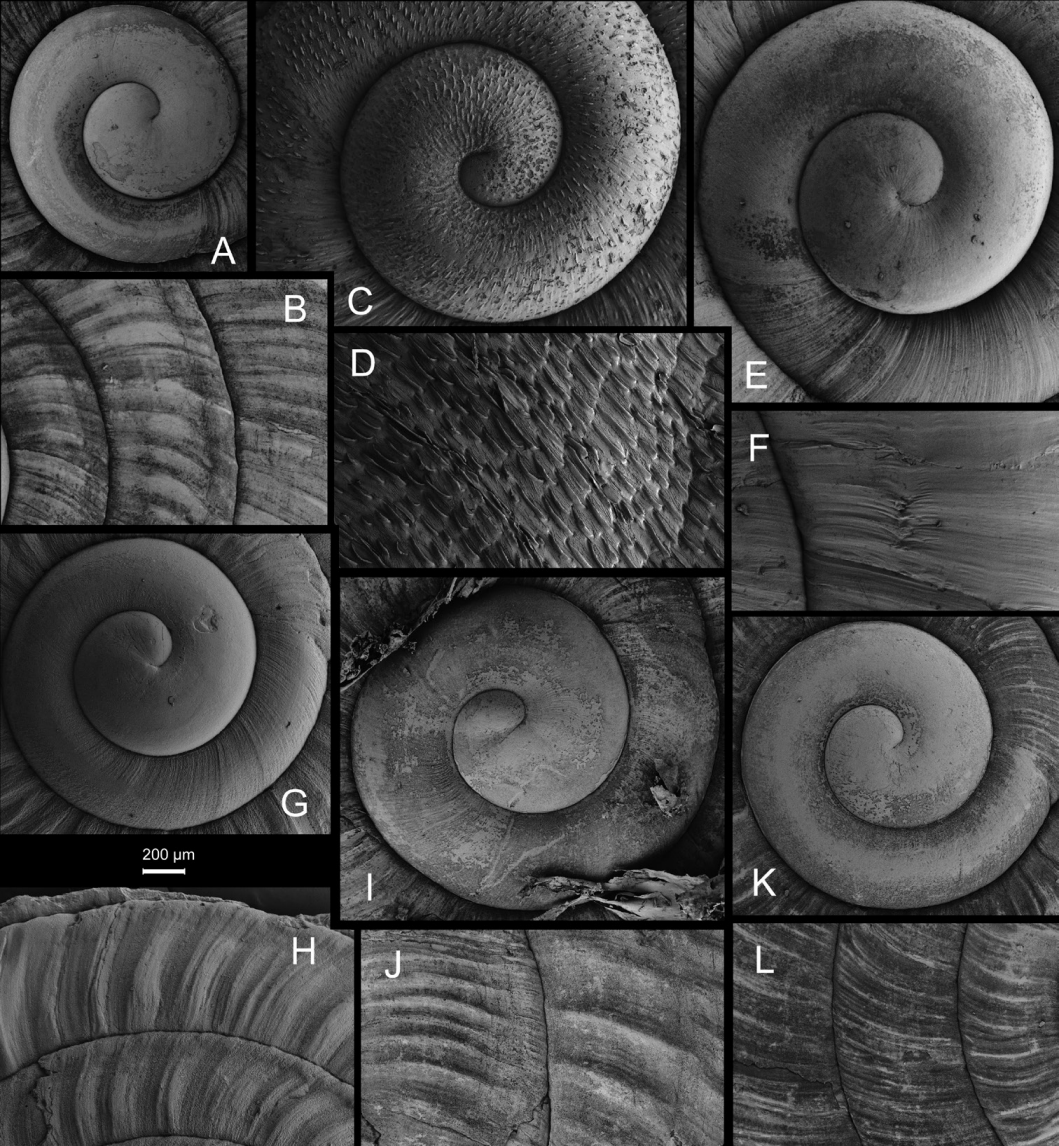
SEM micrographs **A, B***Laeocathaicaodophora* Möllendorff, 1899, HBUMM08430 **A** protoconch **B** teleoconch **C, D***L.dolani* (Pilsbry, 1934), HBUMM08439 **C** protoconch **D** teleoconch **E, F***L.nordsiecki* Wu, sp. nov., HBUMM08446, paratype, subadult **E** protoconch **F** teleoconch **G, H***L.pewzowi* Möllendorff, 1899, HBUMM08452 **G** protoconch **H** teleoconch **I, J***L.carinalis* Chen & Zhang, 2004, HBUMM08453 **I** protoconch **J** teleoconch **K, L***L.zhengpingliui* Wu, sp. nov., HBUMM05553-spec.2, paratype, juvenile **K** protoconch **L** teleoconch.

###### Etymology.

This cute new species is named in memory of Hartmut Nordsieck, a German malacologist who showed strong interest in the snails of the South Gansu Plateau and was a good friend of the first author.

**Figure 48.**
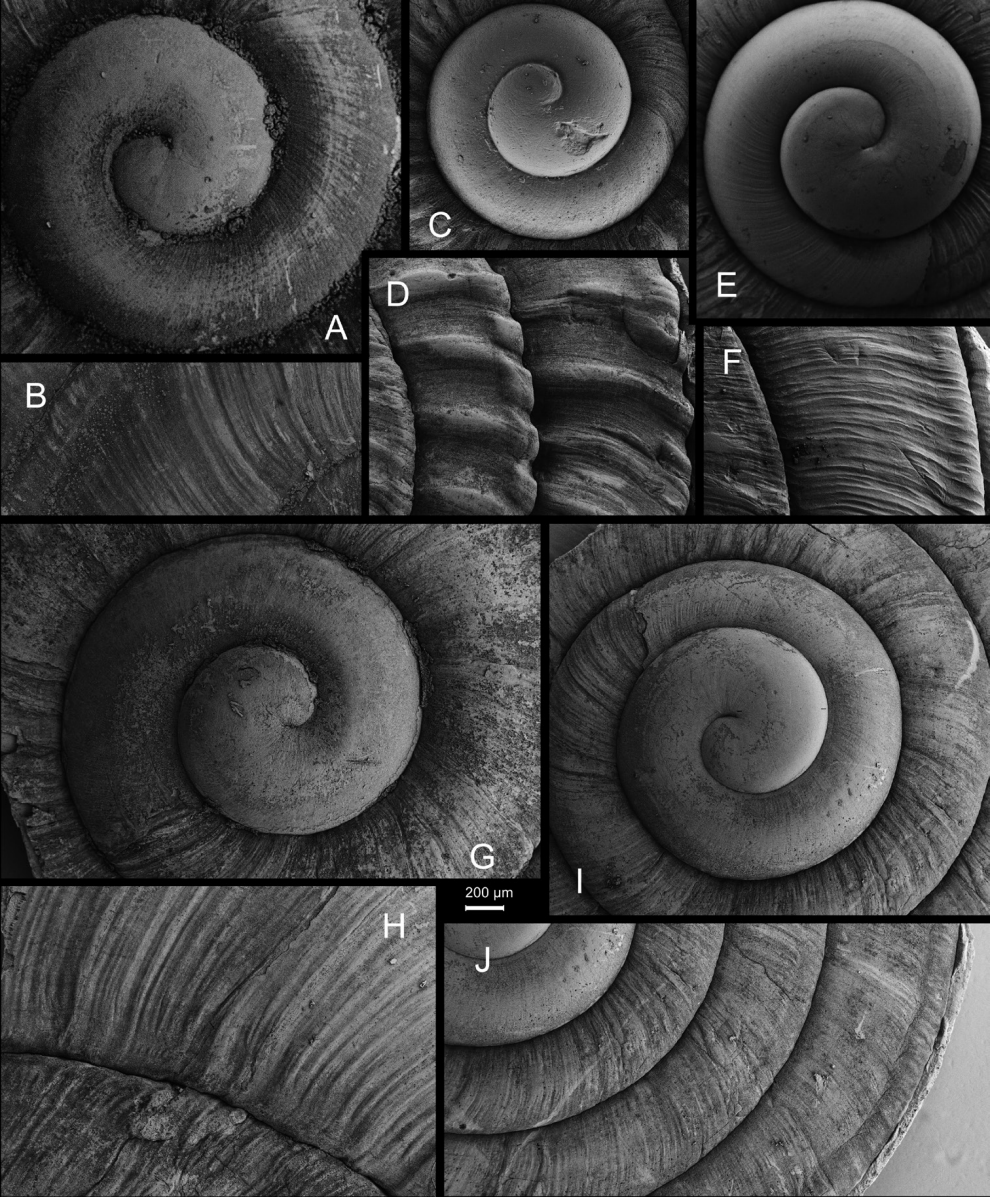
SEM micrographs **A, B***Laeocathaicaqishilii* Wu, sp. nov., HBUMM08298, paratype **A** protoconch **B** teleoconch **C, D***L.qiminglii* Wu, sp. nov., HBUMM08448, paratype **C** protoconch **D** teleoconch **E, F***L.phaeomphala* Möllendorff, 1899, CZG202008-w3, subadult **E** protoconch **F** teleoconch **G, H***L.filippina* (Heude, 1882), HBUMM04166 **G** protoconch **H** teleoconch **I, J***L.parapolytyla* Wu, sp. nov., HBUMM06640-spec.9, paratype **I** protoconch **J** teleoconch.

###### Ecology.

This species was found on bare earth with a few broken rocks.

**Figure 49.**
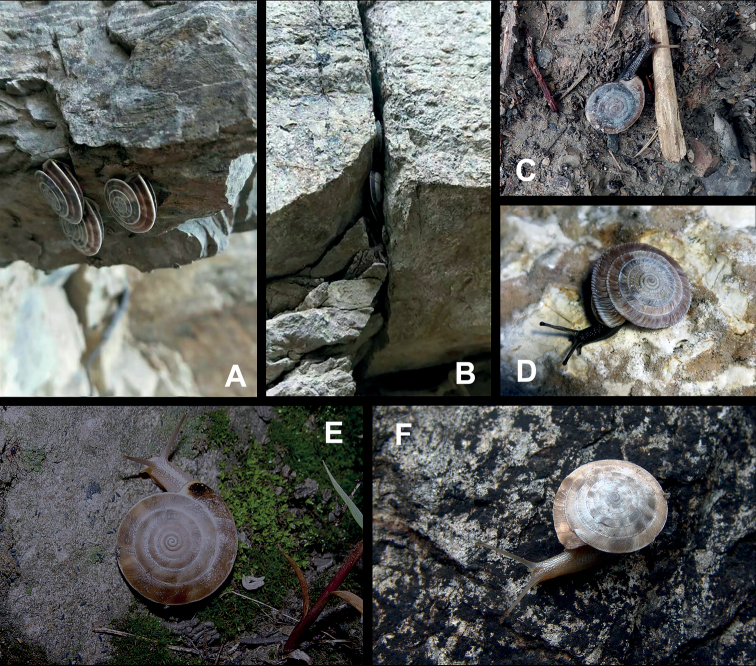
Habitats (excluding **D**) **A, B***Laeocathaicaqishilii* Wu, sp. nov., border of Jiuzhaigou County and Wen County **C***L.nordsiecki* Wu, sp. nov., Guoyuanxiang, Jiuzhaigou **D***L.qiminglii* Wu, sp. nov., an indoor photograph **E***L.qingchuanensis* Wu, sp. nov., Dagou Nature Reserve, Qingchuan County **F***L.cheni* Wu, sp. nov., Hengdan, Wenxian.

###### Distribution.

Only known from two localities where the types were found.

**Figure 50.**
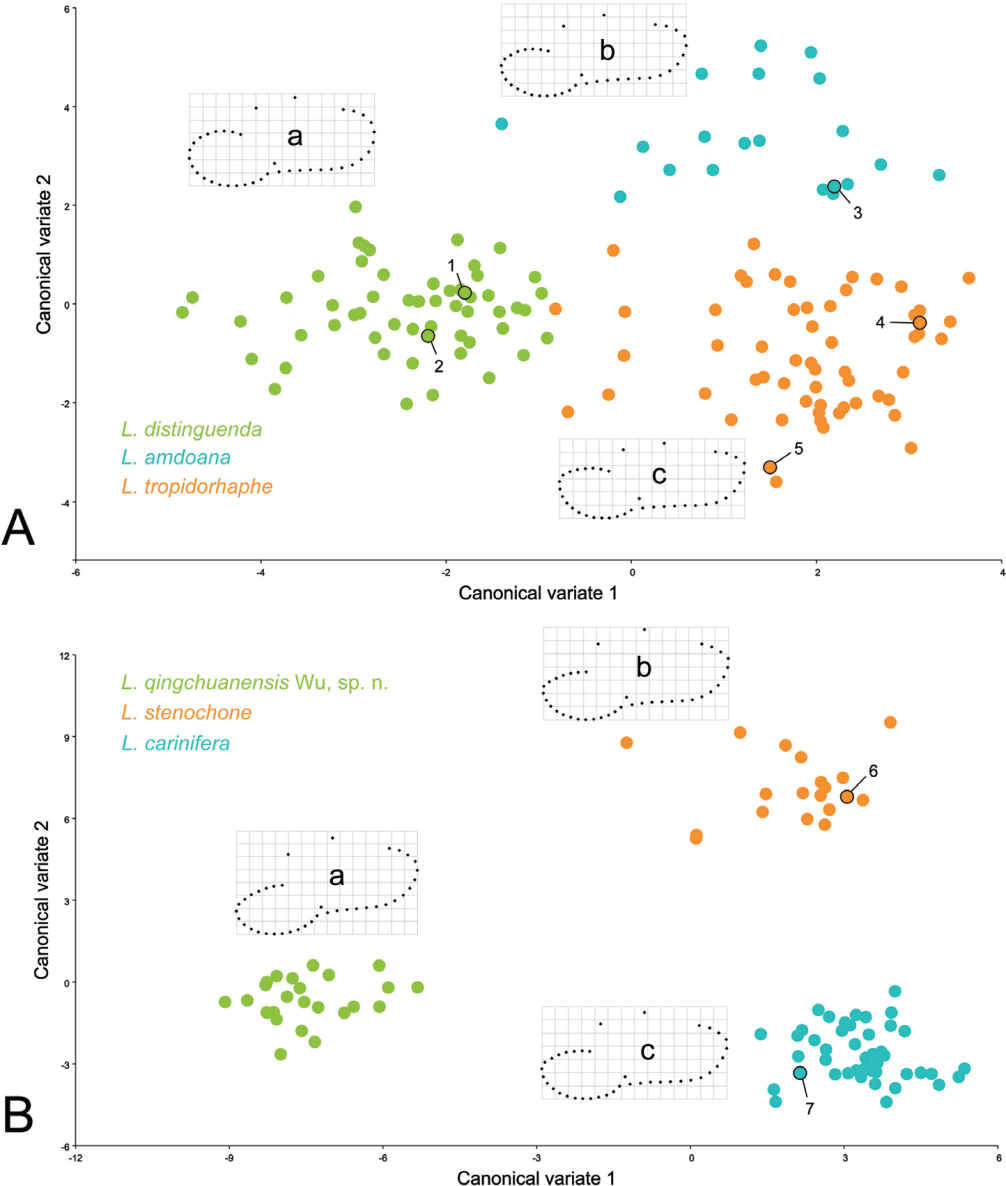
Scatter plots of canonical variate 1 against canonical variate 2 (Canonical Variate Analysis), showing the shell morphological relationships among **A***Laeocathaicadistinguenda* Möllendorff, 1899 (green dots; a, average shape), *L.amdoana* Möllendorff, 1899 (blue dots; b, average shape) and *L.tropidorhaphe* Möllendorff, 1899 (orange dots; c, average shape) **B***L.qingchuanensis* Wu, sp. nov. (green dots; a, average shape), *L.stenochone* Möllendorff, 1899 (orange dots; b, average shape) and *L.carinifera* (H. Adams, 1870) (blue dots; c, average shape). Encircled dots: 1, SMF 8959, lectotype; 2, HBUMM05436-spec.1; 3, SMF 8952, lectotype; 4, SMF 9074, lectotype; 5, paratype of *L.dangchangensis* Chen & Zhang, 2004; 6, SMF 9071, lectotype; 7, syntype, NHMUK 1870.7.16.7. Data for 5 from fig. 7D, 6 and 7 from fig. 12 in Páll-Gergely et al. 2022.

###### Remarks.

**Figure 51.**
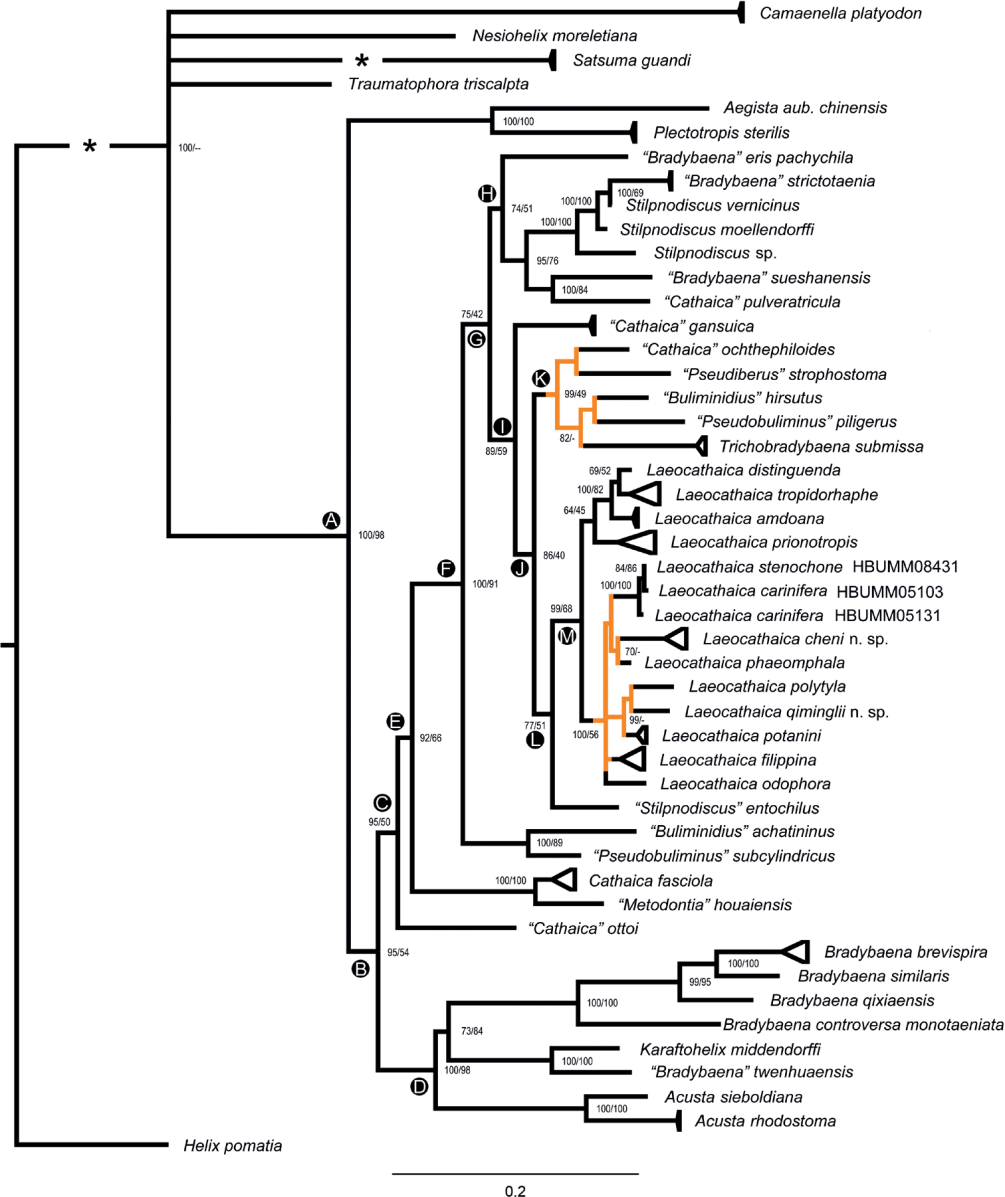
Bayesian phylogram of camaenid species (for detail see in Table 1) based on the concatenated partial mitochondrial 16S and partial ITS2 sequences. The tree is rooted with *Helixpomatia*. Numbers near nodes indicating Bayesian posterior probabilities and Maximum-likelihood bootstrap values are given as BPP ⁄BP. Black or orange part shows the topology where the result yielded by using Bayesian-Inference method agrees with that by using Maximum-likelihood method or not, respectively. An asterisk indicates the branch is exactly one third shortened in length. Scale bar is for substitutions per site.

The new species is the smallest species in *Laeocathaica*, where it is provisionally placed due to its chirality, granules on the protoconch and the similarity of the shell to that of *Laeocathaicadityla*. The very glossy shell also makes this species distinctive, reminiscent of the genus *Stilpnodiscus* Möllendorff, 1899, which appears in the phylogram (Fig. 51) as the sister group of all the *Laeocathaica* species. Undoubtedly, final generic assignment of this species will depend on further anatomical information and molecular studies.

### ﻿Phylogenetics

To investigate the systematic position of the putative *Laeocathaica* clade (because of the absence of type species of *LaeocathaicaL.christinae*), 13 species of *Laeocathaica* and 33 species of another 16 camaenid genera, whether or not they bear a dart sac, are included in the analyses (Table 3). The final dataset contains sequences from 47 species including the outgroup *Helixpomatia*. Seventy-nine of the 94 original sequences have the unique combined sequence 16S + ITS2. The model “GTR+G+I” was selected as the best nucleotide substitution model for the combined dataset (lnL = –11126.1, BIC = 24124.9).

The phylograms produced by both Maximum Likelihood and Bayesian Inference based on partial 16S and partial ITS2 sequences are topologically identical in the major branches with the exception of different positions of two nodes (orange parts in Fig. 51). The phylogenetic trees are robust that their most nodes received strong support as showed by the high Bayesian posterior probability (BPP) and/or Maximum-likelihood bootstrap values (BP) (Fig. 51). The clades *Nesiohelix*, *Satsuma* and *Traumatophora* represent the most basal offshoot in the phylogenetic trees. In Fig. 51, those clades which include the type species of a genus are recognizable by lack of quotation marks around the generic name. So, the lineage separated under Clade D for example contains a clade that includes *Helixsimilaris*, the type species of *Bradybaena*. Subsequently, species which cluster here, in fact constitute members of that genus, while those clustering elsewhere and are commonly affiliated to *Bradybaena*, have to be included in other genera by future studies. Although we did not sequence *Helixchristinae*, the type species of *Laeocathaica*, our result shows a single clade supported by BPP/bootstrap value 99/68 that includes all species considered belonging to this group. Thus, we hypothesize for the time being that this is the correct *Laeocathaica* clade.

The indigenous dart-sac-bearing camaenids aggregated in Central China, which are grouped into the genera “*Pseudobuliminus*”, “*Buliminidius*”, “*Bradybaena*”, “*Cathaica*”, “*Stilpnodiscus*”, “*Pseudiberus*”, and *Laeocathaica*, could form a strongly supported monophyletic Clade F (Fig. 51), in which the members of each of the first five genera cannot be grouped together and the branch “*Pseudiberus*” is not represented by the type species. The present phylogeny (Fig. 51) shows the following five points:

1. *Stilpnodiscus* is a polyphyletic group divided into two separated parts. The first is a monophyletic clade including “ *Bradybaena*” *strictotaenia*, which is sister to the type species of *Stilpnodiscus*. The second is “ *Stilpnodiscus*” *entochilus* Möllendorff, 1899 which is the sister group of all the *Laeocathaica* species examined here.
2. the monophyly of *Cathaica* Möllendorff, 1884 is rejected because “ *Cathaica*” *pulveratricula* (Martens, 1882), “ *Cathaica*” *ottoi* Pilsbry, 1934, *Cathaicafasciola* (Draparnaud, 1801) and “ *Cathaica*” *gansuica* (Möllendorff, 1899) occur on different branches on the tree (Fig. 51) and these species can constitute independent evolutionary units.
3. *Bradybaenacontroversamonotaeniata* Pilsbry, 1934 is confirmed as genuine *Bradybaena*, as it appears as the sister group of *B.brevispira* (H. Adams, 1870) + *B.qixiaensis* Wu & Asami, 2017 + *B.similaris* (Rang, 1831) (type of *Bradybaena*; Nordsieck 2002) on Clade D (Fig. 51), which extends the occupancy of *Bradybaena* far to the west region. The remaining “ *Bradybaena*” species occurred on Clade H and were mixed with *Stipnodiscus* and “ *Cathaica*” species.
4. Both “ *Pseudobuliminus*” and “ *Buliminidius*” are not monophyletic as indicated by the phylogram.
5. “ *Bradybaena*” *twenhuaensis* is a species of *Karaftohelix* which is a sister group of *Bradybaena*. Here, the species under the genera inside quotation marks refer to those having problematic generic assignments as indicated by this work.

## ﻿Discussion

More than 200 species belonging to 28 genera were recorded as bradybaenine or believed to be dart-sac-bearing camaenids in Chinese Mainland (Wu and Guo 2003; Schileyko 2004; Wu 2004; Chen and Zhang 2004; Wu 2019; Zhang et al. 2019; Wu et al. 2019a; Wu et al. 2019b). This work confirms that the distribution range of the least known genus *Laeocathaica* is limited to W Hubei, Chongqing, Sichuan, S Gansu, and W Shaanxi and does not extend to other adjacent regions, as shown in Fig. 2A. In this area, *Laeocathaicacarinifera*, mainly active in artificial environments, occupies the largest habitat (~ 76,500 km^2^, Fig. 2C) and seems to be the most successful species there. A previous work suggested that the parallel mountains situated at eastern Sichuan provided a series of relatively stable intermountainous environments in which populations of *Laeocathaicacarinifera* (= *L.subsimilis* in Wu and Chen 1998) exhibited divergent shell morphology, reflecting microevolution in relatively isolated environments (Wu and Chen 1998). In contrast, most other congeners of *Laeocathaicacarinifera* show a pattern of much narrower distribution. The South Gansu Plateau, a narrow region in northern Sichuan and the southern corner of Gansu, has nineteen *Laeocathaica* species that largely geographically overlap. Each species usually occupies only one or a few small areas, for extreme examples ~ 10 km^2^ for *Laeocathaicaphaeomphala* and ~ 100 km^2^ for *L.potanini* (Fig. 3), which are much more endemic in the limited local environment than for some other camaenids (e.g., *Exiligada*, Criscione et al. 2012). The variously conditioned rugged terrain of mountains and intermountainous valleys with multiple species make this region a hotspot for land snails, where the malacodiversity in this region is approximately 30 times richer than that in the rest territory of China (Wu and Xu 2013; Wu 2018).

The work of Páll-Gergely et al. (2022), in which all species in the genus *Laeocathaica* were revised, has served as an essential basis for the present study. *Laeocathaicastenochone*, treated by Páll-Gergely et al. (2022) as a synonym of *L.carinifera* based on conchological characteristics, has a much denser granulation on the embryonic shell, a higher relative shell height, and differently structured genital organ compared to the latter. The phylogram based on partial 16S and partial ITS2 data could indicate that the branch ((*Laeocathaicastenochone* HBUMM08431 + *L.carinifera* HBUMM05103) + *L.carinifera* HBUMM05131) represents a single species or two species if the problem identified by Will et al. is taken into account (Will et al. 2005: fig. 1) for species identification using the barcode protocol. The sequence data of HBUMM08431, HBUMM05103, and HBUMM05131 share the same ITS2 sequence and only differ in three sites on the 16S gene. Therefore, the present phylogenetic analyses on these three samples are identical to those obtained by a traditional barcoding method for identifying species. In the present work we therefore prefer to treat *Laeocathaicastenochone* and *L.carinifera* as two species, since they are morphologically distinguishable. Nevertheless, the present phylogram suggests that *Laeocathaicacarinifera* has a deep phylogenetic affinity to *L.stenochone*.

Until now, the systematic position of *Laeocathaica* remained an open question. Before this work, one of the two studies on the systematic position of *Laeocathaica* was based on a morphological character-based phylogram, in which *Laeocathaicacarinifera* (= *L.subsimilis*, *L.filippina* in Wu 2004) is deeply embedded and receives very weak non-homoplasious support based on that dataset (Wu 2004). Based on partial mitochondrial 16S and CO1 sequences, another work involving *Laeocathaicapolytyla* and *L.distinguenda* provided phylograms which both indicate that *Laeocathaica* shares the same robustly supported branch with *Acusta* Martens, 1860 and *Pseudobuliminus* Gredler, 1886 (Chen et al. 2021). In this second work, the monophyly of *Laeocathaica* was questioned because *Laeocathaicapolytyla* is close to *Acusta* and *Pseudobuliminus* instead of close to *L.distinguenda*, which does not agree with our work, where the *Laeocathaica* is possibly monophyletic, when more congeners (i.e., 13 *Laeocathaica* species) join the analyses based on the combined ITS2 and 16S dataset (Fig. 51).

The present phylogenetic inference is consistent with the phylogram of Wade et al. (2007), since *Satsuma* A. Adams, 1868 and *Nesiohelix* Kuroda & Emura, 1943 are basal on the phylogram, reflecting the evolutionary relationships of eastern Asian camaenids. The camaenine ingroup genus *Satsuma*, which agrees regarding its basal location on phylogram with some authors (Chiba 1999; Wade et al. 2007; Chen et al. 2021), shows a minute difference between *Nesiohelix* and *Traumatophora* Ancey, 1887 in the sequences of 16S rDNA and ITS2. The presence of a flagellum only on basal clades, represented by *Nesiohelix*, *Satsuma*, *Camaenella*, *Traumatophora*, *Aegista* Albers, 1860, and *Plectotropis* Martens, 1860, which are representatives of SE Chinese Mainland, Taiwan, Hainan, and Japan (Pfeiffer 1865; Azuma 1982; Zhou et al. 2011; Wu and Asami 2017; Wu 2019) and clearly diverge from those genera distributed in Central China (Möllendorff 1899; Chen and Zhang 2004; Wu and Prozorova 2006), agrees with that the presence or absence of the flagellum can be used as a character state coding for higher-level classification (Hirano et al. 2014; Jirapatrasilp et al. 2022). The positions of *Nesiohelix*, *Aegista*, *Acusta*, and *Bradybaena* Beck, 1837 (represented by *Bradybaena* spp. on Clade D, Fig. 51), from the basal part to the upper part, correspond topologically to the phylogenies of Wade et al. (2007). Comparing the basal part, the taxa on Clade F (Fig. 51) could present a highly evolved and diverse group among the East Asian camaenids. However, the present phylogenetic analyses suggest that *Pseudobuliminus*, *Buliminidius*, *Bradybaena*, *Cathaica*, and *Stilpnodiscus* are polyphyletic and an intensive revision is required.

**Figure 52.**
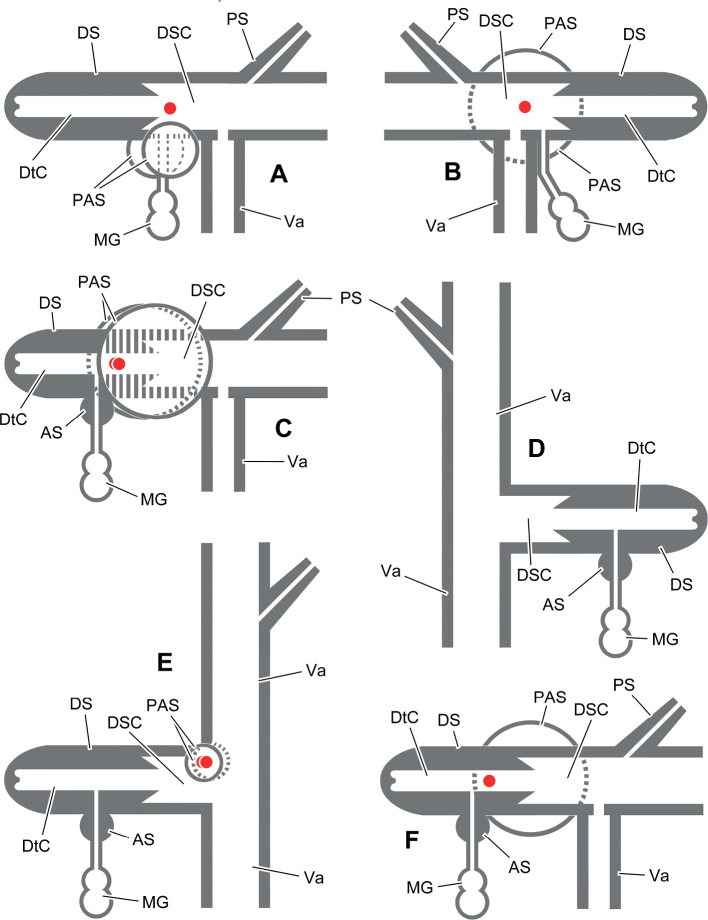
Schematics of terminal genitalia of some dart-sac-bearing camaenids **A***Cathaicafasciola* (Draparnaud, 1801) (stylized pattern from HBUMM8142-spec.1–15, 9 fma; Qingyang, Gansu, 35.738°N, 107.701°E, 1353 m a.s.l.; coll. Sheng, X.-F., 2017-VII) **B** “*Cathaica*” *pulveratricula* (Martens, 1882) (stylized pattern from HBUMM08208), one proximal accessory sac is present at the left side of dart sac **C***Laeocathaica* spp. with two proximal accessory sacs at both sides of dart sac **D***L.phaeomphala* Möllendorff, 1899, without proximal accessory sac and with elongated vagina above dart sac **E***L.amdoana* Möllendorff, 1899, with two tiny proximal accessory sacs and with elongated vagina above dart sac **F***L.dolani* (Pilsbry, 1934), with a proximal accessory sac at the right side of dart sac. Notes: any fleshy septum inside dart sac chamber is not shown; red dots indicate openings of proximal accessory sac. Abbreviations: AS – accessory sac; DS – dart sac; DSC – dart sac chamber; DtC – a chamber containing love dart; MG – mucous glands; PAS – proximal accessory sac; PR – penial retractor muscle; PS – penial sheath; Va – vagina.

Clade F (Fig. 51), which roughly corresponds to the clade containing *Acusta*, *Laeocathaica*, *Pseudobuliminus*,and *Bradybaena* in the phylogram proposed by Chen et al. (2021), is characterized by the presence of a penial sheath and a dart sac, and the absence of an epiphallic papilla, a penial caecum, or a flagellum. On Clade F, the proximal accessory sac is widely present in different subclades including *Stilpnodiscusmoellendorffi* (Wu 2001: fig. 4A, E; Wu 2004: fig. 16A, C), “*Cathaica*” *pulveratricula* (Fig. 52B), *Laeocathaica* spp. (text figures listed below most species in this study; Fig. 52C, E, F), and “*Stilpnodiscus*” *entochilus* (Wu 2004: fig. 16D, E). On Clade C, the proximal accessory sac is also present in *Cathaicafasciola*. However, the structures of the terminal genitalia including the proximal accessory sac of the taxa on Clade C are divergent. In *C.fasciola* there are two small proximal accessory sacs attaching at both lateral-ventral sides of the dart sac (Fig. 52A) while in “*Cathaica*” *pulveratricula* there is a single proximal accessory sac on the left side of dart sac (Fig. 52B). However, in both *Cathaica* species the accessory sac is absent and the mucous glands open into dart sac chamber (Fig. 52A, B) in contrast to all examined *Laeocathaica* species, where the accessory sac is present and the mucous glands open into the dart chamber via an accessory sac (Fig. 52C, E, F). The frequent occurrence of a proximal accessory sac in the subclades of Clade C indicates this structure might be one of the most valuable characters and deserving of detailed examination in respect of taxonomy and evolution in the dart-sac-bearing camaenids.

## Supplementary Material

XML Treatment for Laeocathaica

XML Treatment for Laeocathaica amdoana

XML Treatment for Laeocathaica anceyi

XML Treatment for Laeocathaica carinalis

XML Treatment for Laeocathaica carinifera

XML Treatment for Laeocathaica christinae

XML Treatment for Laeocathaica dejeana

XML Treatment for Laeocathaica distinguenda

XML Treatment for Laeocathaica dityla

XML Treatment for Laeocathaica dolani

XML Treatment for Laeocathaica filippina

XML Treatment for Laeocathaica hisanoi

XML Treatment for Laeocathaica leucorhaphe

XML Treatment for Laeocathaica minwui

XML Treatment for Laeocathaica odophora

XML Treatment for Laeocathaica pewzowi

XML Treatment for Laeocathaica phaeomphala

XML Treatment for Laeocathaica polytyla

XML Treatment for Laeocathaica potanini

XML Treatment for Laeocathaica prionotropis

XML Treatment for Laeocathaica stenochone

XML Treatment for Laeocathaica tropidorhaphe

XML Treatment for Laeocathaica parapolytyla

XML Treatment for Laeocathaica qishilii

XML Treatment for Laeocathaica qiminglii

XML Treatment for Laeocathaica zhengpingliui

XML Treatment for Laeocathaica cheni

XML Treatment for Laeocathaica qingchuanensis

XML Treatment for Laeocathaica nordsiecki
